# Novel 1,4-Dihydropyridines as Specific Binders and Activators of SIRT3 Impair Cell Viability and Clonogenicity and Downregulate Hypoxia-Induced Targets in Cancer Cells

**DOI:** 10.1021/acs.jmedchem.3c00337

**Published:** 2023-07-13

**Authors:** Clemens Zwergel, Michele Aventaggiato, Sabrina Garbo, Elisabetta Di Bello, Bruno Fassari, Beatrice Noce, Carola Castiello, Chiara Lambona, Federica Barreca, Dante Rotili, Rossella Fioravanti, Thomas Schmalz, Michael Weyand, Amelie Niedermeier, Marco Tripodi, Gianni Colotti, Clemens Steegborn, Cecilia Battistelli, Marco Tafani, Sergio Valente, Antonello Mai

**Affiliations:** † Department of Drug Chemistry and Technologies, Sapienza University of Rome, Piazzale Aldo Moro 5, 00185 Rome, Italy; ‡ Department of Experimental Medicine, “Department of Excellence 2023-2027”, Sapienza University of Rome, Viale Regina Elena 324, 00161 Rome, Italy; § Department of Molecular Medicine, “Department of Excellence 2023-2027”, Sapienza University of Rome, Viale Regina Elena 324, 00161 Rome, Italy; ∥ Dompé Farmaceutici S.p.A, Via Campo di Pile snc, 67100 L’Aquila, Italy; ⊥ Dept. Organic Chemistry, University of Bayreuth, Universitätsstr. 30, 95447 Bayreuth, Germany; # Dept. Biochemistry, University of Bayreuth, Universitätsstr. 30, 95447 Bayreuth, Germany; ¶ Institute of Molecular Biology and Pathology, Italian National Research Council, Piazzale A.Moro 5, 20, 00185 Rome, Italy; ∇ Pasteur Institute, Cenci-Bolognetti Foundation, Sapienza University of Rome, P.le A. Moro 5, 00185 Rome, Italy

## Abstract

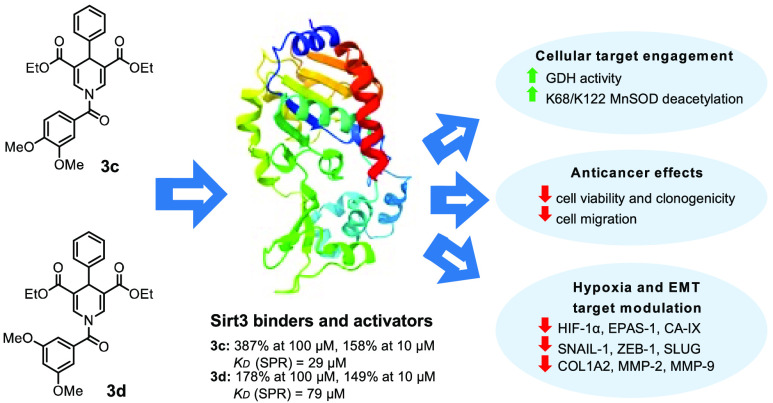

The mitochondrial SIRT3 modulates several biological pathways such as cancer, metabolism, and hypoxia-related diseases. Recently, we discovered new 1,4-dihydropyridines, compounds **2** and **3**, the latter being a SIRT3-specific activator. In the present work, a novel **2**- and **3**-related small series of compounds have been developed, with **3c** displaying the strongest SIRT3 binding and activation, with a *K*_D_ of 29 μM and 387% of enzyme activation. Differently, **3d** was the best in enhancing glutamate dehydrogenase activity and deacetylating K68- and K122-acMnSOD in triple-negative MDA-MB-231 breast cancer cells. Tested in CAL-62 thyroid cancer and MDA-MB-231 cells, **3d** displayed the strongest time- and dose-dependent reduction of cell viability and clonogenicity at a single-digit micromolar level, along with cell death, in both normoxia and hypoxia conditions. Moreover, **3d** downregulated not only hypoxia-induced factors, such as HIF-1α, EPAS-1, and CA-IX, but also epithelial–mesenchymal transition master regulators and extracellular matrix components such as SNAIL1, ZEB1, SLUG, COL1A2, MMP2, and MMP9, markedly hampering MDA-MB-231 cell migration.

## Introduction

The sirtuins family is composed of NAD^+^-dependent lysine deacetylases that, in humans, contains seven members (SIRT1–7) and belong to the class III histone deacetylases.^1−3^ The 275-amino acid catalytic core is highly conserved within the SIRT family, but their C- and N-terminal domains differ in length and sequence. Sirtuins display isoform-specific substrate affinity because of slender differences in their peptide substrate binding sites.^4^

Sirtuins have gained increasing attention over the past two decades due to their crucial roles in several biochemical contexts, including cell cycle progression, inflammation, energetic metabolism, apoptosis, neuro- and cardioprotection, and cancer onset and progression.^5−7^ Among SIRTs, SIRT1–3 mainly are deacetylating enzymes, whereas SIRT4 and SIRT6 own a substrate-specific mono-ADP-ribosyltransferase activity other their deacetylase and deacylase activities. SIRT5 can deacylate succinyl-, glutaryl-, and malonyl-lysine residues. Finally, for SIRT7, a new desuccinylase activity in addition to the previously known deacetylase one has been discovered.^8^

In general, sirtuins are regulated by post-translational modifications, connections with endogenous molecules and protein–protein interactions, and their physiological activity is controlled transcriptionally and by regulation of their degradation.^9^

In more detail, SIRT3 is predominantly located in the mitochondrial matrix and has a major role in numerous mitochondrial metabolic processes, such as the tricarboxylic acid (TCA) cycle, the urea cycle, amino acid metabolism, fatty acid oxidation, mitochondrial electron transport chain (ETC)/oxidative phosphorylation (OXPHOS), reactive oxygen species (ROS) detoxification, mitochondrial dynamics, and the mitochondrial unfolded protein response (UPR).^10,11^ In addition to its role in regulating metabolism-dependent diseases, SIRT3 displays a double-sided function in cancer development.^12^

Indeed, SIRT3 in cancer exerts a context-dependent role,^13^ being tumorigenic in some cancer types, tumor suppressor in others.^14−16^ One of the most studied oncogenic pathways concerns the regulation of hypoxia-inducible factor-1α (HIF-1α), a key transcription factor activating several glycolytic genes involved in the “Warburg effect”. SIRT3 regulates the HIF-1α activity by direct deacetylation and activation of prolyl hydroxylase (PHD), thus leading to HIF-1α ubiquitination and proteasomal degradation.^17^ Another study proved that SIRT3 overexpression destabilized HIF-1α in hypoxic human breast cancer cells, where the SIRT3 catalytic activity was needed for the complete repression of HIF-1α target genes.^15^ SIRT3 can deacetylate and modulate several targets directly or indirectly involved in glycolysis regulation leading to anticancer effects. Indeed, SIRT3 has been reported to regulate the pyruvate dehydrogenase complex (PDC) in lung cancer,^18^ cyclophilin D in breast cancer,^19^ glutamate oxaloacetate transaminase 2 (GOT2) in pancreatic cancer,^20^ and manganese-dependent superoxide dismutase (MnSOD) in chronic lymphocytic leukemia (CLL)^21^ and in hepatocellular carcinoma (HCC).^22^

Differently, to date, rather little literature evidence and even mixed reports are available for the role of SIRT3 in differentiated thyroid cancer (DTC). First, older evidence showed that SIRT3 was highly expressed in DTC compared to benign thyroid tumors,^23^ while Wang et al. showed that MiR-1225-5p might trigger DTC cell proliferation and metastasis by targeting SIRT3.^24^ Newer findings by Yao and Wang, in a more clinical setting, indicate the opposite, showing that mRNA expression levels of SIRT3 decreased in their DTC model compared to healthy cells and that patients with high SIRT3 expression had a longer disease-free survival.^25^

SIRT3 is not only involved in cancer but also in infectious,^26,27^ heart,^28^ kidney,^29^ metabolic,^30,31^ and neurodegenerative diseases such as Alzheimer’s, Parkinson’s, and Huntington’s disease, as well as in stroke and traumatic brain injury.^32^ A recent study by Yan et al. stated that SIRT3 is very likely to play a crucial role in the development of neuropathic pain.^33^

On these bases, SIRT3 activation would be a fascinating approach for the treatment of several diseases, including cancer.

**Figure 1:**
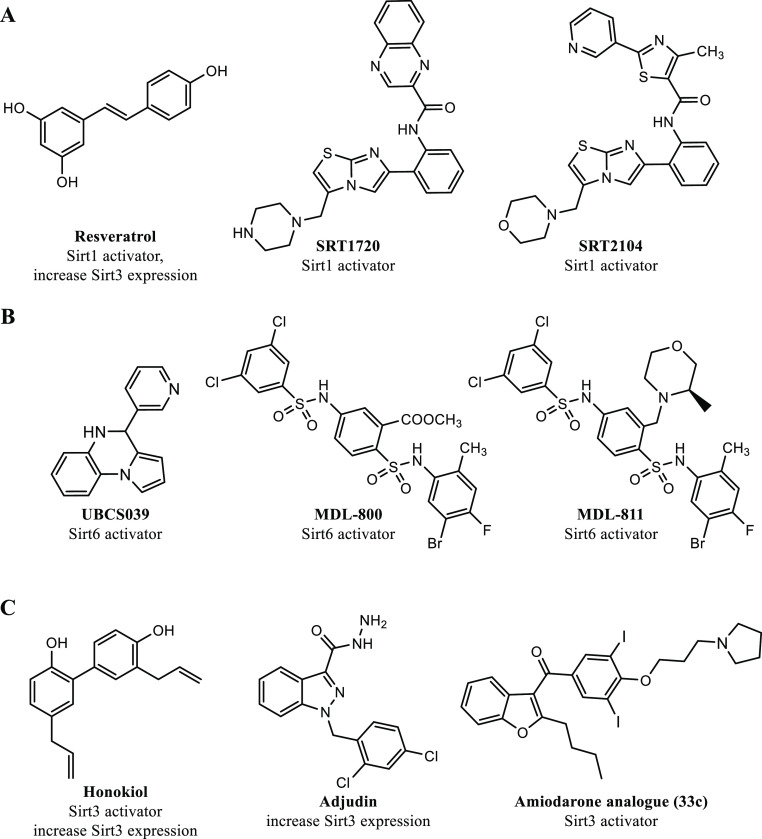
Known human sirtuins activators. (A) SIRT1 activators; (B) SIRT6 activators; and (C) SIRT3 activators.

To date, several activators of sirtuins have been developed, with particular emphasis on SIRT1.^34^ Among these, resveratrol (Figure 1A) has been shown to activate sirtuins and demonstrated promising results in a phase II clinical trial involving patients with type 2 diabetes and coronary heart disease.^35^ Its effects are limited by the poor bioavailability, but different studies are underway to try to improve it.^36^ Due to the polyphenolic nature of resveratrol, the biological activity of this molecule may be correlated with a wide range of pleiotropic modes of action, which has spurred the identification and development of more potent sirtuin-activating compounds (STACs), such as SRT1720 and SRT2104 (Figure 1A). Among STACs, SRT2104 is the most extensively studied SIRT1 activator, with various clinical trials demonstrating its anti-inflammatory, anticoagulant,^37^ anti-cholesterol,^38^ and antipsoriasis^39^ effects.

The discovery that the free 14–18 carbon-containing fatty acids act as weak SIRT6 activators has spurred the development of SIRT6 activators.^43,44^ UBCS039 (Figure 1B) activates SIRT6 in the low-micromolar range and inhibits the proliferation of HCC cells, as well as suppresses tumor growth in a rodent xenograft model.^45^ MDL-800 (Figure 1B) inhibits the proliferation of 12 non-small-cell lung cancer cell lines^46^ and suppresses tumor growth in a rodent xenograft adenocarcinoma model.^47^ MDL-811 (Figure 1B) is another SIRT6 activator that exhibits twice the activity of MDL-800 and has antiproliferative effects in colorectal cancer (CRC) cells. Additionally, MDL-811 inhibits CRC growth in patient-derived organoids, in a patient-derived xenograft, and in a spontaneous CRC mouse model, enhancing vitamin D3 anticancer activity.^48^

As briefly outlined above, small synthetic molecules have been mainly described as activators of SIRT1^34,35,37−41^ and SIRT6,^43,45−51^ while the development of potent small molecules as SIRT3 activators is still in its infancy. Different positive modulators of SIRT3 have been reported in literature, able to induce SIRT3 expression or its enzymatic activity. Noteworthy, the natural lignan honokiol (Figure 1C) has been extensively studied as a SIRT3 activator and was reported to SIRT3-dependently block cardiac fibroblast proliferation and differentiation, and finally to ameliorate cardiac hypertrophy in mice.^52^ Moreover, honokiol proved to stimulate SIRT3 activation to hamper the NF-κB/TGF-β1/Smad-regulated inflammation and fibrosis signaling in a renal fibrosis mouse model.^53^ In addition, metformin, a well-known AMPK activator used for the first-line therapy of type 2 diabetes, has been proven to SIRT3-dependently reduce atherosclerosis in type 2 diabetes patients.^54^ Adjudin (Figure 1C) proved to protect rodent cochlear hair cells against gentamicin ototoxicity via the SIRT3-ROS pathway in both in vitro and in vivo studies.^55^ The serotonin-derived pineal hormone melatonin has been found to increase SIRT3 expression and to play a protective role in heart disease, liver, and atherosclerosis.^56^ Lastly, caffeine was also described to interact with SIRT3 and induce its enzymatic activity, thus leading to K68 and K122 MnSOD deacetylation and activation.^57^ At the end of 2021, through a structure-guided design and high-throughput screening, the Liu’s research group discovered an amiodarone-derived small-molecule, compound **33c** (Figure 1C), as a specific activator of SIRT3. Indeed, proliferation and migration of human triple-negative breast cancer MDA-MB-231 cells were hampered in vitro and in vivo by compound **33c** through SIRT3-mediated autophagy/mitophagy signaling cascades.^58^

## Results and Discussion

**Figure 2:**
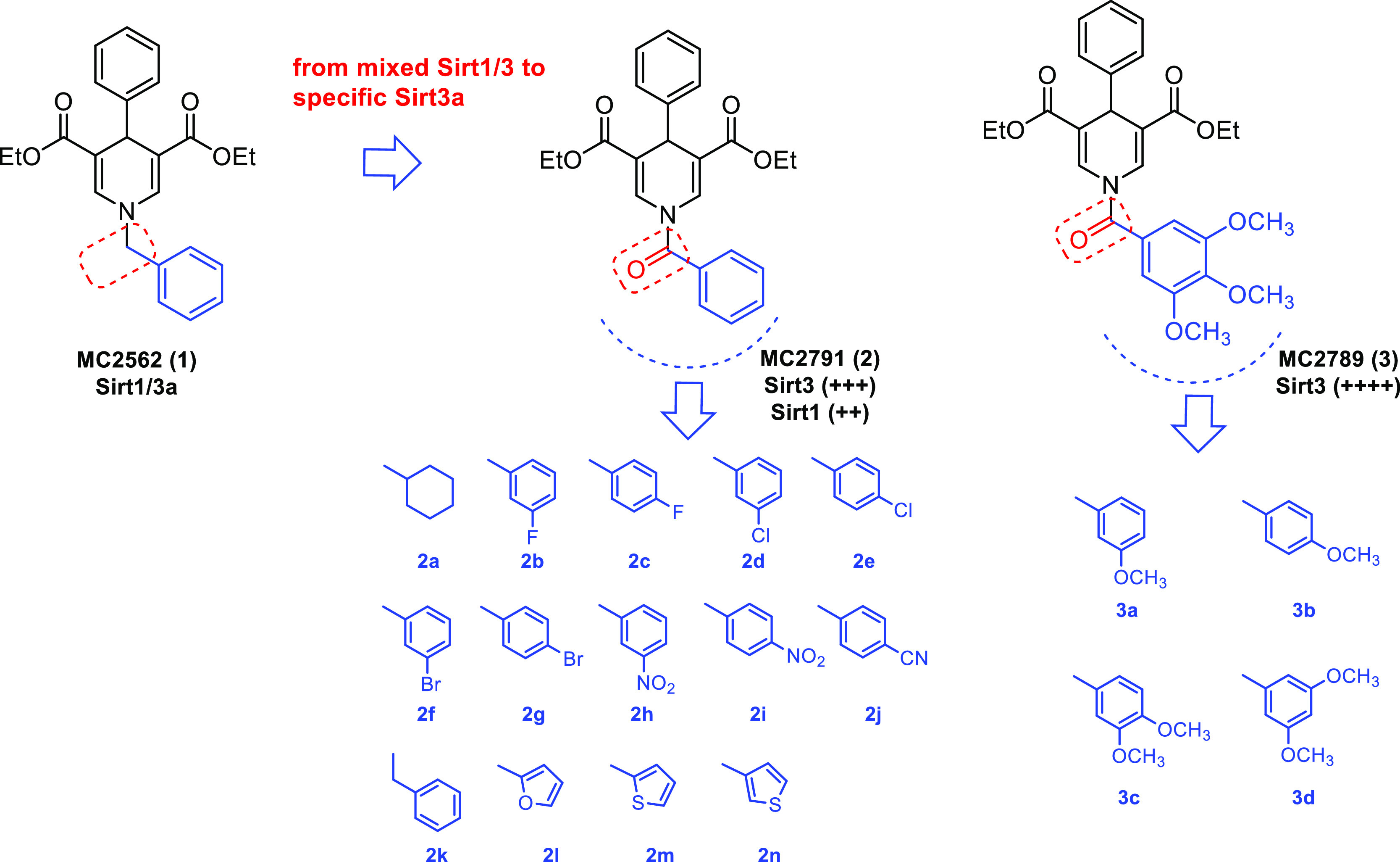
Design of novel 1,4-DHP-based SIRT3 activators **2a–n** and **3a–d** starting from the first identified hit **1**.

Since 2009 our research group has been working on the 1,4-dihydropyridine (1,4-DHP) scaffold as SIRT activators,^40−42^ identifying compound **1** (MC2562, Figure 2) as a first hit compound. Very recently, we have described the characterization of novel 1,4-DHP-based compounds that act as specific activators of SIRT3 or SIRT5.^59^ The latter bind to the catalytic cores independently from the NAD^+^ co-factor and the relative acetylated substrates and lead to an increase of the peptide or protein substrate turnover. Among them, compound **2** (MC2971, Figure 2), bearing at N1-DHP position a benzoyl moiety, proved a mixed SIRT1/3 activation providing stronger activation of SIRT3 (over 300% at 100 μM) than SIRT1 (below 200% at 100 μM), whereas compound **3** (MC2789, Figure 2), bearing a 3,4,5-trimethoxybenzoyl moiety, displayed the highest SIRT3 specific activation (over 400% at 100 μM).^59^ The SIRT3 activation by **3** was also confirmed by an MS-based assay, and both **2** and **3** exhibited SIRT3-driven cellular effects.^59^ Indeed, in MDA-MB-231 cells, **2** and **3** increased the activity of glutamate dehydrogenase (GDH), a SIRT3 substrate activated by deacetylation, and **2** proved to deacetylate the acK68 residue of MnSOD^59^ in the same cell line.

Based on these findings, we developed a novel 1,4-DHP-based series as analogues of the SIRT3 activators **2** and **3** through the following approaches: (a) by adding different substituents including halogens, nitro, and cyano groups at *meta* or *para* (*ortho* was known to be unfavorable by previous SAR data^40,41,59^) position of the N1-benzoyl ring; (b) by replacing the phenyl ring of the N1-benzoyl moiety with its sp^3^ carbon analogue (cyclohexane) or some its isosteres (furan and thiophene); and (c) by introducing a methylene spacer into the N1-benzoyl moiety to provide the corresponding N1-phenylacetyl derivative (see compounds **2a–n**, Figure 2). Also, some simplified analogues of **3** obtained by removing one by one the methoxy substituents from the 3,4,5-trimethoxybenzoyl moiety were prepared (**3a–d**, Figure 2). All novel compounds were tested against human SIRT3, and the selected **3a**, **3c**, and **3d** were also tested against human SIRT1, -2, and -5 to assess their SIRT3-specificity. Cellular functional assays for proving SIRT3 activation were performed by detecting GDH activation and changes in MnSOD acetylation levels in MDA-MB-231 cells. Moreover, the effects of selected SIRT3 activators on MDA-MB-231 and thyroid CAL-62 cancer cell viability and colony formation, as well as modulation of hypoxia-induced targets (i.e., HIF-1α, EPAS-1, and CA-IX) upon normoxia and hypoxia conditions were assessed. Furthermore, the expression of epithelial–mesenchymal transition (EMT) master regulators and of extracellular matrix (ECM) components, along with the cell migration, were analyzed upon MDA-MB-231 cells treatment with the selected compounds.

### Chemistry

**Scheme 1:**
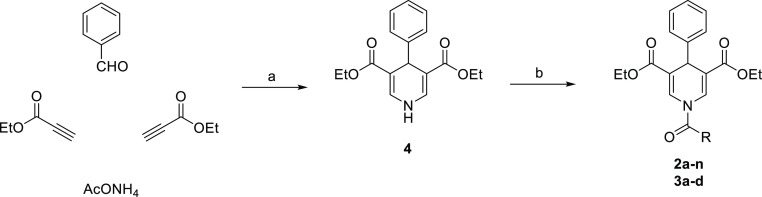
Reagents and conditions: (a) glacial acetic acid, 80 °C, 5 h; (b) Et_3_N, acyl chloride, dry dichloromethane, rt, overnight.

The key intermediate diethyl 4-phenyl-1,4-dihydropyridine-3,5-dicarboxylate (**4**) has been prepared via a multicomponent cyclocondensation between benzaldehyde, ethyl propiolate, and ammonium acetate at 80 °C in glacial acetic acid as previously described by us.^41^ Next, compound **4** underwent N1-acylation using triethylamine and the appropriate acyl chloride in dry dichloromethane to furnish the final compounds **2a–n** and **3a–d** (Scheme 1).

### Sirtuins Biochemical Assay

#### Nicotinamidase (PncA)/GDH-Coupled Deacylation Assays for Sirt1, -2, -3, and -5 Activation

To avoid artifacts and other non-specific effects, instead of the “Fluor-de-Lys” (FdL) substrate, we used a PncA/GDH-coupled deacylation assay by measuring the NADPH oxidation to NADP^+^ that relies on isoform-specific non-modified physiological acetyl or succinyl substrates [p53-acK381 for SIRT1, α-tubulin-acK25 for SIRT2, acetyl-CoA synthetase 2 (ACS2)-acK642 for SIRT3, and CPS1-succK537 for SIRT5].^59^ The assays were carried out using the 1,4-DHP derivatives **2a–n** and **3a–d** against SIRT3, including the prototype compounds **2** and **3** as positive controls.

**Figure 3:**
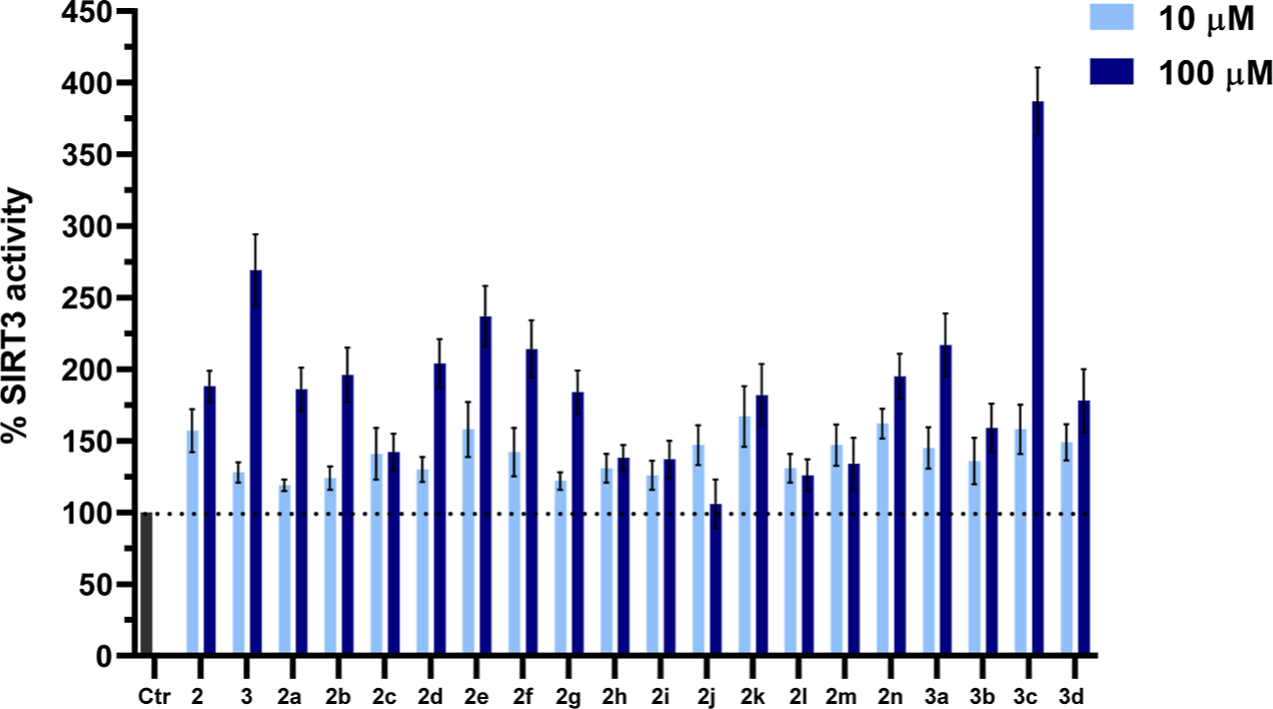
SIRT3 biochemical activation by novel 1,4-DHP derivatives **2a–n** and **3a–d** at 10 and 100 μM. Compounds **2** and **3** were used as positive controls.

As shown in Figure 3, all the novel compounds **2a–n** and **3a–d** displayed SIRT3 activation at 10 μM with values from 120% (cyclohexanoyl derivative, **2a**) to 176% (phenylacetyl derivative, **2k**), thus showing generally similar potency as the reference compounds **2** (157%) and **3** (128%) at this dose. When tested at 100 μM, the prototypes **2** and **3** confirmed their strong SIRT3 activation (188 and 269%, respectively). Among the **2a–n** analogues, **2a**, **2b**, **2d–g**, **2k**, and **2n** displayed activation potencies similar as or higher than **2** at 100 μM, with **2d–f** being the most potent, highlighting the importance of halogen insertion at the *meta* (preferred) or *para* position of the benzoyl moiety. Indeed, while the insertion of a fluorine (**2b**, 196%) or bromine (**2f**, 214%) atom at the *meta* position provided a major potency than the *para* position (**2c**, 142% or **2g**, 184%, respectively) in activating SIRT3, a chlorine atom displayed an opposite behavior (*meta***2d**, 204% vs *para***2e**, 237%), the *para*-chloro analogue **2e** being the most potent among all halogens-containing compounds **2b–g**.

Moreover, the replacement of the phenyl with the 3- (but not 2-) thienyl ring at N1 furnished **2n** with a slight improvement of the potency with respect to **2**. The systematic removal of methoxy groups by **3** (compounds **3a–d**) generally provided a decrease of SIRT3 activation at 100 μM respect to the reference compound **3**, with the sole exception of compound **3c**, bearing the 3,4-dimethoxy substitution, which intriguingly exhibited the strongest SIRT3 activation (387%), thus being 1.4-fold more potent than the reference **3** (269%) at the same concentration. Among the other methoxy analogues, **3a** (the 3-methoxybenzoyl derivative)—despite its lower potency with respect to **3** and **3c**—was able to activate SIRT3 up to 200%.

Control reactions for compound effects on the processing of the sirtuin catalysis product nicotinamide (NAM) by the coupled enzymes PncA/GDH showed negligible effects (Figure S1 in the Supporting Information), confirming that the effects of the compounds are due to direct SIRT3 activation.

Next, we selected compounds **3a**, **3c**, and **3d** to be tested against SIRT1, -2, and -5, as well, to assess their SIRT3-specificity.

**Figure 4:**
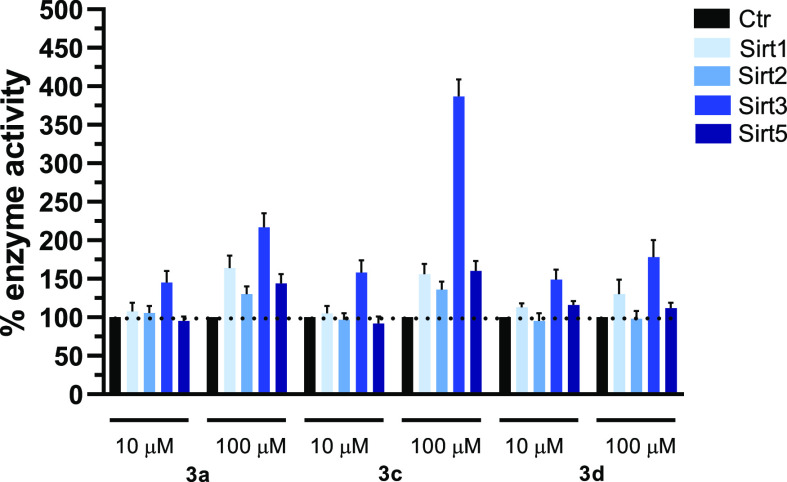
SIRT1, -2, -3, and -5 isoforms biochemical activation profile by 1,4-DHP **3a**, **3c**, and **3d** at 10 and 100 μM.

The graph in Figure 4 clearly shows that when tested at 10 μM, **3a**, **3c**, and **3d** displayed specific SIRT3 activation (145, 158, and 149%, respectively) with negligible effects on SIRT1, -2, and -5. At 100 μM, **3a** exerted 217% activation of SIRT3, joined to lower activation of SIRT1, -2, and -5 (164, 130, and 144%, respectively), while **3c** induced huge activation (387%) of SIRT3 and definitely lower activation of the other SIRT isoforms (156% (SIRT1), 136% (SIRT2), and 160% (SIRT5)), confirming a SIRT3-specific behavior. Differently, compound **3d** proved to be a mixed SIRT1/3 selective activator at 100 μM since it exhibited 130% (SIRT1) and 178% (SIRT3) enzyme activation, however displaying a major activation potency toward SIRT3.

### SIRT3 Biophysical Binding Assay

#### Surface Plasmon Resonance Assay

**Figure 5:**
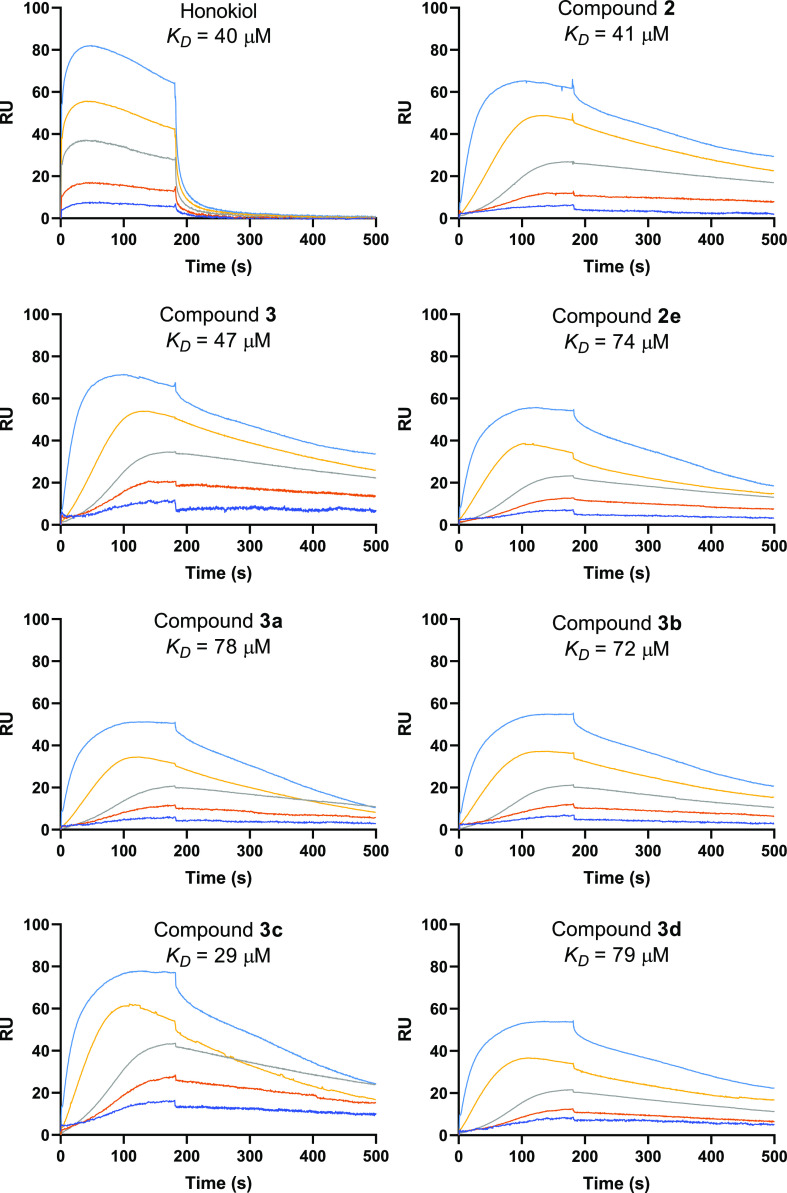
Sensorgrams of the interaction between selected SIRT3 activators **2e**, **3a–d**, reference compounds **2**, **3**, and honokiol, and SIRT3 protein immobilized onto a COOH5 sensor chip and analytes injected at the following concentrations: 5 (blue), 10 (orange), 20 (gray), 40 (yellow), and 80 μM (light blue).

Next, surface plasmon resonance (SPR) experiments were conducted with selected SIRT3 activators **2e** and **3a–d** to ascertain their biophysical interaction with the human SIRT3 protein. Their parental compounds **2** and **3**, together with honokiol, were used as positive controls. The experiments were carried out twice at different concentrations (5, 10, 20, 40, and 80 μM), by injecting the compounds at increasing doses (Figure 5). The resonance units (RU) increase with respect to baseline indicates the formation of the SIRT3/ligand complex, the plateau region represents the steady-state phase of the interaction (RUeq, RU at equilibrium) and the decrease in RU after 180 s represents dissociation of analytes from the immobilized SIRT3 protein after injection of the buffer.

Scatchard analysis showed that parental compounds **2** and **3** as well as honokiol interact with SIRT3 with *K*_D_ values ranging from 40 to 47 μM (Figure 5). Compounds **2e** and **3a–d** showed affinities in the dual digit micromolar range, with **3c** binding to the protein with the highest affinity (*K*_D_ = 29 μM) (Figure 5), consistently with its highest biochemical SIRT3 activation (387%, Figure 3).

#### Activation of SIRT3 with Physiological Substrates and in Cellular Systems

**Figure 6:**
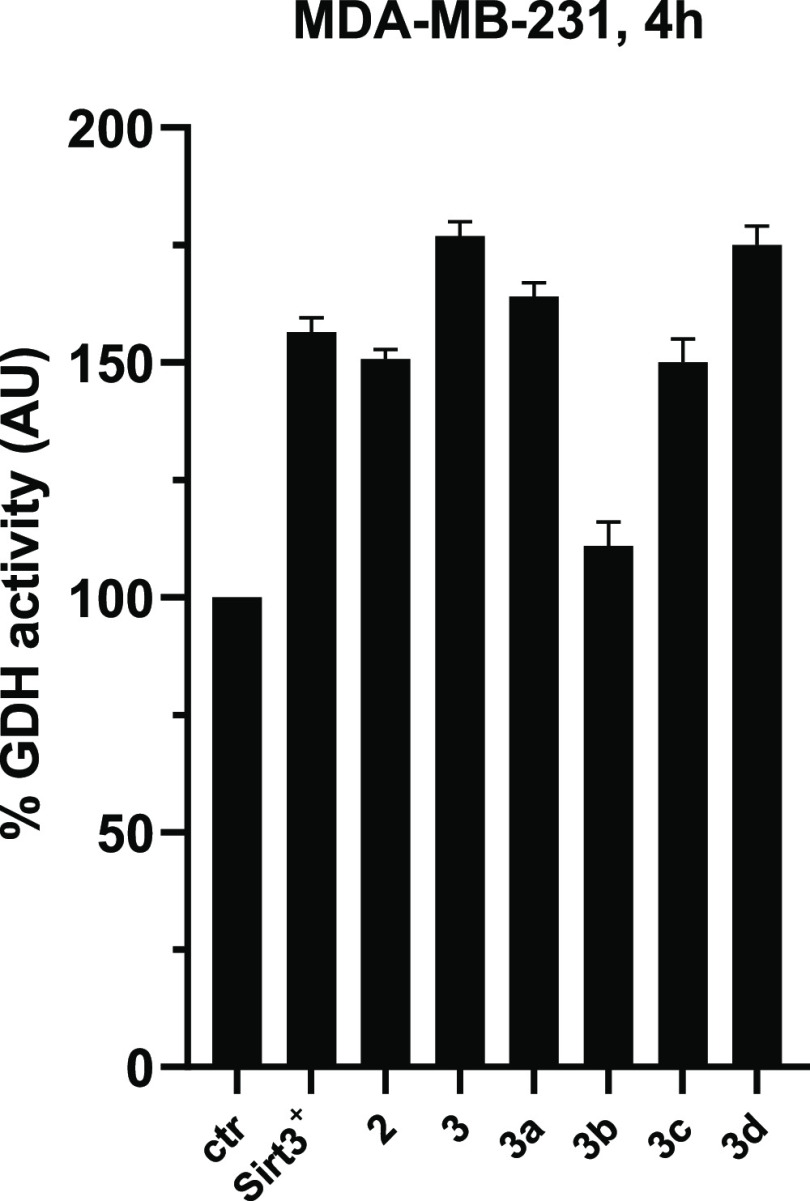
GDH activity upon 4 h treatment with SIRT3 activators **3a–d** used at 50 μM. Compounds **2** and **3** and SIRT3 overexpressing (SIRT3^+^) MDA-MB-231 cells were used as positive controls.

Selected compounds **3a–d** were tested in triple-negative breast cancer MDA-MB-231 cells to evaluate their effects on SIRT3 physiological substrates. First, the effect on the activity of GDH (an established SIRT3 substrate activated through deacetylation^60−62^) was investigated by a coupled enzyme assay. The treatment of MDA-MB-231 cells with 50 μM **3a**, **3c**, and **3d** caused an increase in GDH activity in cell lysates, detected after 4 h and consistent with SIRT3 activation, while **3b** exerted only a negligible effect, along with its weak biochemical SIRT3 activation. These effects of SIRT3 activators clearly phenocopied those observed in the SIRT3-overexpressing MDA-MB-231 cells (SIRT3^+^) (Figure 6). In detail, **3d** displayed a degree of GDH activation similar to the reference compound **3** (around 175%), and **3**, **3a**, and **3d** were even more effective than the SIRT3-overexpressing cells in increasing GDH activity (Figure 6).

#### Effects on Manganese-Dependent Superoxide Dismutase

**Figure 7:**
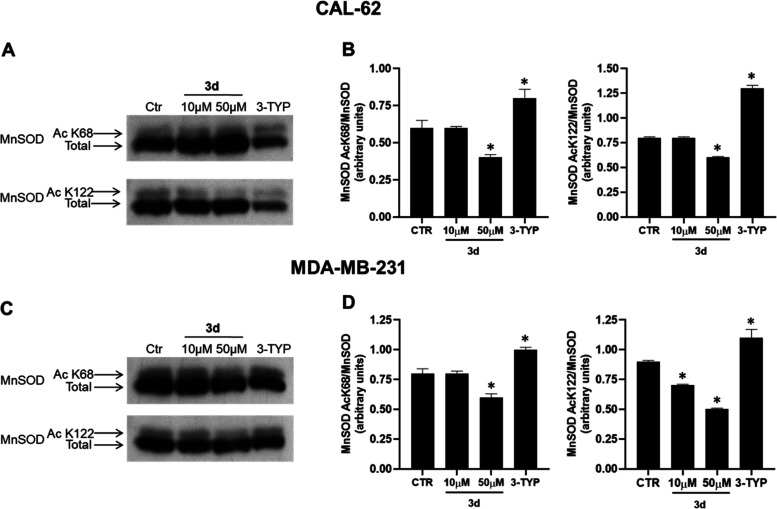
Western blotting of MnSOD acK68 and acK122 marks in CAL-62 (A) and MDA-MB-231 (C) cell lines and their respective densitometric analysis (B,D) upon treatment with 10 and 50 μM **3d** for 4 h. *p*-values were obtained using Student’s *t*-test (*p < 0.05) for three independent experiments.

The post-translational acetylation/deacetylation of MnSOD controlled by SIRT3 is demonstrated to modulate the MnSOD functions in different biological pathways.^63−67^ Among all the MnSOD lysine residues identified as targets for SIRT3 deacetylation, mainly K68 and K122 were proved to contribute to MnSOD activation.^65^ K68-MnSOD acetylation promotes a transformation-permissive phenotype, leading to a chemotherapy-resistant breast cancer cell model using cisplatin and doxorubicin.^68^ Thus, we investigated the ability of **3d**, the most potent compound to induce the SIRT3-driven GDH activation, to deacetylate the acK68- and acK122-MnSOD marks in CAL-62 and MDA-MB-231 cell lines. The specific SIRT3 inhibitor 3-TYP was utilized as negative control. As depicted in the Western blot analyses (Figure 7A,C) and relative densitometries (Figure 7B,D), **3d** at 50 μM and after 4 h treatment led to a decrease of the ratio acK68- or acK122-MnSOD/MnSOD in both the two tested cell lines, and it was able to display significant acK122 deacetylation already at 10 μM in MDA-MB-231 cells. As expected, in both cell lines, the SIRT3 inhibitor 3-TYP markedly increased the acetylation levels of both K68 and K122 residues.

#### Effects on Cell Viability and Colony Formation in CAL-62 and MDA-MB-231 Cells

**Figure 8:**
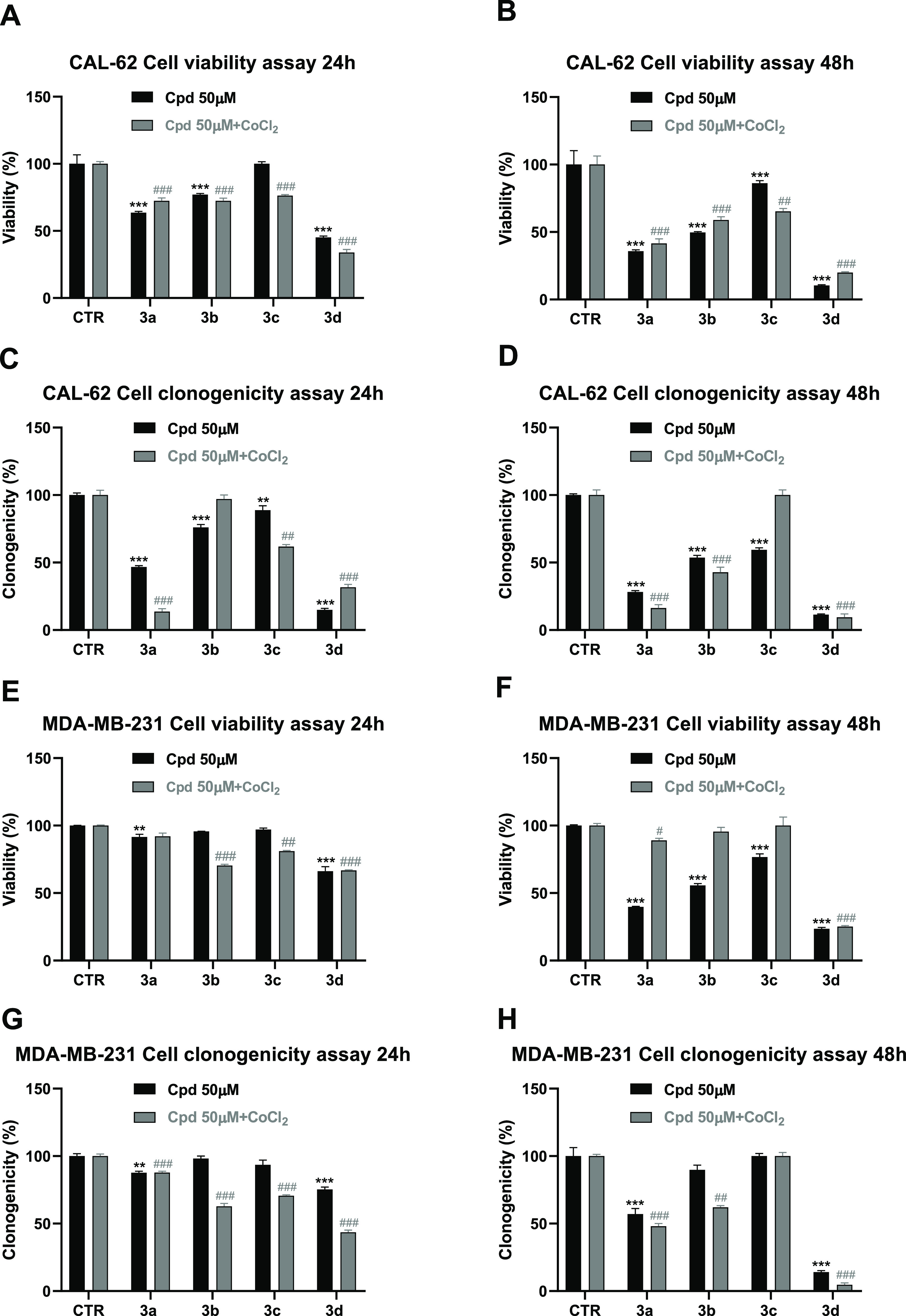
Viability and clonogenic assay under normoxia and hypoxia conditions in CAL-62 (A–D) and MDA-MB-231 (E–H) cancer cells treated with the compounds **3a–d** at 50 μM for 24 and 48 h. *p*-values were obtained using a Student’s *t*-test (*p < 0.05, **p < 0.01, and ***p < 0.001; ^#^*p* < 0.05, ^##^*p* < 0.01, and ^###^*p* < 0.001) for three independent experiments.

Afterward, the selected compounds **3a–d** were assayed at 50 μM for 24 and 48 h in CAL-62 and MDA-MB-231 cells to assess their ability to impair the cell viability and colony formation in either normoxia or hypoxia-induced condition, the latter obtained by adding a 200 μM cobalt chloride solution, as described in previous reports^69^ (Figure 8). Most cancer cells, mainly solid types, are known to have an altered metabolism due to hypoxia, a non-physiological level of oxygen tension, which contributes to therapy resistance by causing cellular quiescence.^70^ Moreover, hypoxic cancer cells can induce several factors that lead to cell proliferation and colony formation.^71^

Tested against CAL-62 cells, compounds **3a–d** provided a time-dependent reduction of viability, **3d** being the most effective (reduction of 55% at 24 h and 90% at 48 h, respectively), followed by **3a** (reduction of 37% at 24 h and 64% at 48 h) (Figure 8A,B). Under hypoxia, **3a–d** displayed similar effects as under normoxia, with **3d** (80%) and **3a** (59%) being the most potent after 48 h treatment in reducing cell viability (Figure 8A,B).

We also investigate whether our SIRT3 activators **3a–d** were able to hamper the cells’ ability to form colonies. The results in Figure 8C,D clearly show that in CAL-62 cells, **3a** and **3d** were the most effective even in these experiments, in either normal or hypoxia conditions, at both the two-time points, with a decrease of clonogenicity of 68–91% (**3d**) or 53–86% (**3a**).

When tested against MDA-MB-231 cells, after 24 h only **3d** provided weak (34%) viability impairment in both conditions. After 48 h of treatment, **3d** was the most effective (75%) in both conditions, while **3a** was more potent in normoxia (60% reduction viability) than in hypoxia (11% reduction viability) (Figure 8E,F). Even in this cell line, we tested the compounds **3a–d** toward the colony formation. In both conditions, compound **3d** displayed the strongest effect, mainly at 48 h (86 and 95% reduction of clonogenicity in normoxia and in hypoxia conditions, respectively) (Figure 8G,H). For the plate images of the clonogenic assay at 24 and 48 h, see Figure S2 in the Supporting Information.

**Figure 9:**
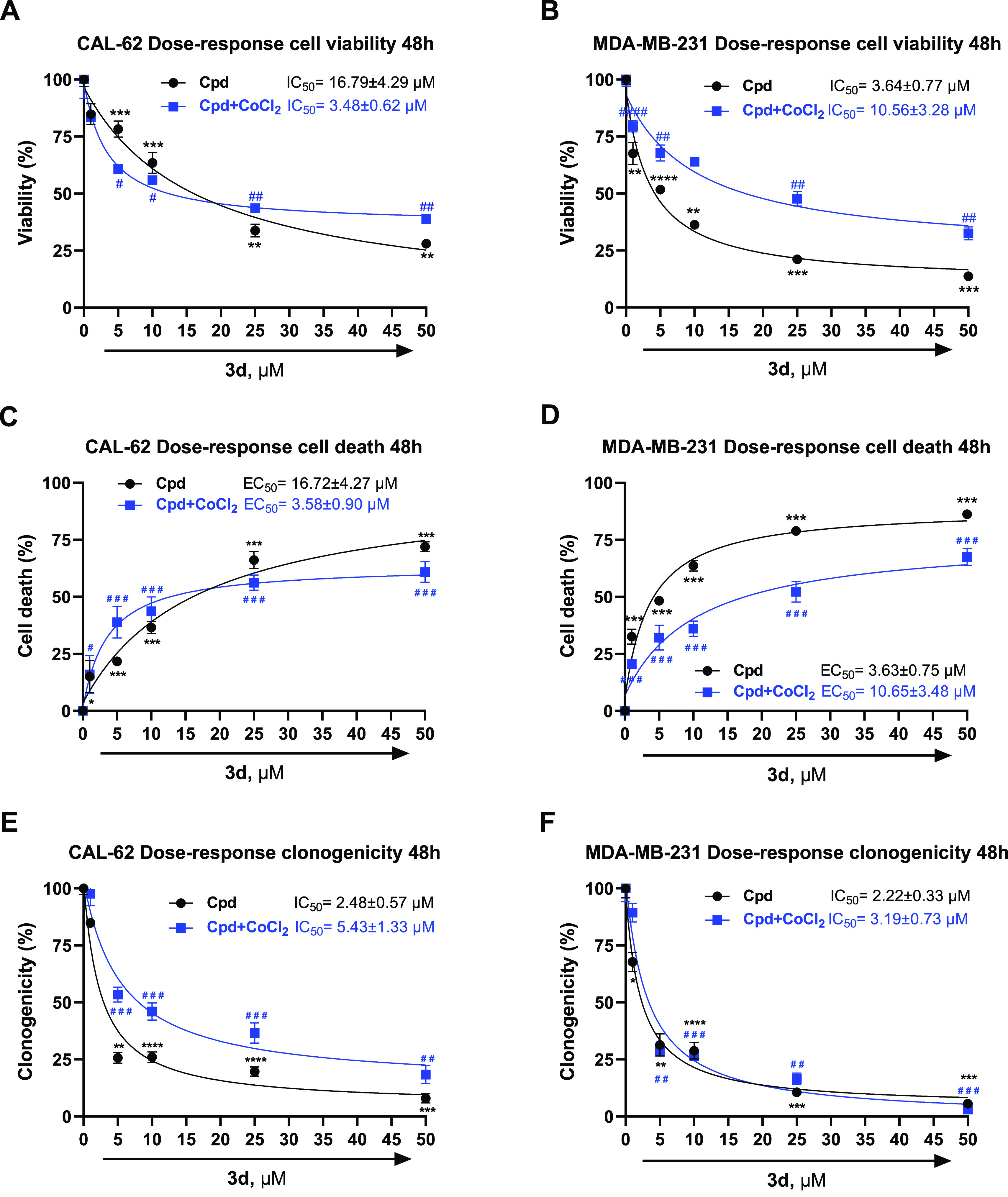
Dose-dependent effects and relative IC_50_/EC_50_ of compound **3d** on cell viability, cell death, and clonogenicity in CAL-62 (A,C,E) and MDA-MB-231 (B,D,F) cells upon 48 h of treatment. *P*-values were obtained using a Student’s *t*-test (*p < 0.05, **p < 0.01, ***p < 0.001, and ****p < 0.0001) (^#^*p* < 0.05, ^##^*p* < 0.01, ^###^*p* < 0.001, and ^####^*p* < 0.0001) for three independent experiments.

Furthermore, prompted by the auspicious results provided by the CAL-62 and MDA-MB-231 cells treatment with the SIRT3 activator **3d**, which remarkedly impaired cell viability and clonogenicity in both normal and hypoxia conditions, we decided to investigate its dose-dependent effects in the same cell lines by evaluating cell viability, cell death, and clonogenicity from 1 to 50 μM after 24 (Figure S4 in the Supporting Information) and 48 h of treatment (Figure 9), and by determining the relative IC_50_ or EC_50_ values. Compound **3d** generally displayed single-digit to low dual-digit micromolar potency in both cell lines and conditions. In detail, in CAL-62 cells, **3d** proved higher efficacy (4.8-fold) in cell viability reduction and cell death induction in hypoxia (IC_50_ and EC_50_ values around 3.5 μM) than in normoxia conditions (IC_50_ and EC_50_ values around 16.7 μM). Differently, in MDA-MB-231 cells, **3d** displayed greater potency in normoxia (IC_50_: 3.64 μM) than in hypoxia (IC_50_: 10.56 μM) in reducing cell viability, and very similar EC_50_ values between the two conditions toward cell death induction. Intriguingly, in both cell lines and in both conditions, **3d** provided single-digit micromolar IC_50_ values against colonies formation by displaying around 2.5 μM (CAL-62) and 2.2 μM (MDA-MB-231) values in normoxia and similar results in hypoxia.

The relative plate images of the dose-dependent clonogenic assay at 24 and 48 h are reported in Figure S3 in the Supporting Information.

Moreover, to assess their anticancer-specific effect, we tested **3c** and **3d**, the most potent SIRT3 activator in biochemical and in cellular assays, respectively, against healthy HaCaT cells at 24 and 48 h, using both compounds at 5, 10, and 25 μM. As reported in Figure S5 and Table S4, the compound **3c** displayed negligible toxicity at the tested doses and times, whereas the activator **3d** provided some degree of cell viability reduction and cell death only after 48 h treatment and at higher concentrations (10 and 25 μM) than its antiproliferative IC_50_ values provided in CAL-62 and MDA-MB-231 cancer cells (Figure 9).

#### Effects on HIF-1α, EPAS-1 (HIF-2α), and CA-IX Expression

In a previous study, SIRT3 was proven to be able to reprogram metabolism by destabilizing HIF-1α, a transcription factor that controls glycolytic gene expression.^72^ SIRT3 loss-dependent increase of the ROS production was resulted in HIF-1α stabilization. Furthermore, reduced SIRT3 expression in human breast cancer correlated with the increased expression of HIF-1α target genes, whereas SIRT3 overexpression impaired glycolysis and proliferation in breast cancer cells, thus unveiling a metabolic mechanism to suppress tumor growth.^15^ Moreover, a recent study showed that EPAS-1 (HIF-2α) promotes tumor growth and metastasis via EMT induction in in vitro and in vivo breast cancer models.^73^ In addition, differently from targeting HIF-1α molecules directly, another approach to hamper metastasis could be to target the carbonic anhydrase IX (CA-IX), an important hypoxia-inducible enzyme widely present in tumors downstream of HIF-1α. CA-IX is a major regulator of intra- and extra-cellular pH with a critical influence on increased survival and invasion of cancer cells.^74^ Notably, pre-clinical studies in breast cancer models showed that CA-IX inhibition led to a decrease in tumor growth and metastasis progression.^74^

**Figure 10:**
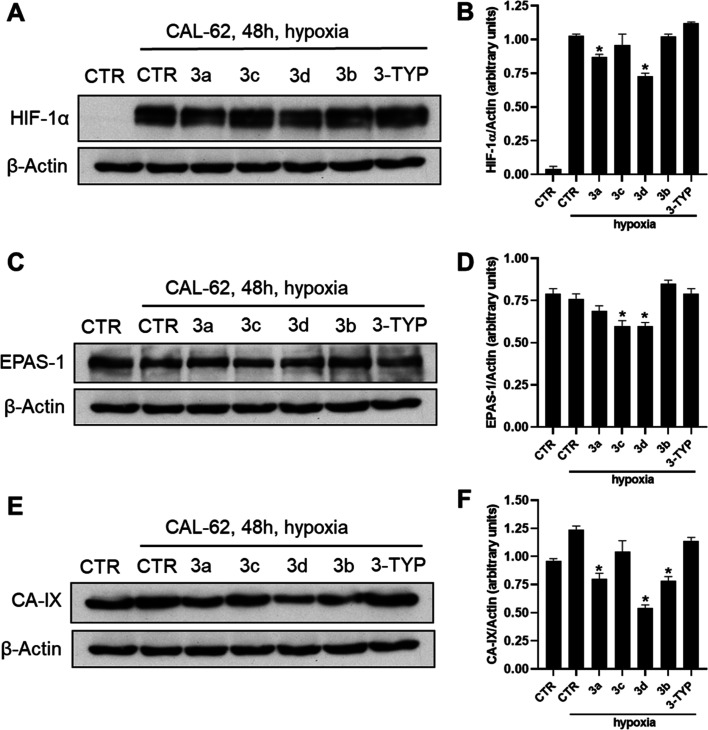
Western blotting of HIF-1α (A), EPAS-1 (HIF-2α) (C), and carbonic anhydrase IX (CA-IX) (E) and their respective densitometric analysis (B,D,F) in CAL-62 cell line upon treatment with 10 and 50 μM compounds **3a–d** for 48 h. Compound 3-TYP was used as a negative control. *p*-values were obtained using Student’s *t*-test (*p < 0.05) for three independent experiments.

**Figure 11:**
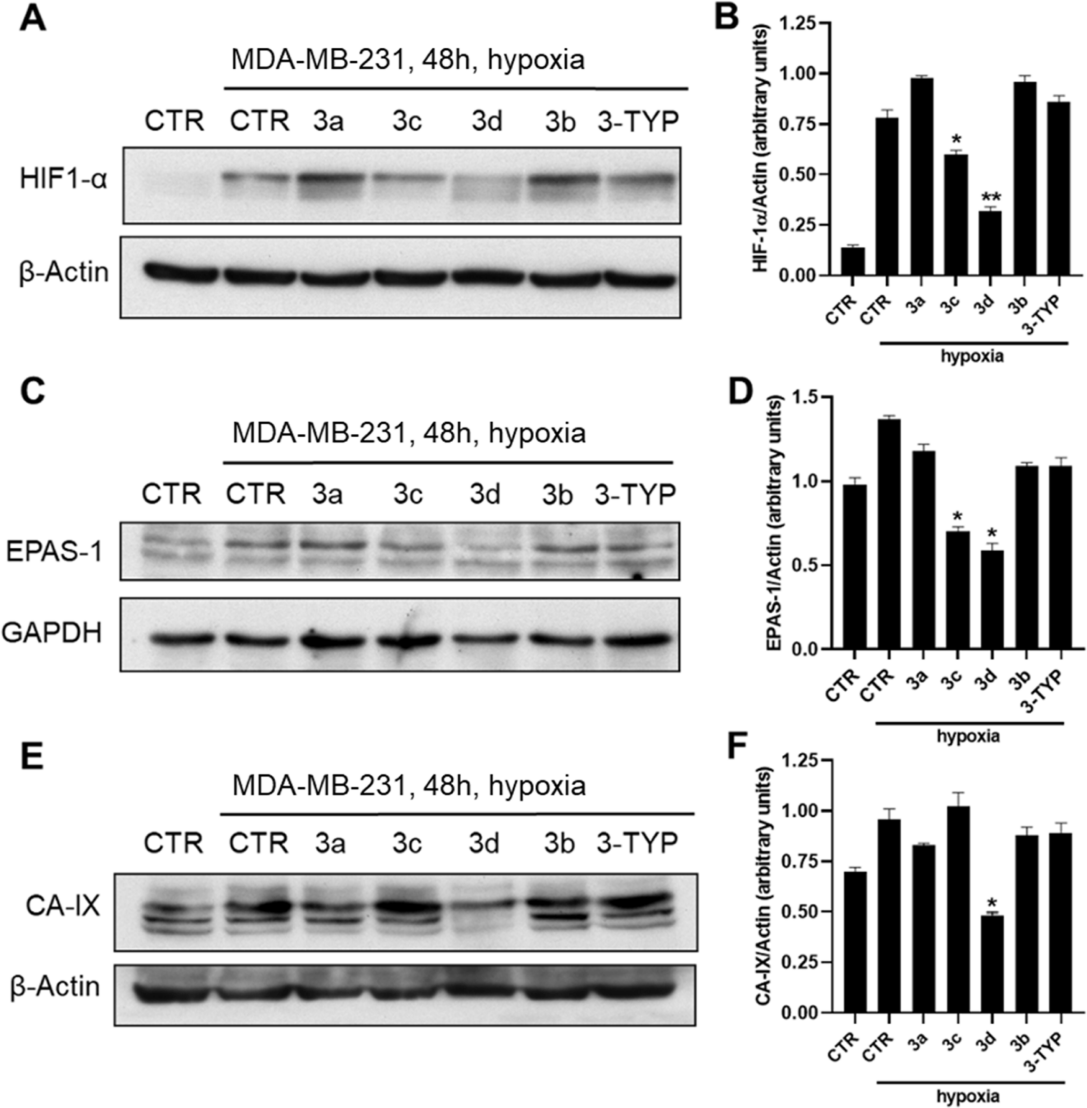
Western blotting of HIF-1α (A), EPAS-1 (HIF-2α) (C), and carbonic anhydrase IX (CA-II) (E) and their respective densitometric analysis (B,D,F) in the MDA-MB-231 cell line upon treatment with 50 μM compounds **3a–d** for 48 h. Compound 3-TYP was used as a negative control. *p*-values were obtained using Student’s *t*-test (*p < 0.05, **p < 0.01) for three independent experiments.

Based on these findings, we decided to assess the ability of the SIRT3 activators **3a–d** to impair HIF-1α, EPAS-1, and CA-IX protein expression in CAL-62 (Figure 10A–F) and MDA-MB-231 (Figure 11A–F) cell lines treated with the compounds at 50 μM for 48 h and under hypoxia conditions. As shown by the western blotting performed on CAL-62 cells (Figure 10), compounds **3d** and, to a lesser extent, **3a** were able to decrease HIF-1α, whereas EPAS-1 protein was downregulated by **3c** and **3d**, and the CA-IX protein was significantly downregulated by compounds **3a**, **3b**, and **3d**. Overall, compound **3d** was confirmed to be the most effective in the modulation of all three proteins. Moreover, the SIRT3 inhibitor 3-TYP displayed negligible effects in all the experiments described above.

Next, we evaluated the effects of **3a–d** on HIF-1α, EPAS-1, and CA-IX in MDA-MB-231 cells, as well (Figure 11A–F). Compounds **3c** and **3d** significantly reduced HIF-1α and EPAS-1 protein levels, with **3d** being the most effective and providing stronger effects when compared to CAL-62 cells. Even against the CA-IX protein expression, **3d** proved to be the most potent giving a downregulation similar to that observed in CAL-62 cells. Also in this cell line, 3-TYP did not show any activity.

#### Epithelial to Mesenchymal Transition Modulation by SIRT3 Activators

**Figure 12:**
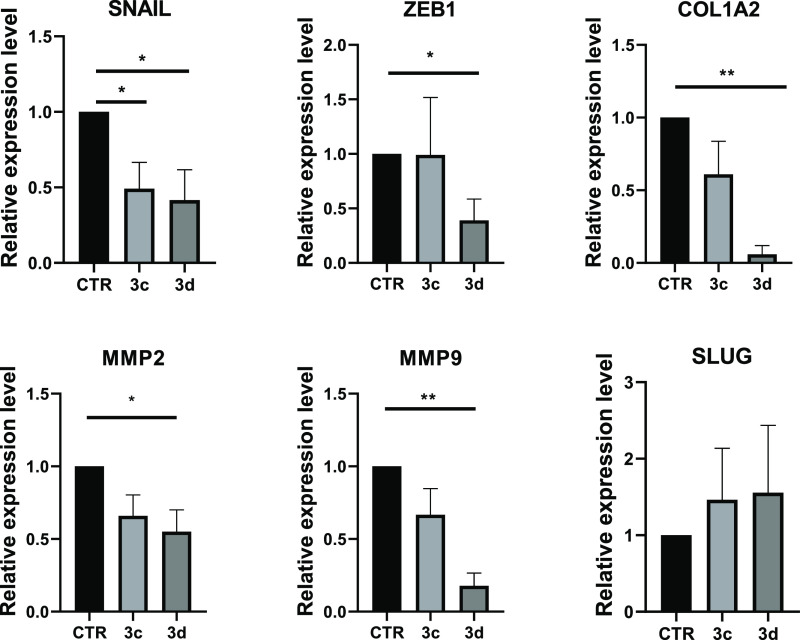
qRT-PCR analysis for the indicated transcripts in MDA-MB-231 treated with the compounds **3c,d** at 50 μM for 48 h. CTR (control) represent the cells treated with the vehicle (DMSO). The values are calculated by the 2(−Δ*Ct*) method, expressed as fold of expression vs the control (arbitrary value = 1) and shown as mean ± SEM. Statistically significant differences are reported (*, *p* < 0.05; **, *p* < 0.01) for three independent experiments.

Afterward, based on the double localization described for SIRT3, which was primarily found into the mitochondria but translocated into the nucleus in stressful conditions,^75^ the effects of SIRT3 activators on gene expression regulation were evaluated in MDA-MB-231 cells. Specifically, literature data relate SIRT3 inactivation to EMT, leading to plastic and mobile phenotype by overexpressing transcription factors such as SLUG, SNAIL, and ZEB,^76^ and prove the involvement of SIRT3 in counteracting EMT.^77^ These findings suggest a potential effect of SIRT3 activation in limiting cell migration properties. Hence, the expression of some EMT master regulator genes (i.e., SNAIL, SLUG, and ZEB1) and of ECM components (COLLAGEN and MMP2/9) was analyzed upon 48 h of treatment with selected compounds **3c** and **3d** used at 10 (Figure S5 in the Supporting Information) and 50 (Figure 12) μM.

Notably, both compounds **3c** and **3d** were able to reduce the SNAIL, COL1A2, and MMP2/9 expression levels, but only **3d** reduced the ZEB1 expression, whereas the SLUG gene was not significantly affected (Figure 12). This could be due to the multiple regulation mechanisms targeting these genes that could be independent of the SIRT3 nuclear role in this cellular context. Of note, compound **3d** displayed a stronger effect than **3c** in all cases, providing the best downregulation for COL1A2 and MMP2/9 genes.

Intriguingly, the effectiveness of these SIRT3 activators was maintained at their lower concentration (10 μM) (Figure S5 in the Supporting Information).

**Figure 13:**
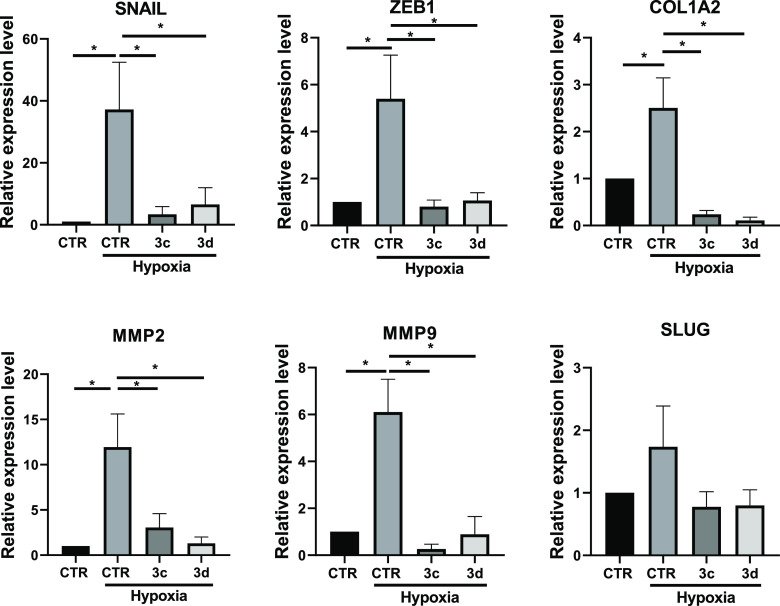
qRT-PCR analysis for the indicated transcripts in MDA-MB-231 treated with the compounds at the concentration of 50 μM for 48 h. CTR (control) represent the cells treated with the vehicle (DMSO). Hypoxia was induced by the cell treatment with CoCl_2,_ as reported in the Experimental Section. The values are calculated by the 2(−Δ*Ct*) method, expressed as fold of expression vs the control (arbitrary value = 1) and shown as mean ± SEM. Statistically significant differences are reported (*, *p* < 0.05; **, *p* < 0.01) for four independent experiments.

Considering the previously described involvement of hypoxia in EMT induction,^78^ we extended our biological study by analyzing the effects of SIRT3 activators on gene expression regulation under hypoxic conditions. Hypoxia induction of EMT was verified by gene expression analysis (upregulation of SNAIL, ZEB1, COLLAGEN, MMPs, and SLUG) after 72 h of treatment with CoCl_2_. In these experimental conditions, we verified the effectiveness of the previously tested SIRT3 activators **3c** and **3d** (50 μM, 48 h) in reducing EMT genes expression. Notably, both compounds not only maintained their ability to impair SNAIL, ZEB1, COLLAGEN, and MMP2/9 gene expression but also affected SLUG expression after 48 h of treatment in hypoxic conditions (Figure 13). Overall, **3c** and **3d** displayed a very high and stronger efficacy in hypoxia with respect to the normoxia condition. This suggests that the contribution of SIRT3 in the regulation of EMT genes is much more evident after the hypoxia induction.

#### Scratch Assay

**Figure 14:**
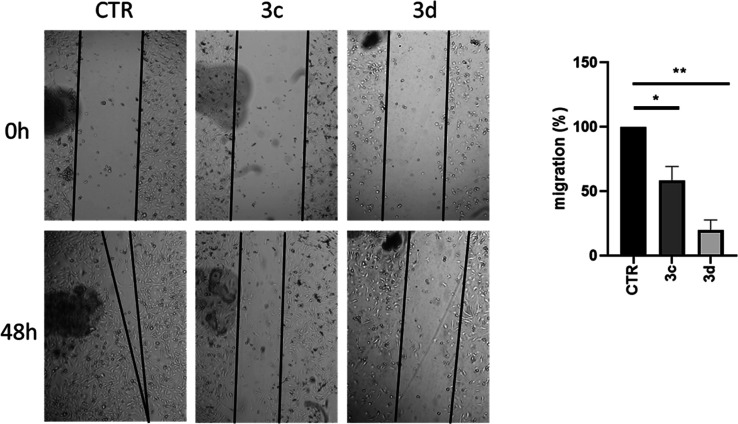
Scratch assay on MDA-MB-231 cells treated with the indicated compounds at the concentration of 50 μM for 48 h. CTR (control) represent the cells treated with the vehicle (DMSO). Left panel: phase contrast micrographs of cells at two different time points. Right panel: quantification of cell migration. Data are shown as the mean ± S.E.M. of three independent experiments. Significant results are indicated: *p* < 0.05 (*); *p* < 0.01 (**).

Next, a scratch assay on MDA-MB-231 cells after treatment with **3c** and **3d** at 50 μM for 48 h was performed to assess their effect on cell migration (Figure 14). The obtained data clearly show that compound **3d**, even in these experiments, displayed the highest effect by impairing over 75% of the MDA-MB-231 migration rate, consistently with the gene modulation effect discussed above.

## Conclusions

Very recently, our group reported that structurally different modifications on the 1,4-DHP scaffold allowed the discovery of specific SIRT3 activators, such as compounds **2** and **3**, confirmed by functional tests in cellular settings.^59^ Here, we describe the development of novel **2** and **3** analogues, compounds **2a–n** and **3a–d**, obtained by structural modification at the N1-benzoyl moiety and their biological validation as novel SIRT3-specific activators. Among the new compounds, the 3,4-dimethoxy substitution at the N1-benzoyl moiety provided the compound **3c** as the most potent and selective SIRT3 activator in biochemical activation assays (387% at 100 μM) as well as in biophysical experiments (SPR assay), with a *K*_D_ of 29 μM. Afterward, to assess the cellular target engagement, we tested compound **3c** and its analogues **3a**, **3b**, and **3d**, along with their parental compounds **2** and **3**, in triple-negative MDA-MB-231 breast cancer cells to evaluate their SIRT3-driven GDH activation. After 4 h of treatment at 50 μM, the most effective compound was **3d**, bearing the 3,5-dimethoxybenzoyl moiety at the 1,4-DHP N1 position, affording around 175% GDH activation similar to the reference compound **3**, and more effective than the SIRT3-overexpressing cells. Then, we tested **3d** for evaluating its effects on acetylation level of MnSOD, another target of SIRT3. This physiological substrate was deacetylated at the known specific residues K68 and K122 after 4 h and 50 μM treatment, markedly in breast MDA-MB-231 cells, whereas the SIRT3 inhibitor 3-TYP provided increased acetylation of the above lysine residues, as expected.

Furthermore, when **3a–d** were tested in thyroid CAL-62 and breast MDA-MB-231 cancer cells, **3a** and **3d** displayed the highest time-dependent activity reducing the cell viability and colony formation in both normoxia and hypoxia environments. Compound **3d** displayed the most potent effect in all experiments, achieving a massive impairment upon 48 h treatment in both the tested cell lines and conditions. Based on these promising results, we investigated the dose-dependent (1–50 μM) effects of **3d** in the same cell lines, wherein it displayed a nice dose–response curve reducing cell viability along the cell death, as well as the clonogenic activity, showing IC_50_/EC_50_ values range of 2.5–16.8 μM (normoxia) and 3.5–5.4 μM (hypoxia) in CAL-62 cells, 2.2–3.6 μM (normoxia) and 3.2–10.6 μM (hypoxia) in MDA-MB-231 cells. Intriguingly, the anti-clonogenic effect was the most remarkable. Moreover, since reduced SIRT3 expression in human breast cancer has been reported to lead to HIF-1α stabilization and upregulation of HIF-1α target genes, we explored the effects of compounds **3a–d** in modulation of HIF-1α, EPAS-1 (HIF-2α) and CA-IX expression in both CAL-62 and MDA-MB-231 cells after 48 h of treatment. Even in these experiments, the SIRT3 activator **3d** displayed the strongest effect, with a significant downregulation of all the three proteins mentioned above, and mainly in MDA-MB-231 cells.

Furthermore, the modulation by **3c** and **3d** of the EMT master regulator genes’ expression, such as SNAIL, SLUG, and ZEB1, and of ECM components, such as COLLAGEN and MMPs, in MDA-MB-231 cells was investigated under normoxia and hypoxia conditions. Both compounds were able to reduce SNAIL, ZEB1, COL1A2, MMP2, and MMP9 gene expression after 48 h of treatment at 50 μM, with compound **3d** being the strongest even in this investigation, while SLUG gene expression was downregulated only in hypoxia conditions. Intriguingly, compounds **3c** and **3d** were effective even at 10 μM, in agreement with the reduction of cell viability, clonogenicity, and induction of cell death observed at the same dosage. Moreover, their effects were generally and clearly stronger in hypoxia cells, underlining the important role of SIRT3 in regulating the expression of genes at least partly dependent on the hypoxia. In complete agreement with this, compound **3c** and mainly **3d** were also able to nicely reduce the MDA-MB-231 cell migration after 48 h of treatment at 50 μM.

In conclusion, the present work highlights that our SIRT3 activators are endowed with anticancer properties and could represent a tool for future in-depth studies of the SIRT3 roles and functions in cancer pathogenesis. Moreover, the DHP scaffold could be worthy of further med-chem optimization for improving its SIRT3 activation potency and selectivity.

## Experimental Section

### Chemistry

Melting points were determined on a Buchi 530 melting point apparatus and are uncorrected. ^1^H NMR and ^13^C spectra were recorded at 400 MHz on a Bruker AC 400 spectrometer; chemical shifts are reported in δ (ppm) units relative to the internal reference tetramethylsilane (Me_4_Si). All compounds were routinely checked by TLC, ^1^H, and ^13^C NMR. IR spectra were recorded on a PerkinElmer Spectrum 100 FT-IR. TLC was performed on aluminum-backed silica gel plates (Merck DC, Alufolien Kieselgel 60 F254) with spots visualized by UV light. All solvents were reagent grade and, when necessary, were purified and dried by standard methods. Concentration of solutions after reactions involved the use of a rotary evaporator operating at a reduced pressure of ca. 20 Torr. The purity of the final compounds **2a-n** and **3a-d** was analyzed by elemental analysis and HPLC. The elemental analyis has been performed on Thermo Fisher FlashSmart CNHS/O. The HPLC system consisted of a Dionex UltiMate 3000 UHPLC (Thermo Fisher) system equipped with an automatic injector and column heater and coupled with a Diode Array Detector DAD-3000 (Thermo Fisher). The analytical controls were performed on a Hypersil GOLD C18 Selectivity 5 μm (4.6 × 250 mm) HPLC Column (Thermo Fisher) in gradient elution. Eluents: (A) H2O/CH3CN, 95/5 (v/v) + 0.1% TFA; (B) CH3CN/H2O, 95/5 (v/v) + 0.1% TFA. A 20 min linear gradient elution from 30% to 100% solvent B was followed by 5 min at 100% B. The flow rate was 1.0 mL/min, and the column was kept at a constant temperature of 30 °C. Samples were dissolved in solvent B at a concentration of 0.25 mg/mL, and the injection volume was 10 μL. Analytical results are within ±0.40% of the theoretical values. All chemicals were purchased from Sigma-Aldrich Chemistry, Milan (Italy), Flurochem, Manchester (UK), or from AlfaAesar, Karlsruhe (Germany), and were of the highest purity.

The intermediate diethyl 4-phenyl-1,4-dihydropyridine-3,5-dicarboxylate (**4**) has been prepared according to the literature.^41^

The final compounds **2a–n** and **3a–d** possess a purity >95% purity confirmed by ^1^H and ^13^C NMR as well as elemental analysis and HPLC.

### General Procedure for the Synthesis of the Final Compounds **2a–n** and **3a–d**

#### Example: Synthesis of Diethyl 1-(Cyclohexanecarbonyl)-4-phenyl-1,4-dihydropyridine-3,5-dicarboxylate (**2a**)

To a solution of diethyl 4-phenyl-1,4-dihydropyridine-3,5-dicarboxylate (0.33 mmol, 0.100 g) in DCM dry (3 mL) was added TEA (1.33 mmol, 0.185 mL) and then, dropwise at 0 °C, cyclohexanecarbonyl chloride (0.36 mmol, 0.053 g) was dissolved in DCM dry (3 mL). The reaction was stirred overnight at RT. The solvent was then evaporated, and the remaining residue was purified by column chromatography (silica gel) using EtOAc/hexane (1:2) as the eluent. The product was isolated as a white solid (0.023 g, 17.0%).

##### **2a**, MC4199, Diethyl 1-(cyclohexanecarbonyl)-4-phenyl-1,4-dihydropyridine-3,5-dicarboxylate

mp 92–93 °C; yield: 17.0%; ^1^H NMR (CDCl_3_, 400 MHz, δ, ppm): δ 1.13 (t, 6H, *J* = 7.2 Hz, 2× −OCH_2_C*H*_3_), 1.18–1.36 (m, 4H, cyclohexane protons), 1.52–1.55 (m, 5H, cyclohexane protons), 1.79–1.86 (m, 2H, cyclohexane protons), 3.98–4.1 (m, 4H, 2× −OC*H*_2_CH_3_), 4.80 (s, 1H, ArC*H*−), 7.09–7.19 (m, 5H, aromatic protons), 8.03 (s, 2H, dihydropyridine protons) ppm; ^13^C NMR (100 MHz, CDCl_3_): δ 14.10 (2C), 25.41, 25.61, 29.18 (2C), 38.62, 41.11, 60.78 (2C), 115.46, 126.94, 128.18 (3C), 128.55 (3C), 129.62 (2C), 143.77, 165.96 (2C), 172.99 ppm.

##### **2b**, MC4158, Diethyl 1-(3-fluorobenzoyl)-4-phenyl-1,4-dihydropyridine-3,5-dicarboxylate

mp 124–125 °C; yield: 85.2%; ^1^H NMR (CDCl_3_, 400 MHz, δ, ppm): δ 1.21 (t, 6H, *J* = 7.2 Hz, 2×-OCH_2_C*H*_3_), 4.06–4.20 (m, 4H, 2× −OC*H*_2_CH_3_), 4.96 (s, 1H, ArC*H*−), 7,25–7,21 (m, 1H, aromatic proton), 7.28–7.44 (m, 5H, aromatic protons), 7.40–7.44 (m, 2H, aromatic protons), 7.52–7.57 (m, 1H, aromatic proton), 8.08 (s, 2H, dihydropyridine protons) ppm; ^13^C NMR (100 MHz, CDCl_3_): δ 14.08 (2C), 38.92, 60.90 (2C), 116.12, 116.49, 119.68, 124.50, 127.00, 127.13 (3C), 128.37, 128.53 (3C), 130.70 (2C), 143.44, 161.43, 165.56 (3C) ppm.

##### **2c**, MC4168, Diethyl 1-(4-fluorobenzoyl)-4-phenyl-1,4-dihydropyridine-3,5-dicarboxylate

mp 123–124 °C; yield: 93.6%; ^1^H NMR (CDCl_3_, 400 MHz, δ, ppm): δ 1.12 (t, 6H, *J* = 7.2 Hz, 2× −OCH_2_C*H*_3_), 3.97–4.11 (m, 4H, 2× −OCH_2_CH_3_), 4.88 (s, 1H, ArC*H*−), 7.14–7.27 (m, 7H, aromatic protons), 7.61–7.65 (m, 2H, aromatic protons), 8.00 (s, 2H, dihydropyridine protons) ppm; ^13^C NMR (100 MHz, CDCl_3_): δ 14.10 (2C), 38.88, 60.87 (2C), 115.79, 116.25 (2C), 116.47 (2C), 127.10, 128.36, 128.50 (2C), 131.04, 131.72 (3C), 131.82, 143.60, 161.43, 165.64 (3C) ppm.

##### **2d**, MC4172, Diethyl 1-(3-chlorobenzoyl)-4-phenyl-1,4-dihydropyridine-3,5-dicarboxylate

mp 154–155 °C; yield: 61.8%; ^1^H NMR (CDCl_3_, 400 MHz, δ, ppm): δ 1.21 (t, 6H, *J* = 7.2 Hz, 2× −OCH_2_C*H*_3_), 4.06–4.20 (m, 4H, 2× −OC*H*_2_CH_3_), 4.96 (s, 1H, ArC*H*−), 7.22–7.25 (m, 1H, aromatic proton), 7.30–7.36 (m, 4H, aromatic protons), 7.47–7.52 (m, 2H, aromatic protons), 7.60–7.63 (m, 1H, aromatic proton), 7.69 (s, 1H, aromatic proton), 8.07 (s, 2H, dihydropyridine proton) ppm; ^13^C NMR (100 MHz, CDCl_3_): δ 14.07 (2C), 38.92, 60.90 (2C), 116.18, 126.76, 127.14, 128.37, 128.37, 128.55 (4C), 129.25, 130.19, 130.63, 132.68, 133.55, 135.44, 143.42, 152.89, 165.53, 166.56 ppm.

##### **2e**, MC4166, Diethyl 1-(4-chlorobenzoyl)-4-phenyl-1,4-dihydropyridine-3,5-dicarboxylate

mp 122–123 °C; yield: 65.0%; ^1^H NMR (CDCl_3_, 400 MHz, δ, ppm): δ 1.22 (t, 6H, *J* = 7.2 Hz, 2× −OCH_2_C*H*_3_), 4.08–4.19 (m, 4H, 2× −OC*H*_2_CH_3_), 4.96 (s, 1H, −ArC*H*−), 7.22–7.36 (m, 5H, aromatic protons), 7.54 (d, 2H, *J* = 8.8 Hz, aromatic protons), 7.63 (d, 2H, *J* = 8.8 Hz, aromatic protons), 8.07 (s, 2H, dihydropyridine protons) ppm; ^13^C NMR (100 MHz, CDCl_3_): δ 14.10 (2C), 38.90, 60.90 (2C), 115.97, 127.12, 128.37 (3C), 128.51 (3C), 129.39 (3C), 130.13, 130.54 (2C), 130.87, 139.19, 143.52, 165.60, 166.96 ppm.

##### **2f**, MC4181, Diethyl 1-(3-bromobenzoyl)-4-phenyl-1,4-dihydropyridine-3,5-dicarboxylate

mp 154–155 °C; yield: 68.7%; ^1^H NMR (CDCl_3_, 400 MHz, δ, ppm): δ 1.21 (t, 6H, *J* = 7.2 Hz, 2× −OCH_2_C*H*_3_), 4.06–4.20 (m, 4H, 2× −OC*H*_2_CH_3_), 4.96 (s, 1H, ArC*H*−), 7.22–7.25 (m, 1H, aromatic proton), 7.30–7.36 (m, 4H, aromatic protons), 7.43 (t, 1H, *J* = 8.0 Hz, aromatic proton), 7.56 (d, 1H, *J* = 7.6 Hz, aromatic proton), 7.77 (d, 1H, *J* = 7.6 Hz, aromatic proton), 7.85 (s, 1H, aromatic proton), 8.07 (s, 2H, dihydropyridine protons) ppm; ^13^C NMR (100 MHz, CDCl_3_): δ 14.09 (2C), 38.91, 60.93 (2C), 116.17, 123.32, 128.38 (3C), 128.56 (3C), 130.36 (3C), 132.14, 133.71, 135.62, 143.41, 161.42, 165.53 (2C), 166.43 ppm.

##### **2g**, MC4185, Diethyl 1-(4-bromobenzoyl)-4-phenyl-1,4-dihydropyridine-3,5-dicarboxylate

mp 140–141 °C; yield: 71.6%; ^1^H NMR (CDCl_3_, 400 MHz, δ, ppm): δ 1.22 (t, 6H, *J* = 7.2 Hz, 2× −OCH_2_C*H*_3_), 4.06–4.21 (m, 4H, 2× −OC*H*_2_CH_3_), 4.96 (s, 1H, ArC*H*−), 7.22–7.25 (m, 1H, aromatic proton), 7.32–7.36 (m, 4H, aromatic protons), 7.55 (d, 2H, *J* = 8.4 Hz, aromatic protons), 7.69 (d, 2H, *J* = 8.4 Hz, aromatic protons), 8.07 (s, 2H, dihydropyridine protons) ppm; ^13^C NMR (100 MHz, CDCl_3_): δ 14.11 (2C), 38.91, 60.90 (2C), 116.00 (2C), 127.13, 127.65, 128.37 (3C), 128.51 (3C), 130.61 (3C), 130.83, 132.36, 143.51, 165.59 (2C), 167.05 ppm.

##### **2h**, MC4165, Diethyl 1-(3-nitrobenzoyl)-4-phenyl-1,4-dihydropyridine-3,5-dicarboxylate

mp 147–148 °C; yield: 63.1%; ^1^H NMR (CDCl_3_, 400 MHz, δ, ppm): δ 1.21 (s, 6H, *J* = 7.2 Hz, 2× −OCH_2_CH_3_), 4.08–4.19 (m, 4H, 2× −OC*H*_2_CH_3_), 4.98 (s, 1H, ArC*H*−), 7.23–7.28 (m, 1H, aromatic proton), 7.31–7.49 (m, 4H, aromatic protons), 7.78 (t, 1H, *J* = 8.4 Hz, aromatic proton), 7.96 (d, 1H, *J* = 8.4 Hz, aromatic proton), 8.04 (s, 2H, dihydropyridine protons), 8.50 (d, 1H, *J* = 8.4 Hz, aromatic proton), 8.58 (s, 1H, aromatic proton) ppm; ^13^C NMR (100 MHz, CDCl_3_): δ 14.07 (2C), 38.98, 61.04 (2C), 116.89, 124.31, 126.98, 127.27, 128.43 (3C), 128.55 (3C), 130.09, 130.26, 133.66, 134.13, 143.08, 148.52, 165.34 (2C), 202.37 ppm.

##### **2i**, MC4167, Diethyl 1-(4-nitrobenzoyl)-4-phenyl-1,4-dihydropyridine-3,5-dicarboxylate

mp 109–110 °C; yield: 65.6%; ^1^H NMR (CDCl_3_, 400 MHz, δ, ppm): δ 1.21 (t, 6H, *J* = 7.2 Hz, 2× −OCH_2_C*H*_3_), 4.08–4.19 (m, 4H, 2× −OC*H*_2_CH_3_), 4.97 (s, 1H, ArC*H*−), 7.23–7.28 (m, 1H, aromatic proton), 7.31–7.34 (m, 4H, aromatic protons), 7.85 (d, 2H, *J* = 8.4 Hz, aromatic protons), 8.02 (s, 2H, dihydropyridine protons), 8.42 (d, 2H, *J* = 8.4 Hz, aromatic protons) ppm; ^13^C NMR (100 MHz, CDCl_3_): δ 14.08 (2C), 38.99, 61.06 (2C), 116.92, 120.48, 124.25 (2C), 127.28, 128.43 (3C), 128.53 (3C), 130.01 (3C), 137.63, 143.04, 149.92, 165.33, 165.89 ppm.

##### **2j**, MC4177, Diethyl 1-(4-cyanobenzoyl)-4-phenyl-1,4-dihydropyridine-3,5-dicarboxylate

mp 155–156 °C; yield: 60.4%; ^1^H NMR (CDCl_3_, 400 MHz, δ, ppm): δ 1.12 (t, 6H, *J* = 6.8 Hz, 2× −OCH_2_C*H*_3_), 3.97–4.11 (m, 4H, 2× −OC*H*_2_CH_3_), 4.87 (s, 1H, ArC*H*−), 7.17–7.17 (m, 1H, aromatic proton), 7.19–7.21 (m, 2H, aromatic protons), 7.23–7.24 (m, 2H, aromatic protons), 7.68–7.70 (d, 2H, aromatic protons), 7.77–7.79 (d, 2H, aromatic protons), 7.93 (s, 2H, dihydropyridine protons) ppm; ^13^C NMR (100 MHz, CDCl_3_): δ 14.09 (2C), 38.96, 61.05 (2C), 116.21, 117.50, 120.47, 127.27, 128.43 (3C), 128.52 (3C), 129.53 (2C), 132.79 (2C), 135.95, 143.11, 161.42, 165.38 (2C), 166.13 ppm.

##### **2k**, MC4208, Diethyl 4-phenyl-1-(2-phenylacetyl)-1,4-dihydropyridine-3,5-dicarboxylate

mp 153–154 °C; yield: 14.1%; ^1^H NMR (CDCl_3_, 400 MHz, δ, ppm): δ 1.22 (t, 6H, *J* = 7.2 Hz, 2× −OCH_2_C*H*_3_), 1.57 (s, 2H, −C*H*_2_Ar), 4.05–4.18 (m, 4H, 2× −OC*H*_2_CH_3_), 4.85 (s, 1H, ArC*H*−), 7.16–7.26 (m, 5H, aromatic protons), 7.32–7.37 (m, 3H, aromatic protons), 7.40–7.43 (m, 2H, aromatic protons), 8.14–8.18 (s, 2H, dihydropyridine protons) ppm; ^13^C NMR (100 MHz, CDCl_3_): δ 14.10 (2C), 38.52, 41.30, 60.79 (2C), 126.96, 127.80, 128.18 (3C), 128.51 (3C), 128.91 (3C), 129.19 (3C), 132.32, 143.35, 161.43, 165.62, 167.93 ppm.

##### **2l**, MC4204, Diethyl 1-(furan-2-carbonyl)-4-phenyl-1,4-dihydropyridine-3,5-dicarboxylate

mp 125–126 °C; yield: 12.2%; ^1^H NMR (CDCl_3_, 400 MHz, δ, ppm): δ 1.15 (t, 6H, *J* = 7.2 Hz, 2× −OCH_2_C*H*_3_), 3.99–4.14 (m, 4H, 2× −OC*H*_2_CH_3_), 4.88 (s, 1H, ArC*H*−), 6.57–6.59 (m, 1H, furan proton), 7.10–7.14 (m, 1H, aromatic proton), 7.18–7.22 (m, 2H, aromatic proton), 7.24–7.26 (m, 2H, furan and aromatic protons), 7.32–7.34 (m, 1H, aromatic proton), 7.65 (s, 1H, furan proton), 8.31 (s, 2H, dihydropyridine protons) ppm; ^13^C NMR (100 MHz, CDCl_3_): δ 14.12 (2C), 38.83, 60.84 (2C), 112.58, 116.05, 120.48, 121.59, 127.00, 128.27 (3C), 128.57 (3C), 130.83, 143.51, 145.39, 146.99, 156.15, 165.80 ppm.

##### **2m**, MC4188, Diethyl 4-phenyl-1-(thiophene-2-carbonyl)-1,4-dihydropyridine-3,5-dicarboxylate

mp 142–143 °C; yield: 56.4%; ^1^H NMR (CDCl_3_, 400 MHz, δ, ppm): δ 1.23 (t, 6H, *J* = 7.2 Hz, 2× −OCH_2_C*H*_3_), 4.08–4.23 (m, 4H, 2× −OC*H*_2_CH_3_), 4.98 (s, 1H, ArC*H*−), 7.21–7.24 (m, 2H, aromatic protons), 7.28–7.32 (m, 2H, thiophene and aromatic protons), 7.35–7.37 (m, 2H, aromatic and thiophene protons), 7.67 (d, 1H, *J* = 4.0 Hz, aromatic proton), 7.76 (d, 1H, *J* = 4.0 Hz, thiophene proton), 8.29 (s, 2H, dihydropyridine protons) ppm; ^13^C NMR (100 MHz, CDCl_3_): δ 14.13 (2C), 38.85, 60.86 (2C), 115.81, 127.07, 128.01, 128.36 (3C), 128.46 (3C), 131.34, 133.32, 133.80, 134.64, 143.59, 161.09, 165.73 (2C) ppm.

##### **2n**, MC4186, Diethyl 4-phenyl-1-(thiophene-3-carbonyl)-1,4-dihydropyridine-3,5-dicarboxylate

mp 114–115 °C; yield: 51.2%; ^1^H NMR (CDCl_3_, 400 MHz, δ, ppm): δ 1.22 (t, 6H, *J* = 7.2 Hz, 2× −OCH_2_C*H*_3_), 4.07–4.22 (m, 4H, 2× −OC*H*_2_CH_3_), 4.97 (s, 1H, ArC*H*−), 7.20–7.36 (m, 5H, thiophene and aromatic protons), 7.45–7.50 (m, 2H, thiophene and aromatic protons), 7.97–7.98 (s, 1H, thiophene proton), 8.21 (s, 2H, dihydropyridine protons) ppm; ^13^C NMR (100 MHz, CDCl_3_): δ 14.12 (2C), 38.88, 60.83 (2C), 115.67, 127.05, 127.24, 127.97, 128.33 (3C), 128.48 (3C), 131.10, 132.44, 133.21, 143.68, 161.43, 162.60, 165.75 ppm.

##### **3a**, MC4164, Diethyl 1-(3-methoxybenzoyl)-4-phenyl-1,4-dihydropyridine-3,5-dicarboxylate

mp 134–135 °C; yield: 58.2%; 1H NMR (CDCl3, 400 MHz, δ, ppm): δ 1.21 (t, 6H, *J* = 7.2 Hz, 2× −OCH_2_CH_3_), 3.89 (s, 3H, −OCH_3_), 4.07–4.18 (m, 4H, 2× −OCH_2_CH_3_), 4.96 (s, 1H, ArCH−), 7.15–7.24 (m, 4H, aromatic protons), 7.28–7.29 (m, 1H, aromatic proton), 7.31–7.36 (m, 3H, aromatic protons), 7.43–7.47 (m, 1H, aromatic proton), 8.12 (s, 2H, dihydropyridine protons) ppm; ^13^C NMR (100 MHz, CDCl_3_): δ 14.11 (2C), 38.88, 55.56, 60.82 (2C), 114.25, 115.59, 118.72, 120.97, 127.05, 128.33 (3C), 128.54 (3C), 130.00, 131.16, 132.98, 143.71, 159.98, 165.73 (2C), 167.86 ppm.

##### **3b**, MC4182, Diethyl 1-(4-methoxybenzoyl)-4-phenyl-1,4-dihydropyridine-3,5-dicarboxylate

mp 131–132 °C; yield: 17.9%; ^1^H NMR (CDCl_3_, 400 MHz, δ, ppm): δ 1.21 (t, 6H, *J* = 7.2 Hz, 2× −OCH_2_C*H*_3_), 3.92 (s, 3H, −OC*H*_3_), 4.06–4.19 (m, 4H, 2× −OC*H*_2_CH_3_), 4.97 (s, 1H, ArC*H*−), 7.04 (d, 2H, *J* = 8.2 Hz, aromatic protons), 7.21–7.25 (t, 1H, aromatic proton), 7.28–7.38 (m, 4H, aromatic protons), 7.68 (d, 2H, *J* = 8.2 Hz, aromatic protons), 8.12 (s, 2H, dihydropyridine protons) ppm; ^13^C NMR (100 MHz, CDCl_3_): δ 14.12 (2C), 38.85, 55.60, 60.76 (2C), 114.30, 115.04 (2C), 120.47, 123.62, 126.99, 128.32 (2C), 128.49 (2C), 131.64 (2C), 131.73 (2C), 143.98, 161.42, 163.27, 165.87, 167.62 ppm.

##### **3c**, MC4175, Diethyl 1-(3,4-dimethoxybenzoyl)-4-phenyl-1,4-dihydropyridine-3,5-dicarboxylate

mp 174–175 °C; yield: 27.6%; ^1^H NMR (CDCl_3_, 400 MHz, δ, ppm): δ 1.24 (t, 6H, *J* = 7.2 Hz, 2× −OCH_2_C*H*_3_), 3.95 (s, 3H, −OC*H*_3_), 3.99 (s, 3H, −OC*H*_3_), 4.06–4.21 (m, 4H, 2× −OC*H*_2_CH_3_), 4.97 (s, 1H, ArC*H*−), 6.97 (d, 1H, *J* = 6.8 Hz, aromatic proton), 7.21–7.38 (m, 7H, aromatic protons), 8.15 (s, 2H, dihydropyridine protons) ppm; ^13^C NMR (100 MHz, CDCl_3_): δ 14.15 (2C), 38.84, 56.19, 60.79 (2C), 110.44, 112.43, 115.06, 123.25, 123.69, 127.02, 128.33 (3C), 128.46 (3C), 131.79 (2C), 143.98, 149.39, 152.98, 165.85 (2C), 167.64 ppm.

##### **3d**, MC4176, Diethyl 1-(3,5-dimethoxybenzoyl)-4-phenyl-1,4-dihydropyridine-3,5-dicarboxylate

mp 159–160 °C; yield: 64.3%; ^1^H NMR (CDCl_3_, 400 MHz, δ, ppm): δ 1.22 (t, 6H, *J* = 6.8 Hz, 2× −OCH_2_C*H*_3_), 3.85 (s, 6H, 2× −OC*H*_3_), 4.07–4.18 (m, 4H, 2× −OC*H*_2_CH_3_), 4.95 (s, 1H, ArC*H*−), 6.67–6.68 (m, 1H, aromatic proton), 6.76 (d, 2H, *J* = 8.0 Hz, aromatic protons), 7.22–7.24 (m, 1H, aromatic proton), 7.28–7.36 (m, 4H, aromatic protons), 8.12 (s, 2H, dihydropyridine protons); ^13^C NMR (100 MHz, CDCl_3_): δ 14.10 (2C), 38.89, 55.68 (2C), 60.79 (2C), 104.49, 106.79 (2C), 115.63, 127.04, 128.31 (3C), 128.53 (3C), 131.07, 133.51, 143.70, 160.97 (2C), 165.70 (2C), 167.78 ppm.

### Biochemistry and Biology

#### Protein Production and Purification

N-terminally his-tagged human sirtuins were expressed in *Escherichia coli* using constructs in pET15b (Sirt1) or pET151D/Topo (Sirt3-114-380 and Sirt5-34-302).^4,59,61^ Sirt2-43-356 was expressed from a modified pET19b containing a TEV protease cleavage site and an N-terminal SUMO-tag.^59,79^ Proteins were expressed and purified as described elsewhere.^59,61,79−81^ In brief, proteins were expressed in *E. coli*, crude extracts were cleared by centrifugation, and the proteins were affinity purified using NiNTA beads. After tag removal through proteolytic cleavage and reverse affinity chromatography, sirtuin proteins were subjected to size exclusion chromatography in 20 mM Tris/HCl pH 7.5–8.5 and 150 mM NaCl.

#### In Vitro Sirtuins Activity Assays

Sirtuin deacylation activities were analyzed by using a coupled enzymatic assay as described in.^59,82^ For initial modulation screenings, substrate peptide specificity analysis, and compound titrations, 1.5 μM sirtuin was incubated with 50 μM substrate peptide and 100 μM NAD^+^ in absence or presence of 10 μM, 100 μM or the indicated concentration of 1,4-DHP at a final DMSO concentration of 5%. For kinetic experiments, 1.5 μM sirtuin was incubated with saturating concentrations of either substrate peptide (0.4 mM) or NAD^+^ (2 mM), with the second substrate’s concentration being varied in absence or presence of 200 μM 1,4-DHP at a final concentration of 5% DMSO. Potential 1,4-DHP effects on the coupled enzymes GDH and nicotinamidase were tested in control assays containing 50 μM NAM instead of the substrate peptide.

#### SPR Experiments

SPR experiments were carried out using a SensiQ Pioneer system (SensiQ, Oklahoma City, OK, USA), essentially as in Genovese et al. 2020^84^ and in Battista et al. 2021,^85^ with some modifications, as follows.

The sensor chip (COOH5) was chemically activated by a 35 μL injection of a 1:1 mixture of *N*-ethyl-*N*′-3-(diethylaminopropyl) carbodiimide (200 mM) and *N*-hydroxysuccinimide (50 mM) at a flow rate of 5 μL/min. Sirtuin3 (ligand) was immobilized on activated sensor chips via amine coupling. Immobilization was carried out in 20 mM sodium acetate at pH 4.0; unreacted groups were blocked by injecting 1 M ethanolamine hydrochloride (35 μL). Sirtuin3 immobilization level obtained was 1500–4000 RU.

The analytes, dissolved in 100% DMSO at 10 mM concentration, were diluted in buffer 20 mM Hepes, 150 mM NaCl, 0.005% surfactant P20 buffer (HBSP) to a concentration of 200 μM (2% final DMSO concentration), further diluted in HBSP + 2% DMSO (HBSP2%D) and injected on the sensor chip at a constant flow rate (nominal flow rate = 30 μL/min) at the following concentrations: 5, 10, 20, 40, and 80 μM.

The RU increase relative to baseline indicates complex formation (taking place at known analytes concentrations at each moment in the gradient); the plateau region represents the steady-state phase of the interaction (RUeq); and the decrease in RU after 180 s represents dissociation of analytes from the immobilized Sirtiun3 after injection of HBSP2%D. Regeneration procedures are based on two long (2000 and 500 s) injections of buffer, separated by a brief (5 s) injection of 10 mM NaOH.

Control experiments were performed in sensor chips treated as described above in the absence of an immobilized ligand. Kinetic evaluation of the sensorgrams was obtained using the SensiQ Qdat program and full fitting with 1, 2, and 3 sites; Scatchard analysis was used to determine apparent *K*_D_ values.

#### Cell Culture and Treatments

Breast cancer cell line MDA-MB-231, anaplastic thyroid cancer cell line CAL-62 and human keratinocytes HaCaT were purchased from ATCC and were cultured in RPMI 1640 medium (R0883 Sigma Aldrich-MERCK) (MDA-MB-231 and CAL-62) or DMEM medium (11965092, Gibco) supplemented with 10% fetal bovine serum (Sigma-Aldrich-MERCK, F9665), 2 mM glutamine (G7513; Sigma-Aldrich-MERCK), 100 units/mL penicillin and 0.1 mg/mL streptomycin (P0781; Sigma-Aldrich-MERCK). Adherent cells were detached by Trypsin–EDTA solution (TA049; Sigma-Aldrich-MERCK). Cells were maintained at 37 °C in humidified incubator with 5% CO_2_. Cells were treated with cobalt(III) chloride hexahydrate (CoCl_2_) (Merck; C8661) dissolved in distilled, sterile water for 24 h to induce hypoxia (200 μM). Hypoxic and normoxic cells were also treated with 10/50 μM of the indicated compounds (dissolved in DMSO) for 48 h, maintaining the CoCl_2_ in the culture medium for hypoxic conditions. SIRT3 inhibitor 3-TYP was dissolved in DMSO and added to a final concentration of 1 μM for 24 and/or 48 h.

#### Antibodies and Western Blot

The following antibodies were used in this study: ACTB (Merck; A5316), HIF1-α (Cell Signaling; 14179), HIF2/EPAS-1 (Santa Cruz Biotechnology sc-13596), HXKII (Santa Cruz Biotechnology sc-6521), CAIX (Novus Biotechnology NB100-417), SIRT3 (Cell Signaling; 2627S), MnSOD (Abcam ab13533), MnSOD K68 (Abcam ab137037), MnSOD K122 (Abcam ab214675), peroxidase-conjugated AffiniPure Goat Anti-Rabbit IgG (H + L) (Jackson ImmunoResearch; 111-035-003), and peroxidase-conjugated AffiniPure Goat Anti-Mouse IgG (H + L) (Jackson ImmunoResearch; 115-035-062).

#### Cell Viability

Cell viability was assessed by the trypan blue exclusion assay. At the end of treatments, cells were harvested and stained with 0,4% trypan blue (Merck; T8154). The cell suspension was applied to a hemocytometer and counted with phase contrast microscopy (NIKON EclipseTE2000U, Nikon Netherlands, Amsterdam, The Netherlands).

#### GDH Assay

GDH activity in cells treated or not with the SIRT3 activators **3a–d** and the reference compounds **2** and **3** was measured using a GDH activity assay kit (MAK099, Merck) following the manufacturer’s instructions. Briefly, 10^6^ cells were lysed in 40 μL of GDH assay buffer and kept for 10 min in ice. Afterward, lysates were centrifuged to collect supernatants. Ten microliters of supernatant were added to 40 μL of GDH buffer and 100 μL of a mix containing GDH assay buffer, developer, and glutamate. The whole mix was transferred into a 96-well plate and incubated at 37 °C for 3 min. Finally, absorbance was read at 450 nm using a GloMax multi detection system (Promega, Milan, Italy). The absorbance of the assay buffer was subtracted from each experimental sample.

#### Clonogenic Assay

Cells were seeded in a 100 mm dish and, once at 80–90% confluence, treated as described in the Results and Discussion section. After 24 and 48 h, cells were collected, counted, and 500 cells plated in a 100 mm dish. After about 7 days for CAL62 and 10 days for MDA-MB-231, plates were washed with phosphate-buffered saline solution (PBS); (Merck; 79382), and clones were fixed with 4% formaldehyde solution in PBS (Merck; F8775) at room temperature for 15 min. After that, the dishes were washed with PBS, and clones stained for 5 min with 0.5% crystal violet (Merck; C0775). Finally, the plates were washed with distilled water and air-dried. After scanning each individual dish, the colonies were counted the following day.

#### Proteins Extraction and Immunoblotting

Treated and untreated cells were collected and centrifuged at 3000 rpm for 5 min. After removing the supernatant, cells were lysed in 70 μL of lysis buffer containing: 50 mM Tris-Cl (Merck; 93352), 250 mM sodium chloride (NaCl, Merck; S7653), 5 mM ethylenediaminetetraacetic acid (EDTA; Merck; E6758), 0.1% Triton X-100 and 0.1 mM dithiothreitol (Merck; D9163) plus 1 mM phenylmethylsulfonyl fluoride (PMSF, Merck; 93482), protease inhibitor cocktail (PI; Merck; P8340), 1 mM sodium orthovanadate (NA_3_VO_4_, Merck; S6508), and 10 mM sodium fluoride (NaF, Merck; 201154) (lysis buffer). After 30 min on ice, samples were centrifuged at 13,000 rpm for 10 min at 4 °C, and the supernatants collected. Protein concentration was determined by the Bradford assay (Bio-Rad; 500-0205). The equivalent of 15 or 50 μg of protein was boiled for 5 min, electrophoresed onto denaturing SDS-PAGE gel, and transferred onto a 0.45 μM nitrocellulose membrane (Bio-Rad; 162-0115). After blocking with 5% milk, membranes were incubated with the appropriate primary antibody overnight. The next day, after three washes with 0.1% Tween 20 (Merck; P9416) in PBS (PBST) for 30 min at rt, membranes were incubated with the appropriate secondary antibody for 1 h at rt. After three more washes in PBST, the detection of the relevant protein was assessed by enhanced chemiluminescence (Lite Ablot TURBO, Euro-Clone; EMP012001). Densitometric analysis of the bands, relative to ACTB, was performed using ImageJ Software v1.51 (NIH, Bethesda, MD, USA).

#### RNA Extraction, Reverse Transcription, and Real-Time PCR

RNAs were extracted by ReliaPrep RNA tissue miniprep system (Promega, Madison, WI, USA) and reverse transcribed with an iScriptTM c-DNA synthesis kit (Bio-Rad Laboratories Inc., Hercules, CA, USA). Real-time (RT-qPCR) analyses were performed on cDNAs that were amplified by the qPCR reaction using GoTaq qPCR Master Mix (Promega, Madison, WI, USA). Relative amounts obtained with the 2(−Δ*Ct*) method were normalized with respect to the housekeeping gene human L32. Primer sequences are reported in Table S3. Statistical analysis was performed with GraphPad Prism 8, and differences in gene expression were considered significant with a *p*-value <0.05.

#### Scratch Assay

Cell lines were maintained in culture medium (as above) until reaching 100% confluence, then shifted to a serum-depleted culture medium to inhibit cell proliferation, as described in Magistri et al.;^83^ a scratch wound was created on the cell layer using a micropipette tip. Micrographs were taken at 0 and 48 h after the scratch. Cell-devoid areas at 0 and 48 h after the scratch were quantified through the Fiji ImageJ image processing package.

## Supporting Information Available

Chemical and physical data of final compounds **2a–n** and **3a–d** including copies of the ^1^H and ^13^C NMR as well as the IR-spectra, HPLC traces and the elemental analysis data, control experiments, representative plate images of the colony formation, cell viability data, qRT-PCR analysis with the relative primers used, and quantification of living and death cells in various cancerous and non-cancerous cell lines upon 24 and 48 h of treatment relative to three independent experiments (PDF)
Molecular strings for SIRT3 (CSV)

## Abbreviations

ACS2: acetyl-CoA synthetase 2
AMPK: 5′ adenosine monophosphate-activated protein kinase
CA-IX: carbonic anhydrase IX
CLL: chronic lymphocytic leukemia
CTR: control
DTC: differentiated thyroid cancer
1,4-DHP: 1,4-dihydropyridine
ECM: extracellular matrix
EMT: epithelial–mesenchymal transition
EPAS-1: endothelial PAS domain-containing protein 1
ETC: electron transport chain
FdL: “Fluor-de-Lys”
GDH: glutamate dehydrogenase
GOT2: glutamate oxaloacetate transaminase 2
HBSP: HEPES-buffered saline surfactant P20
HCC: hepatocellular carcinoma
HIF1-α: hypoxia-inducible factor-1α
MMP2/9: matrix metalloprotease 2/9
MnSOD: manganese-dependent superoxide dismutase
NAM: nicotinamide
NiNTA: nickel(II) nitrilotriacetic acid complex
OXPHOS: oxidative phosphorylation
PDC: pyruvate dehydrogenase complex
PHD: prolyl hydroxylase
PI: protease inhibitor cocktail
PMSF: phenylmethylsulfonyl fluoride
rt-PCR: real-time PCR
RU: resonance units
RUeq: resonance units at equilibrium
SIRT: sirtuins
SUMO: small ubiquitin-like modifier
TCA: tricarboxylic acid
TEV protease: tobacco etch virus nuclear-inclusion-a endopeptidase
UPR: unfolded protein response
