# Supplementary material for: Novel 1,4-Dihydropyridines as Specific Binders and Activators of SIRT3 Impair Cell Viability and Clonogenicity and Downregulate Hypoxia-Induced Targets in Cancer Cells
Source: J Med Chem. 2023 Jul 13;66(14):9622–41. doi: 10.1021/acs.jmedchem.3c00337 (PMC10388363; doi:10.1021/acs.jmedchem.3c00337)
Supplement: Supplementary file 1 — jm3c00337_si_001.pdf [file jm3c00337_si_001.pdf]

Supporting Information

Novel 1,4-Dihydropyridines as Specific Binders and Activators of SIRT3 Impair Cell Viability and Clonogenicity, and Downregulate Hypoxia-Induced Targets in Cancer Cells

Clemens Zwergel,[a,¶] Michele Aventaggiato,[b,¶] Sabrina Garbo,[c,¶] Elisabetta Di Bello,[a] Bruno Fassari,[d] Beatrice Noce,[a] Carola Castiello,[a] Chiara Lambona,[a] Federica Barreca,[b] Dante Rotili,[a] Rossella Fioravanti,[a] Thomas Schmalz,[e] Michael Weyand,[f] Amelie Niedermeier,[f] Marco Tripodi,[c] Gianni Colotti,[g] Clemens Steegborn,[e] Cecilia Battistelli,[c,]* Marco Tafani,[b,]* Sergio Valente,[a,]* and Antonello Mai[a,h]

[a]*Department of Drug Chemistry and Technologies, Sapienza University of Rome, Piazzale Aldo Moro 5, 00185 Rome, Italy*

[b]*Department of Experimental Medicine, Sapienza University of Rome, Viale Regina Elena 324, 00161 Rome, Italy*

[c]*Department of Molecular Medicine, "Department of Excellence 2023-2027", Sapienza University of Rome, Viale Regina Elena 324, 00161 Rome, Italy.*

[d]*Dompé Farmaceutici S.p.A, Via Campo di Pile snc, 67100 L'Aquila, Italy*

[e]*University of Bayreuth, Dept. Organic Chemistry, Universitätsstr. 30, 95447 Bayreuth, Germany.*

[f]*University of Bayreuth, Dept. Biochemistry, Universitätsstr. 30, 95447 Bayreuth, Germany.*

[g]*Institute of Molecular Biology and Pathology, Italian National Research Council, Piazzale A.Moro 5, 20 Rome, Italy*

[h]*Pasteur Institute, Cenci-Bolognetti Foundation, Sapienza University of Rome, P.le A. Moro 5, 00185 Rome, Italy.*

* Corresponding authors:

(C. Battistelli) E-mail address: cecilia.battistelli@uniroma1.it

(M. Tafani) E-mail address: marco.tafani@uniroma1.it

(S. Valente) E-mail address: sergio.valente@uniroma1.it

Index:

Table S1. Chemical and Physical data of Final Compounds 2a-n and 3a-d S3

^{1}H and ^{13}C spectra of final compounds 2a-n and 3a-d S6

IR spectra and HPLC traces of final compounds 2a-n and 3a-d and high resolution mass spectra for final compounds 3a-d S24

Table S2. Elemental analysis of final compounds 2a-n and 3a-d S64

Figure S1. Control experiments for potential compound effects on the coupled enzymes of the assay system. ...S65

Figure S2. Representative plate images of the colony formation assay for CAL-62 and MDA-MB-231 cells treated with the SIRT3 activators 3a-d at 50 µM for 24 and 48 h. S66

Figure S3. Representative plate images of the colony formation assay for CAL-62 and MDA-MB-231 cells treated with increasing dose (1, 5, 10, 25 and 50 µM) of the SIRT3 activator 3d for 24 and 48 h. S67

Figure S4. Dose-dependent effects of compound 3d on cell viability, cell death and clonogenicity in CAL-62 (A, C, E) and MDA-MB-231 (B, D, F) cells upon 24 h of treatment. S68

Table S3. List of Primers used in gene expression analysis. S69

Figure S5. Dose-dependent effects of compounds 3c and 3d on cell viability (A, B) and cell death (C, D) non-tumoral HaCaT cells upon 24h (A, C) and 48h (B, D) of treatment. S69

Tables S4 (A-D). Quantification of living (A, B) and death cells (C, D) upon 24h (A, C) and 48h (B, D) of treatment relative to three independent experiments. Cell lines are indicated. S70

Table S1. Chemical and Physical data of Final Compounds 2a-n and 3a-d

Bayreuth code	Lab Code	Compound	Chemical structure	M.P. (°C)	Yield
**UBCS392**	**MC4199**	**2a**	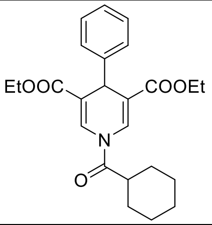	92-93°C	17.0%
**UBCS399**	**MC4158**	**2b**	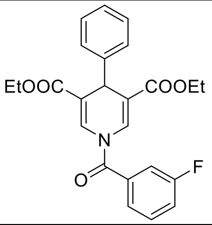	124-125°C	85,2 %
**UBCS400**	**MC4168**	**2c**	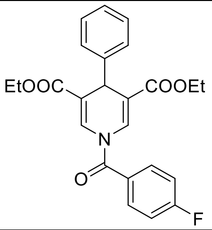	123-124°C	93,6 %
**UBCS401**	**MC4172**	**2d**	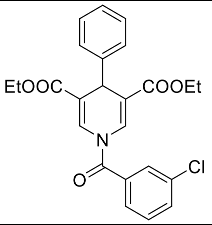	154-155°C	61.8 %
**UBCS402**	**MC4166**	**2e**	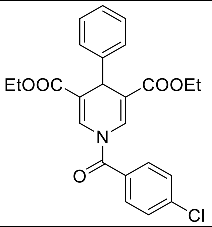	122-123°C	65.0%
**UBCS403**	**MC4181**	**2f**	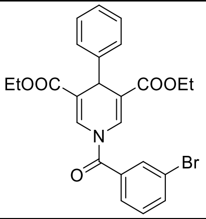	154-155°C	68,7 %
**UBCS404**	**MC4185**	**2g**	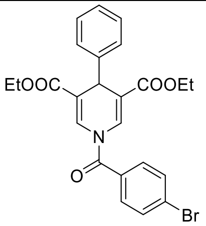	140-141°C	71,6 %
**UBCS393**	**MC4165**	**2h**	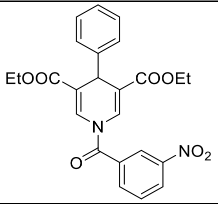	147-148°C	63,1 %

**UBCS394**	**MC4167**	**2i**	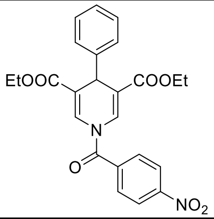	109-110°C	65,6 %
**UBCS405**	**MC4177**	**2j**	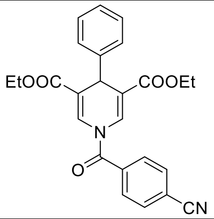	155-156°C	60,4 %
**UBCS409**	**MC4208**	**2k**	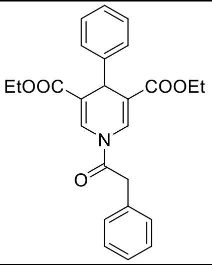	153-154°C	14,1 %
**UBCS408**	**MC4204**	**2l**	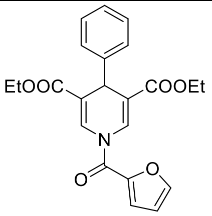	125-126°C	12,2 %
**UBCS406**	**MC4188**	**2m**	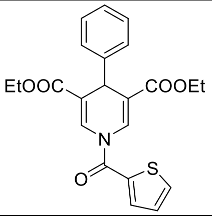	142-143°C	56,4%
**UBCS407**	**MC4186**	**2n**	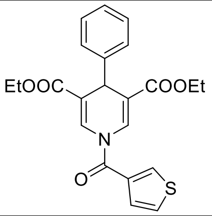	114-115°C	51,2%
**UBCS395**	**MC4164**	**3a**	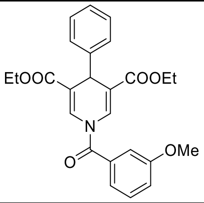	134-135°C	58,2 %
**UBCS396**	**MC4182**	**3b**	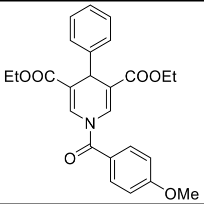	131-132°C	17,9%

**UBCS398**	**MC4175**	**3c**	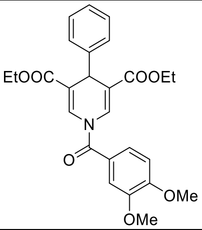	174-175°C	27,6 %
**UBCS397**	**MC4176**	**3d**	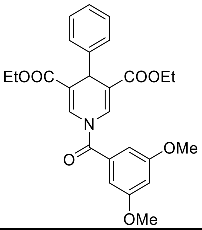	159-160°C	64, 3%

^{1}H and ^{13}C spectra of final compounds 2a-n and 3a-d

**UBCS392**	**MC4199**	**2a**	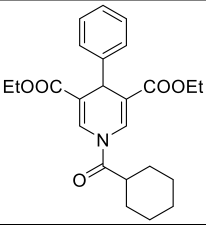

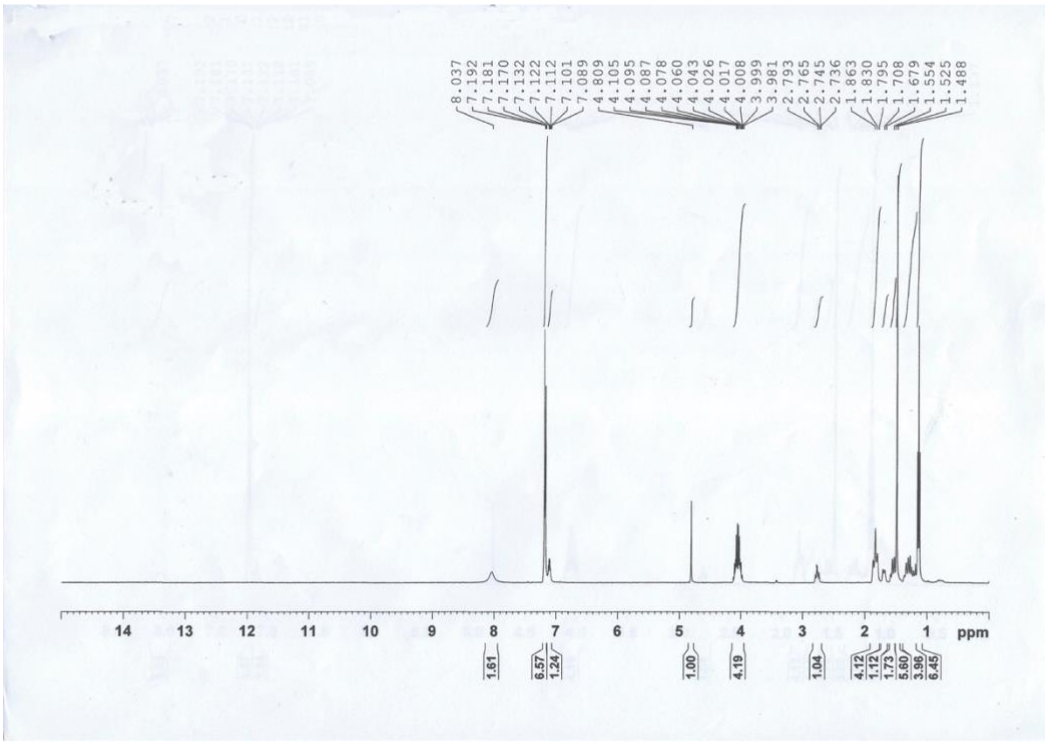

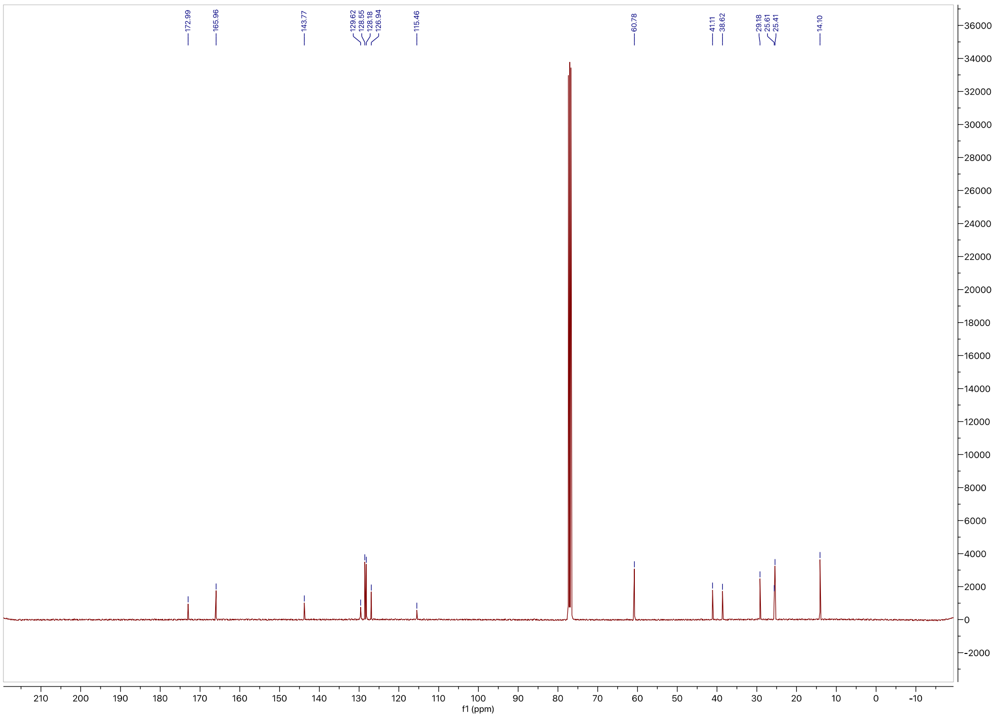

**UBCS399**	**MC4158**	**2b**	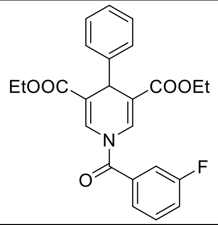

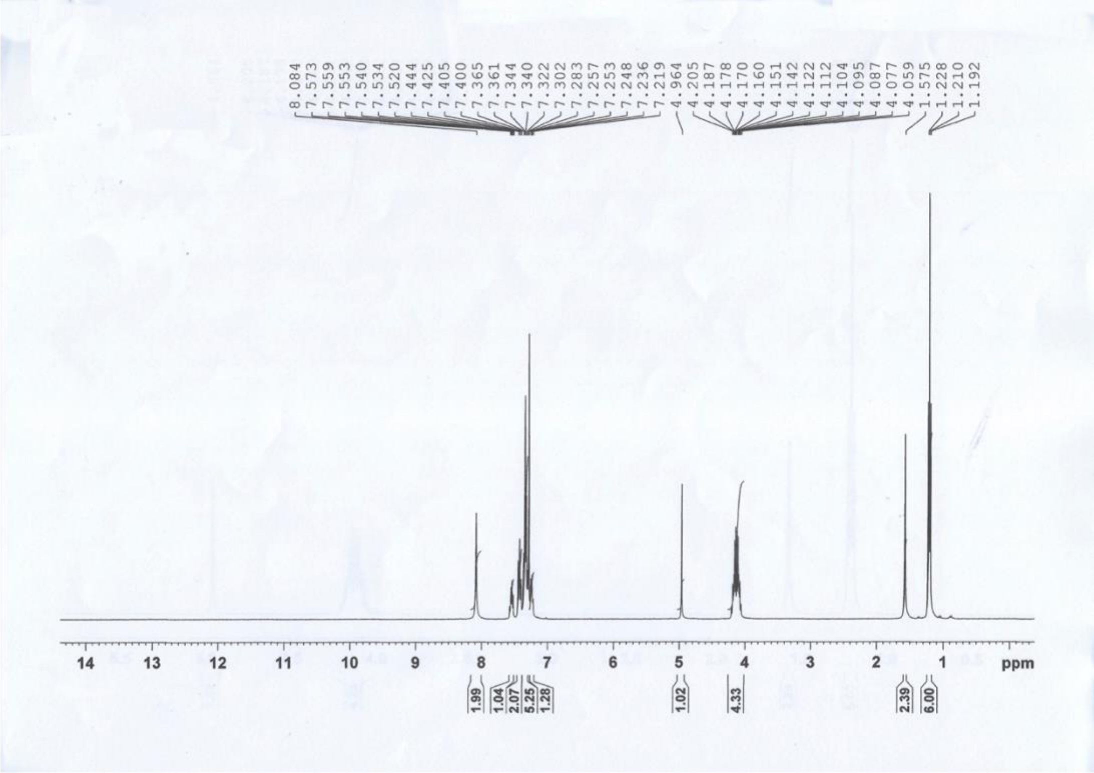

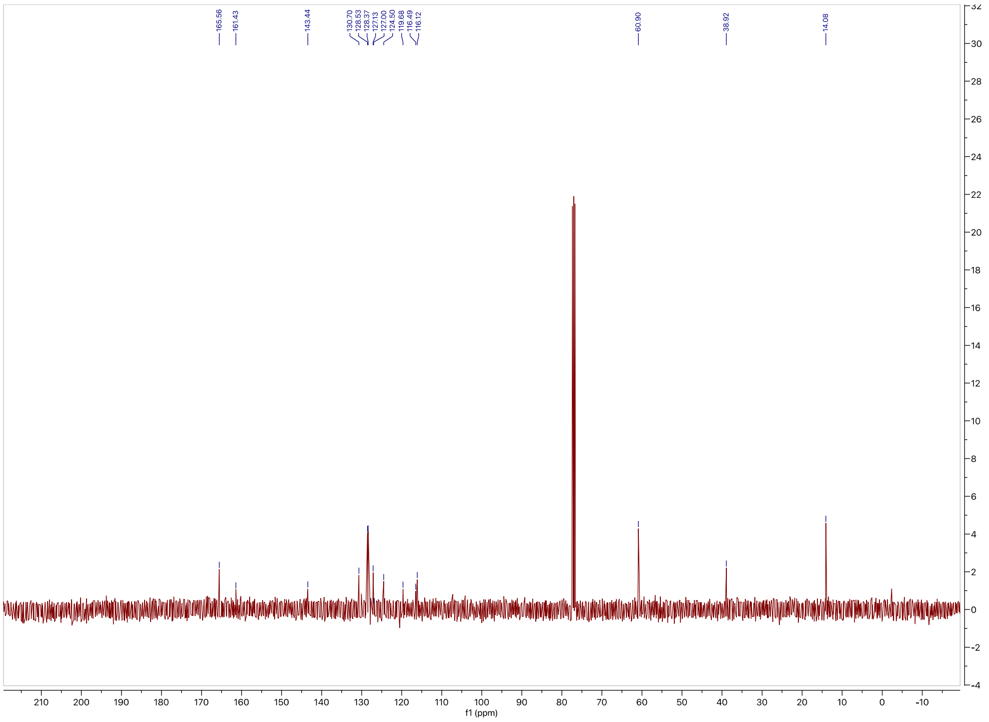

**UBCS400**	**MC4168**	**2c**	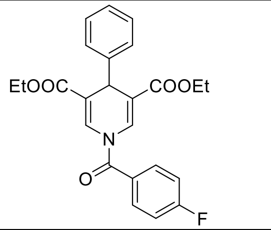

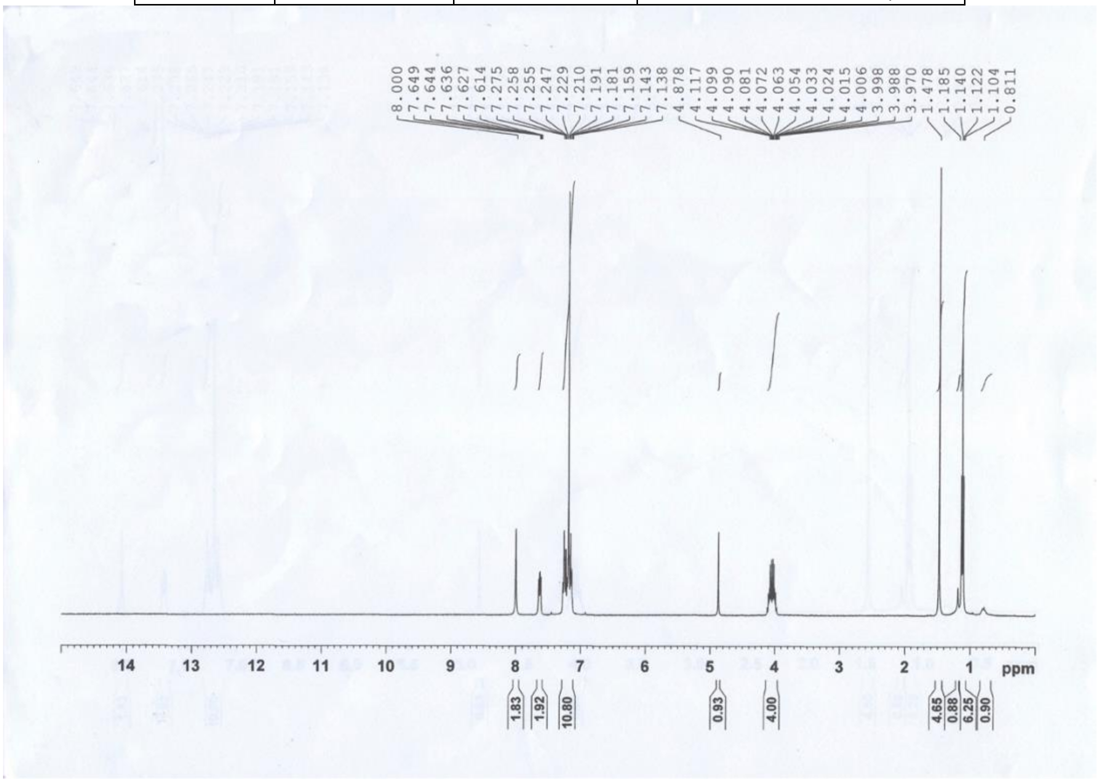

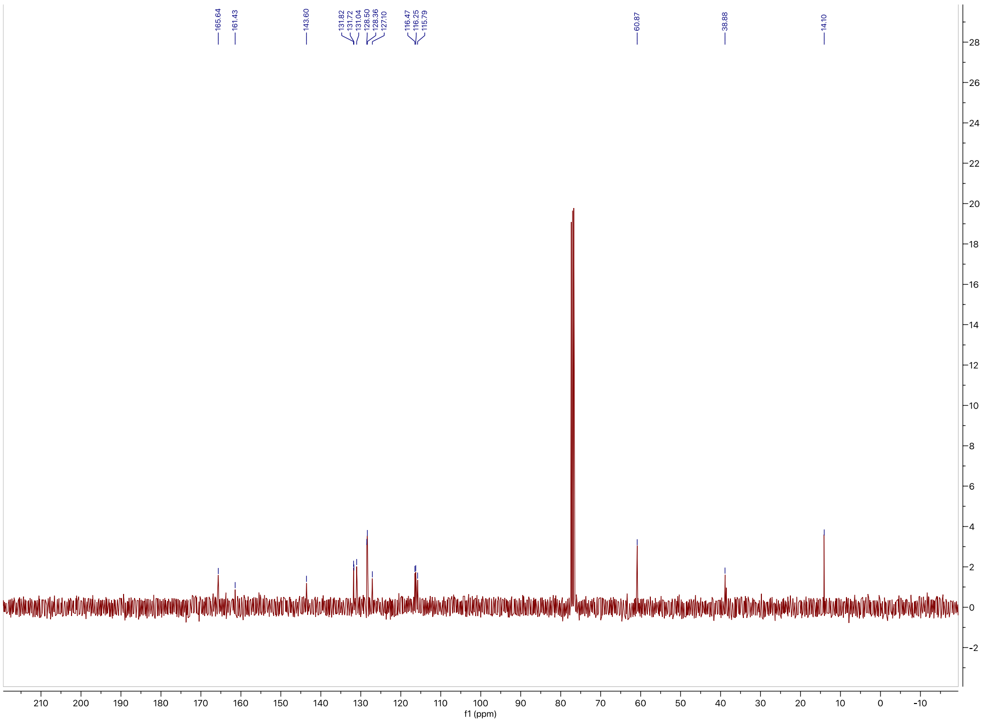

**UBCS401**	**MC4172**	**2d**	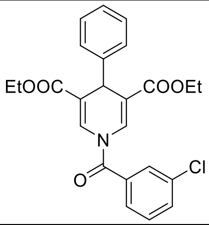

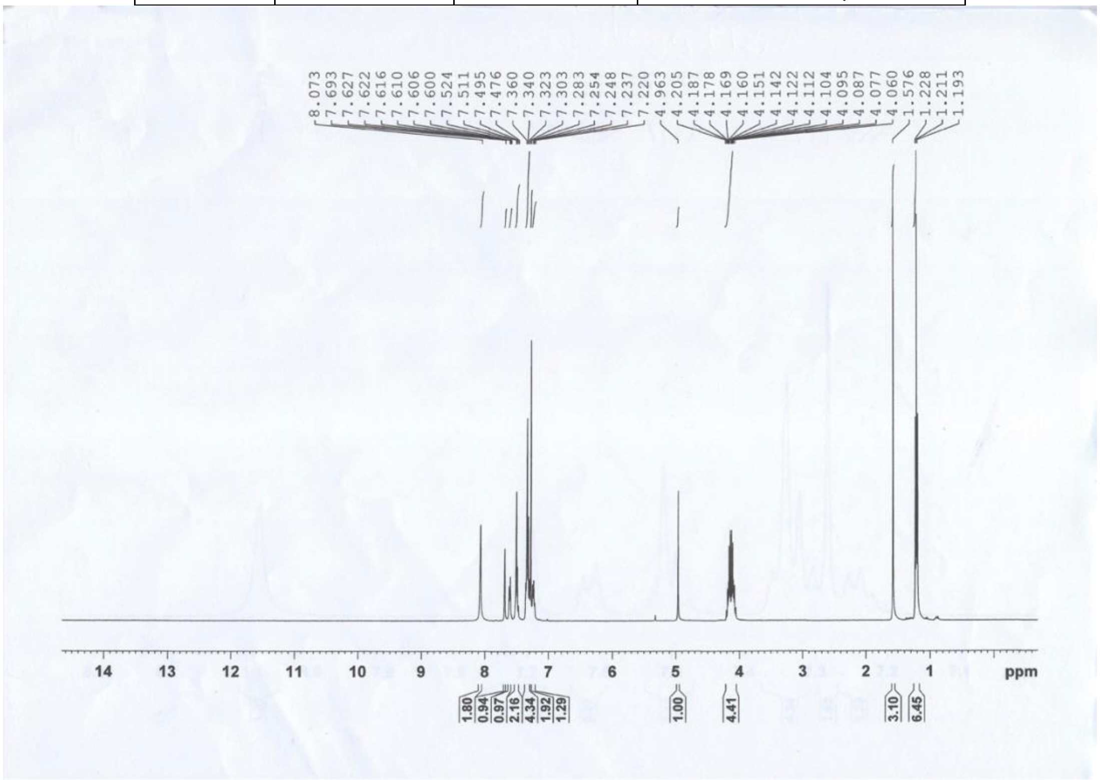

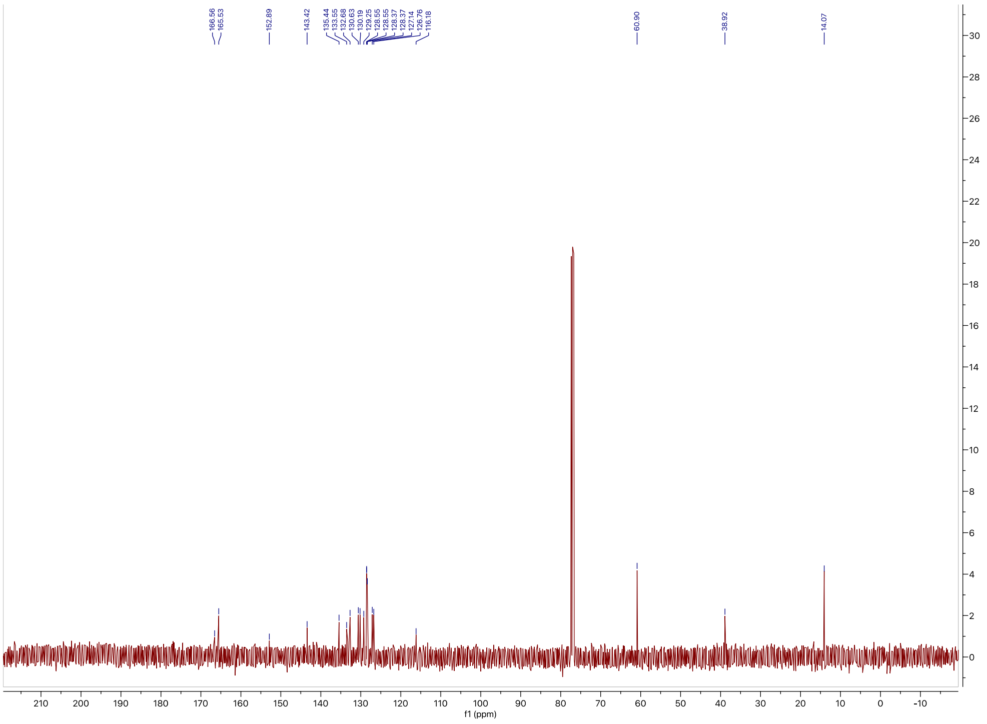

**UBCS402**	**MC4166**	**2e**	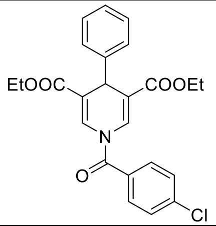

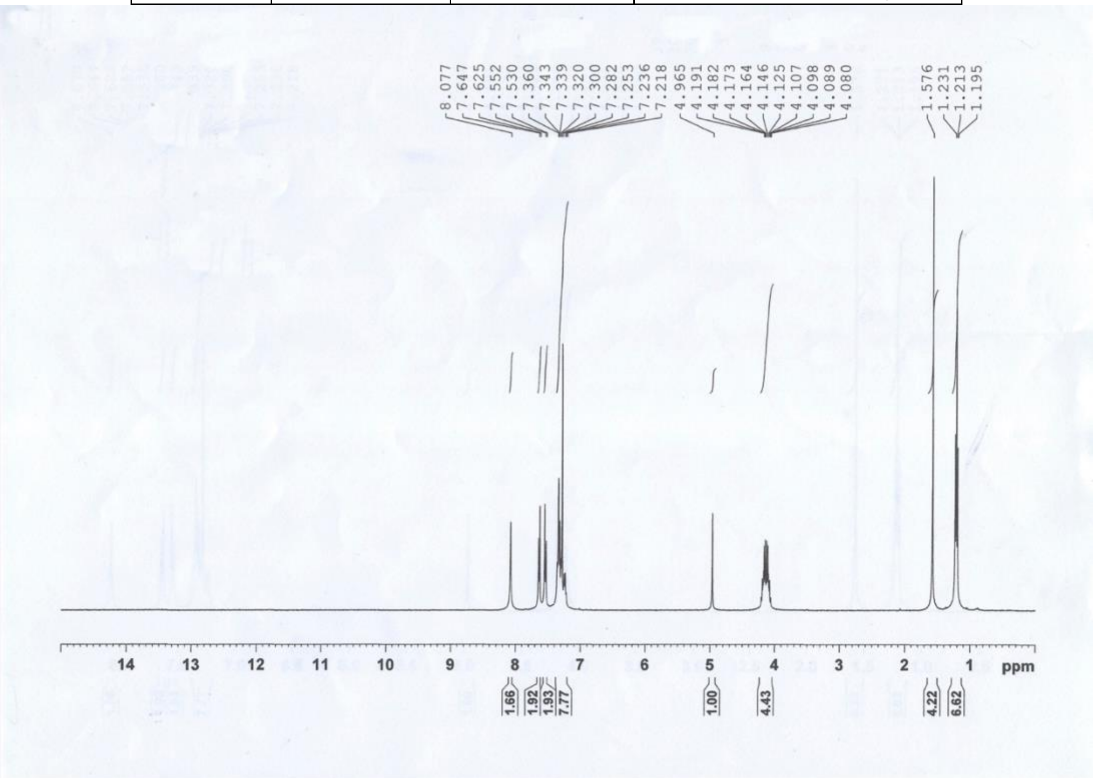

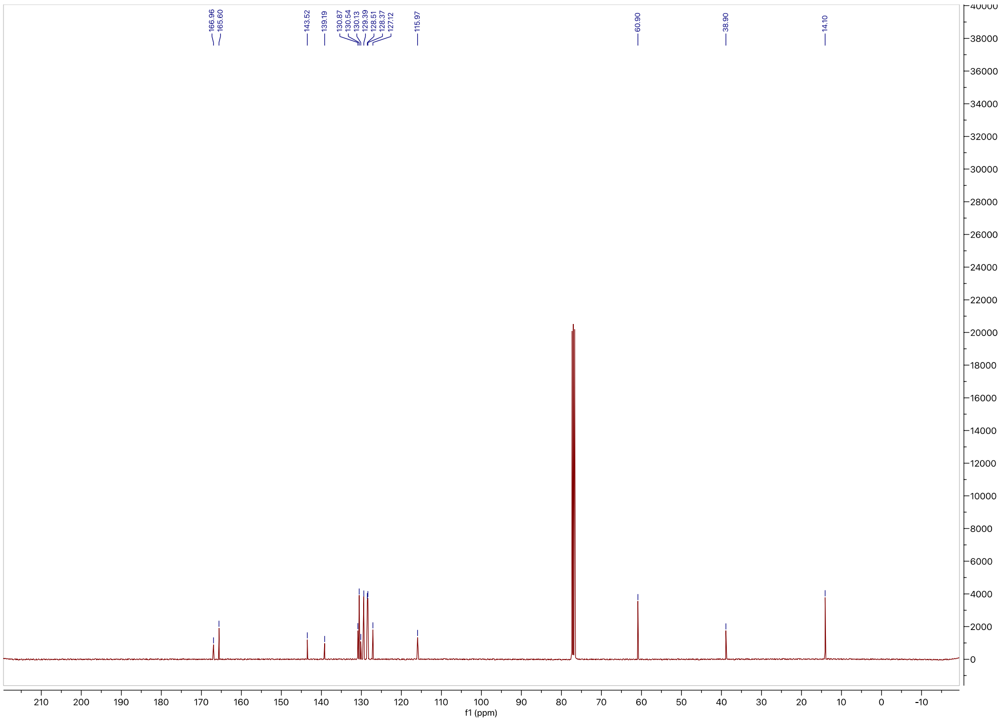

**UBCS403**	**MC4181**	**2f**	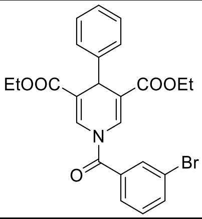

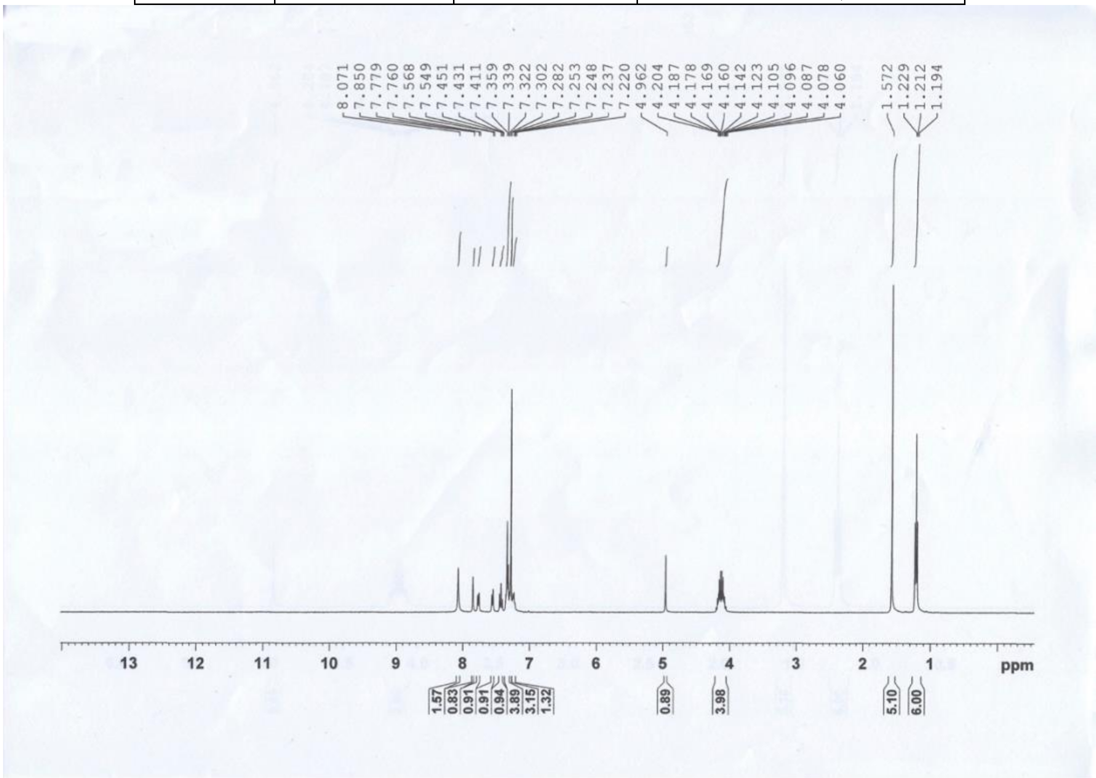

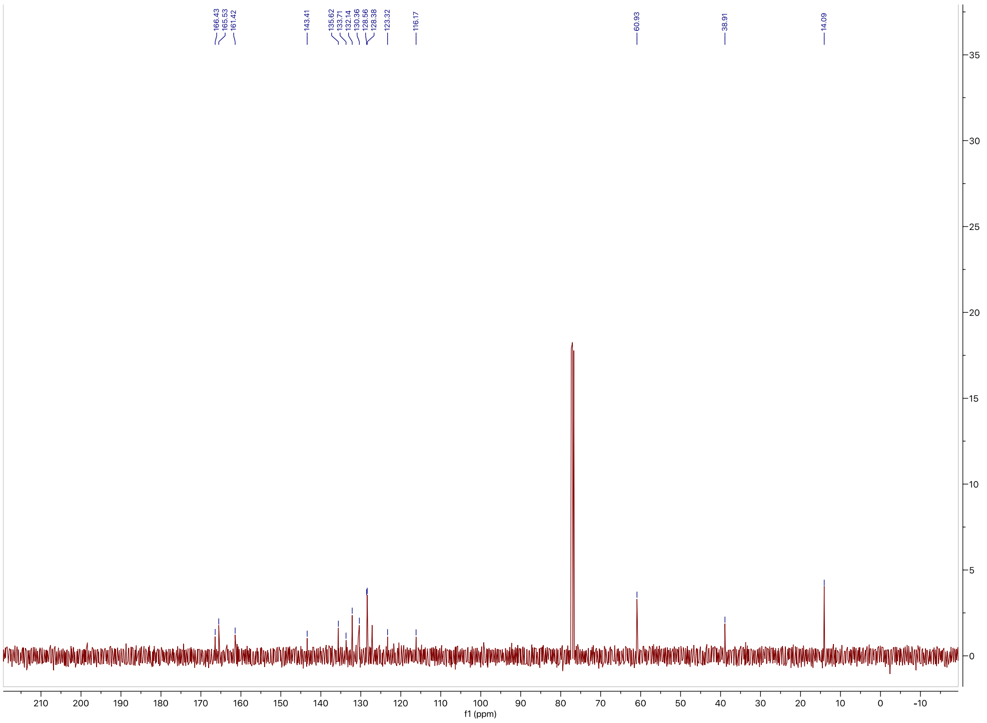

**UBCS404**	**MC4185**	**2g**	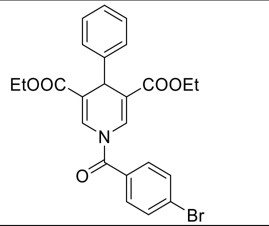

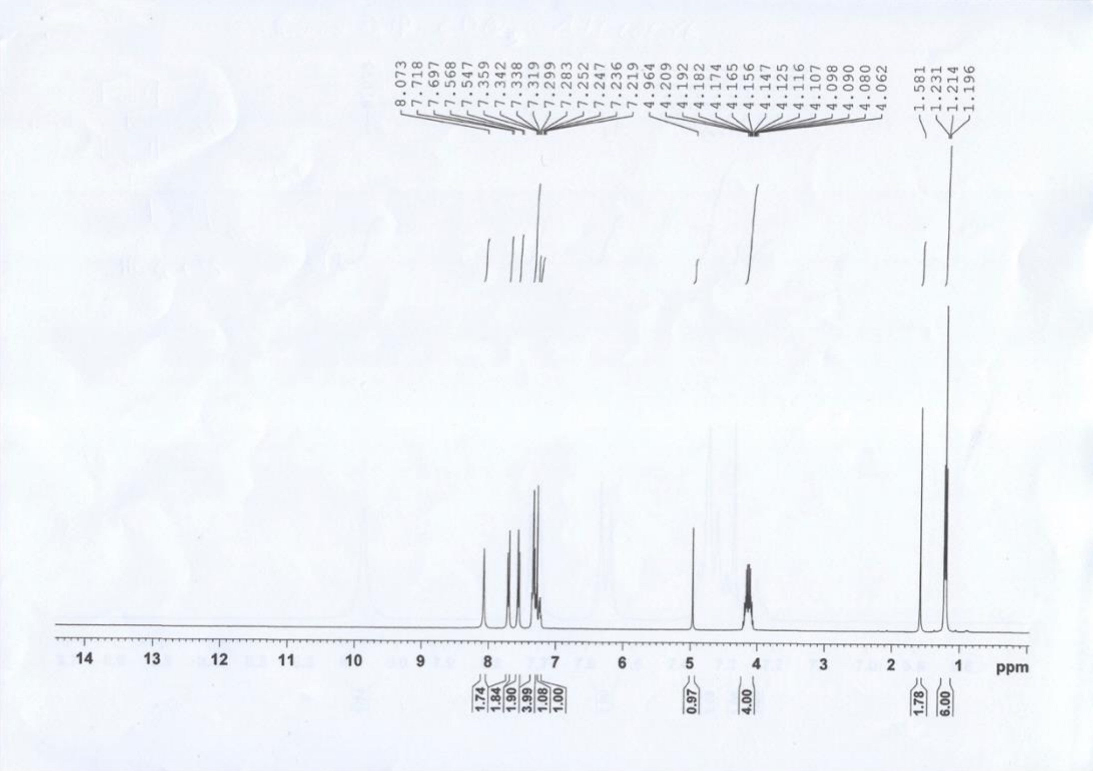

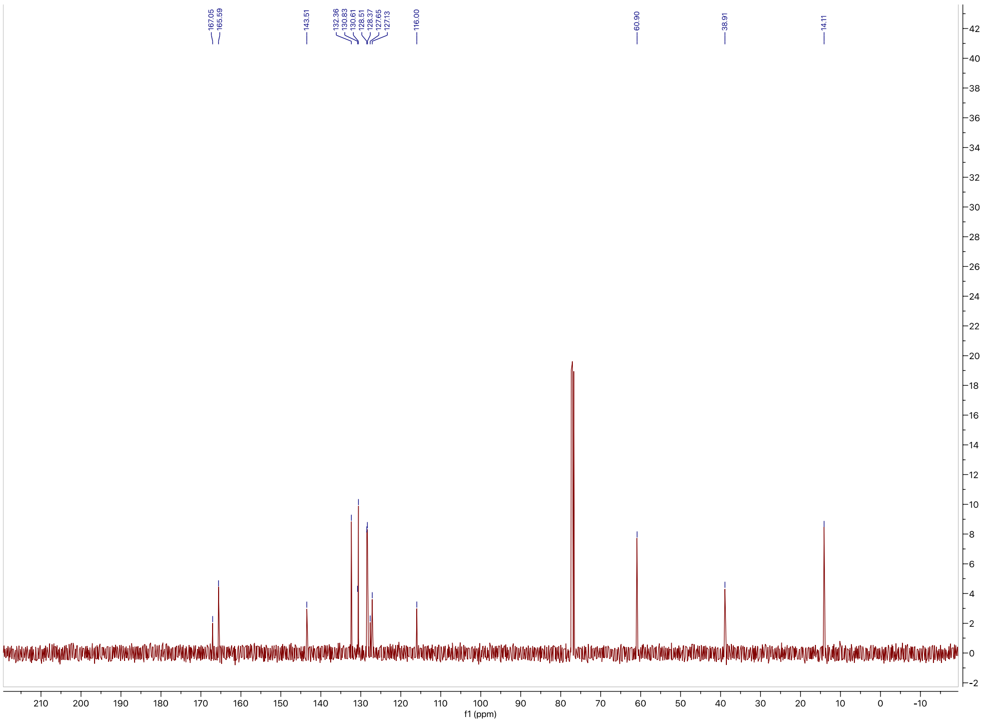

**UBCS393**	**MC4165**	**2h**	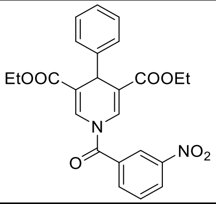

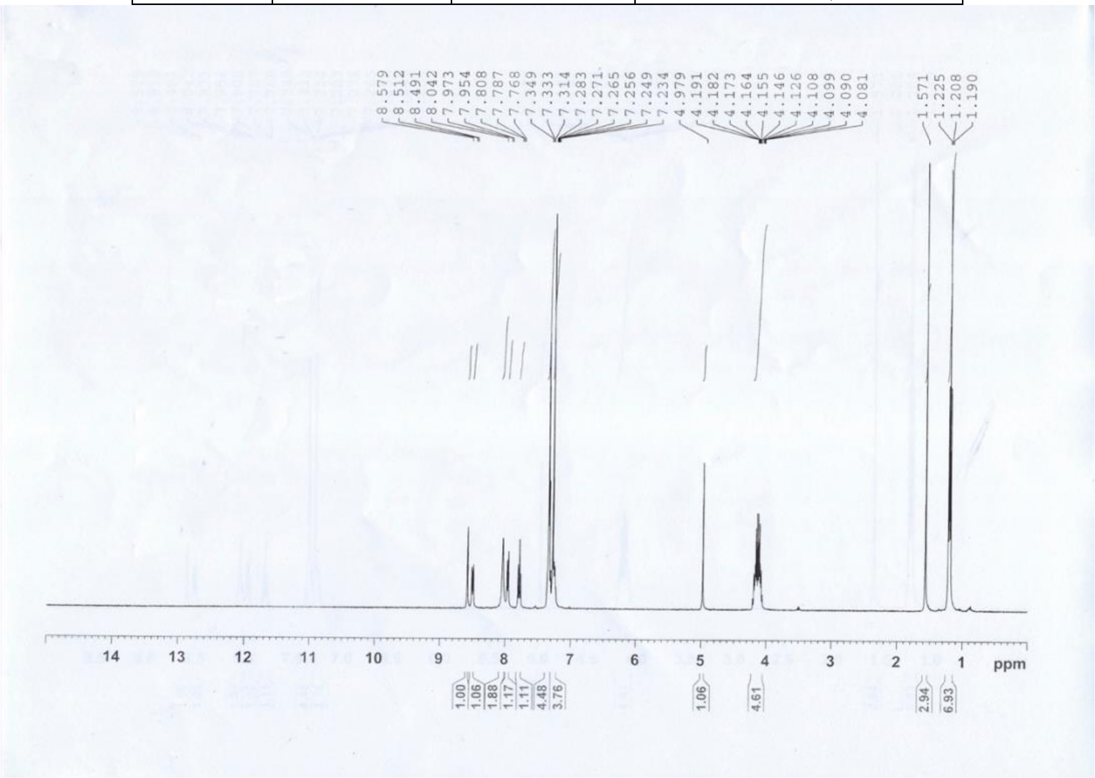

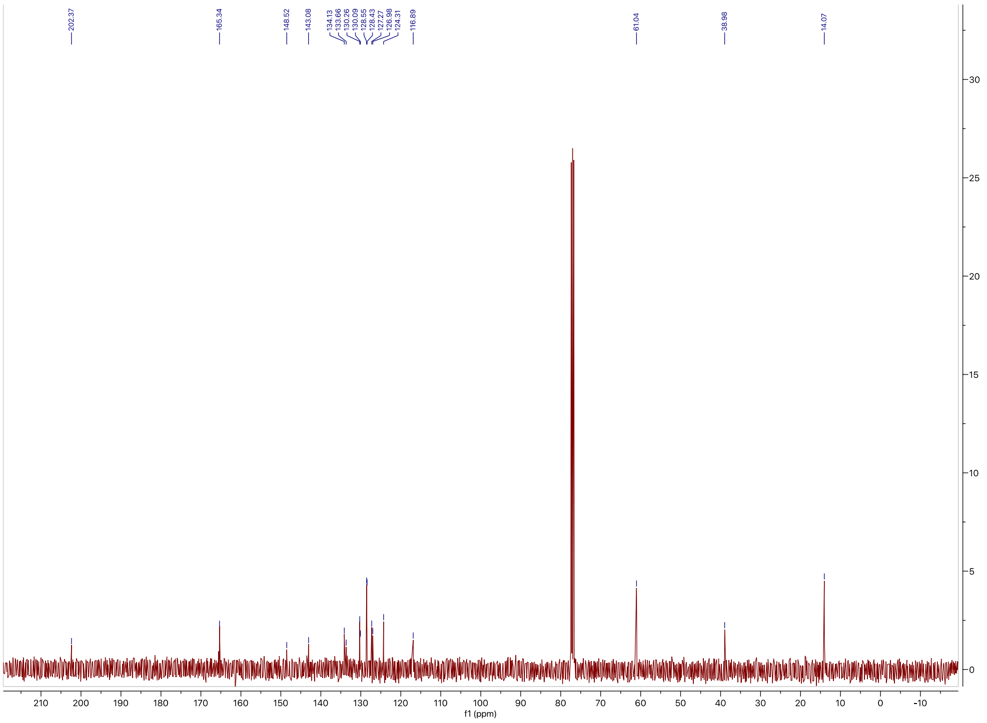

**UBCS394**	**MC4167**	**2i**	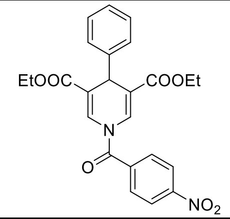

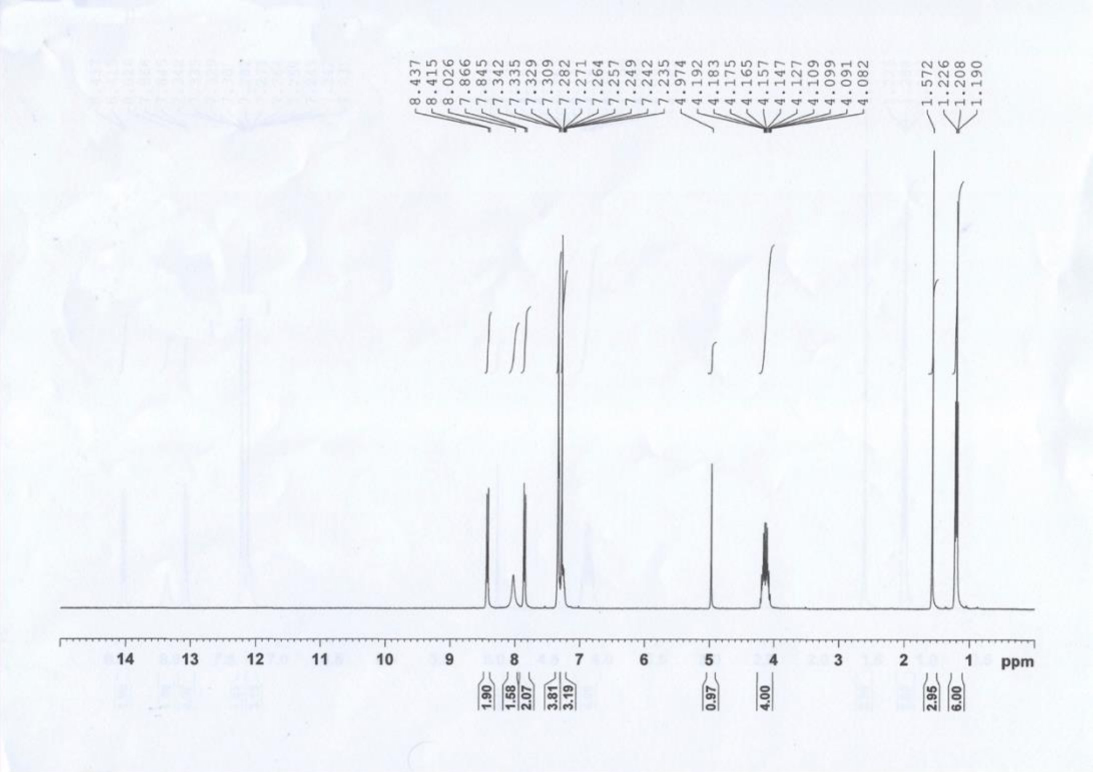

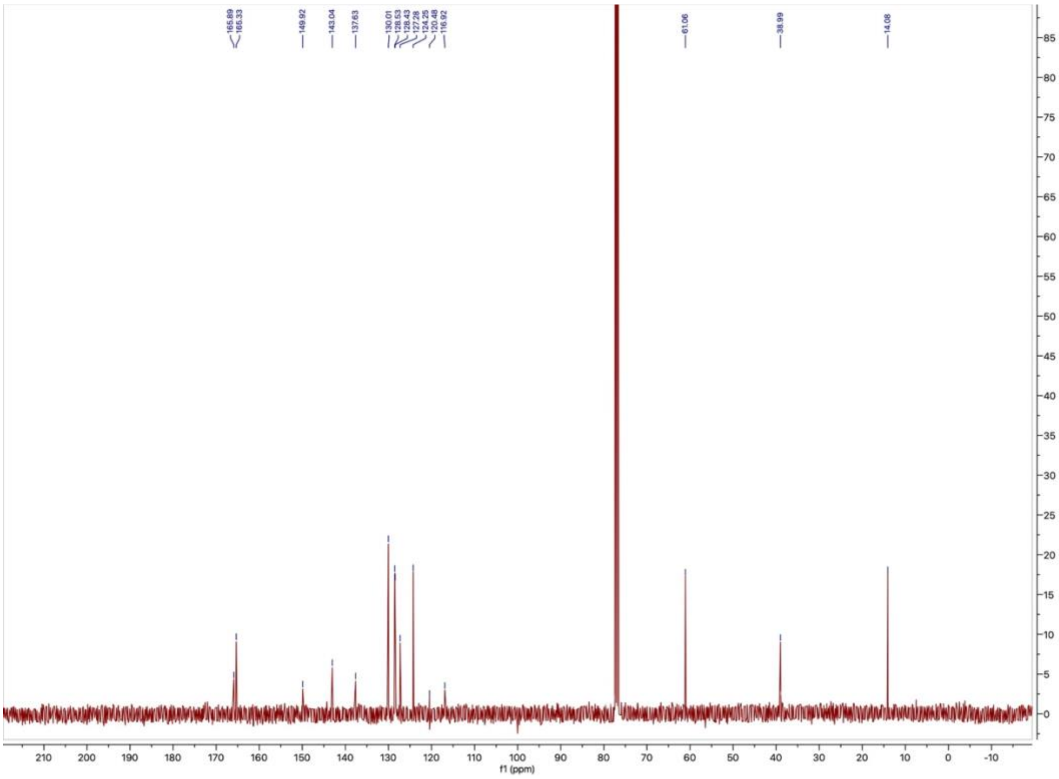

**UBCS405**	**MC4177**	**2j**	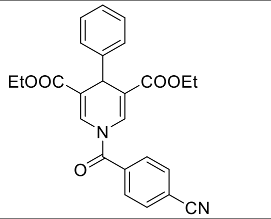

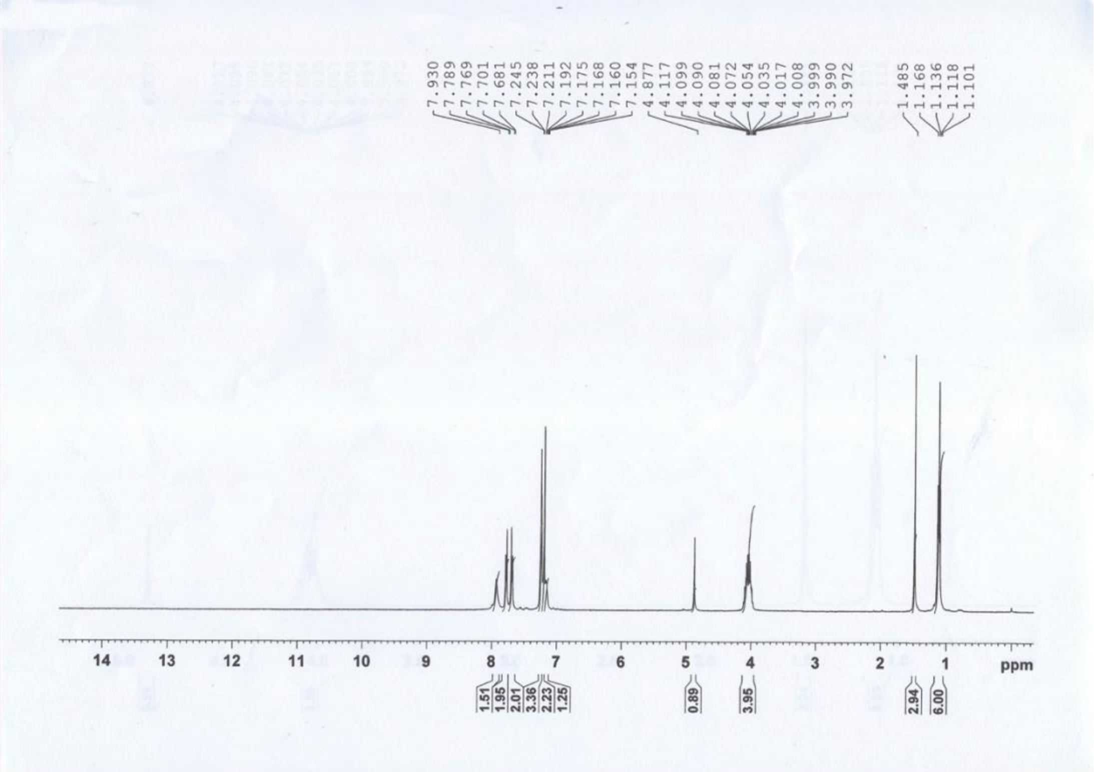

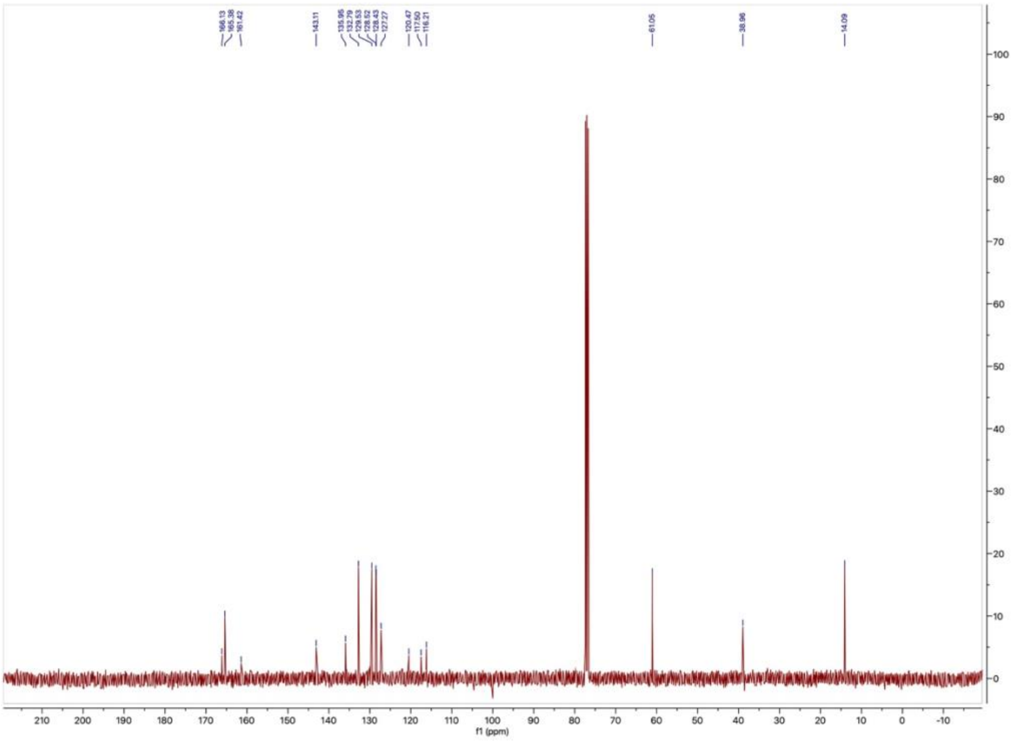

**UBCS409**	**MC4208**	**2k**	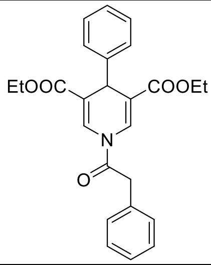

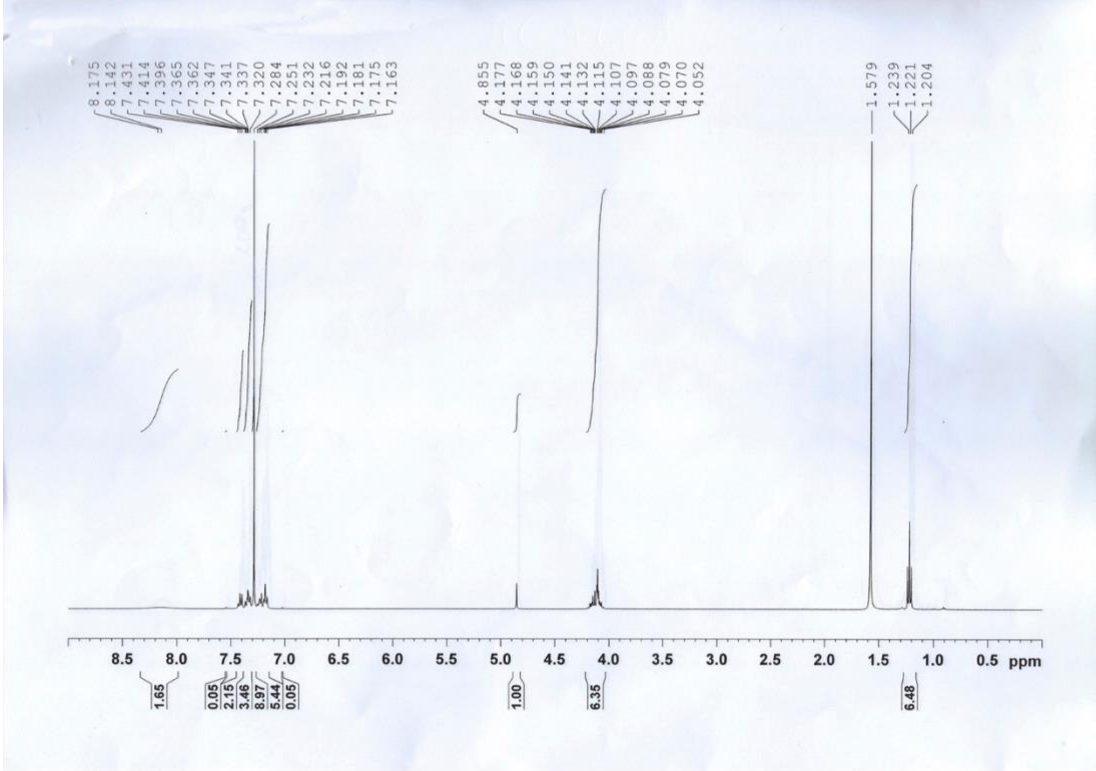

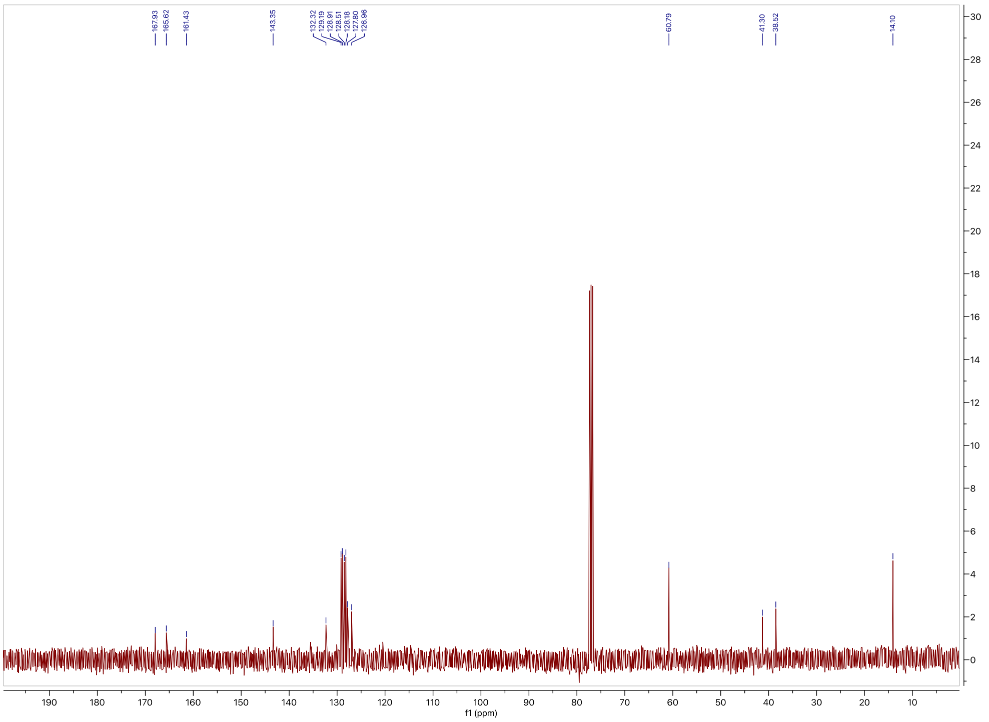

**UBCS408**	**MC4204**	**2l**	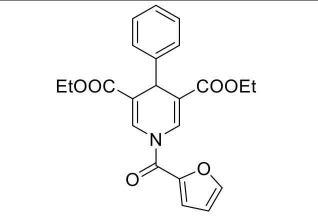

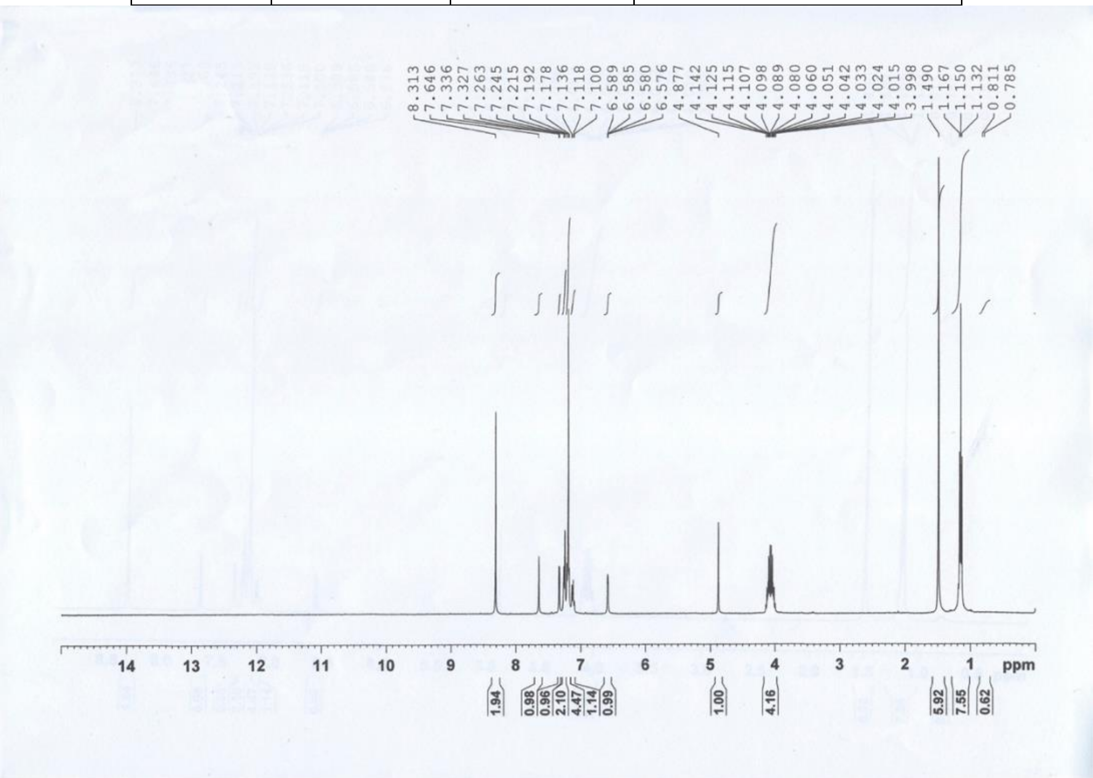

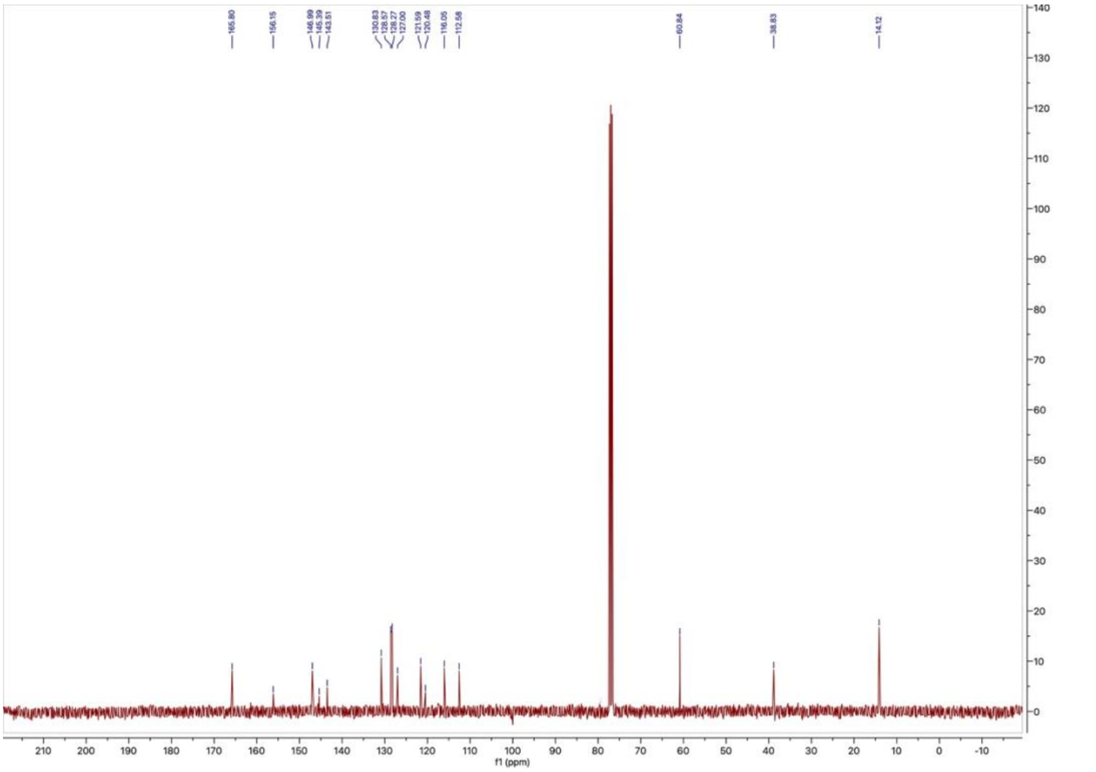

**UBCS406**	**MC4188**	**2m**	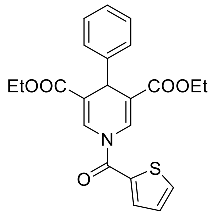

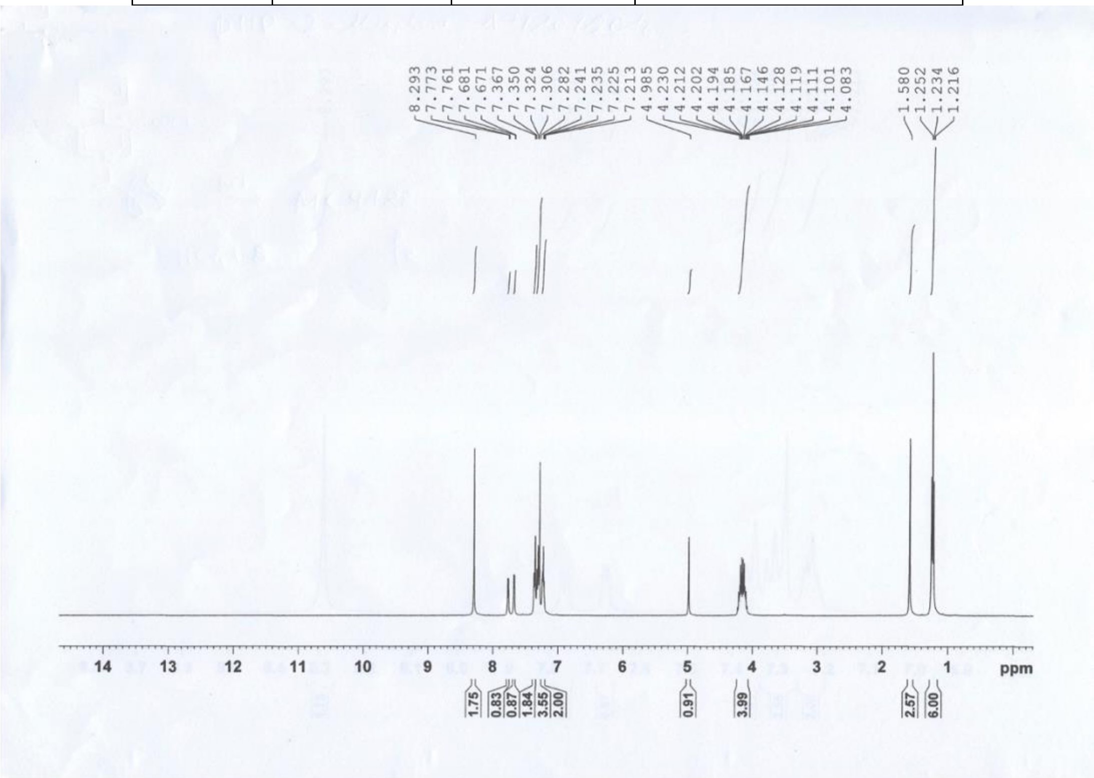

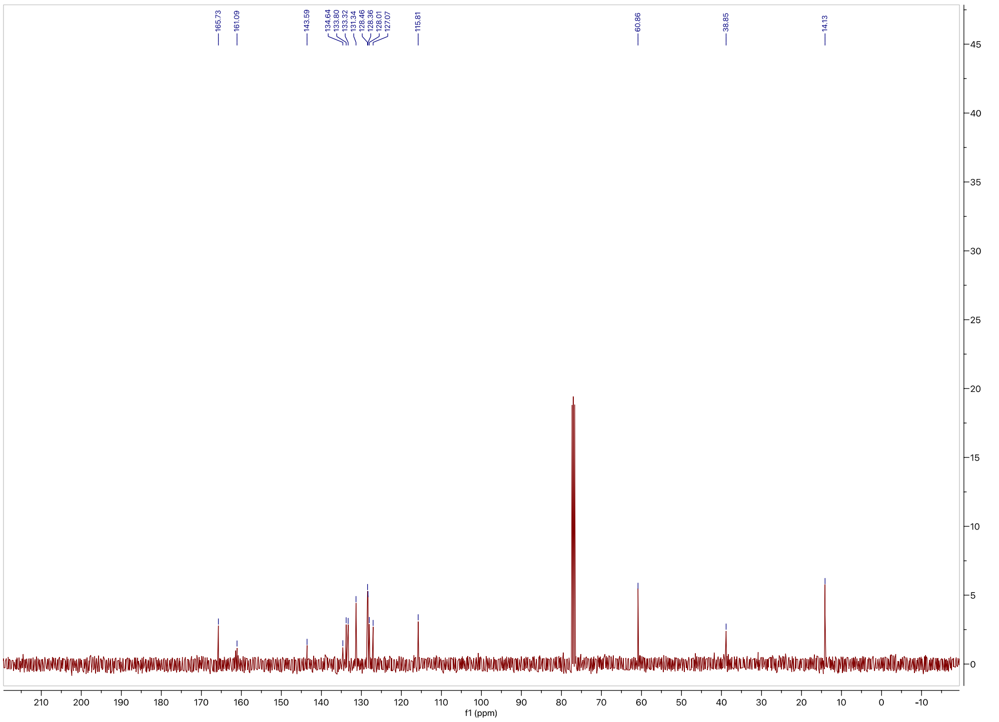

**UBCS407**	**MC4186**	**2n**	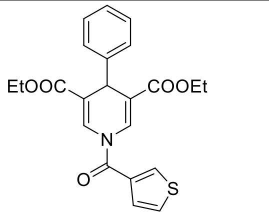

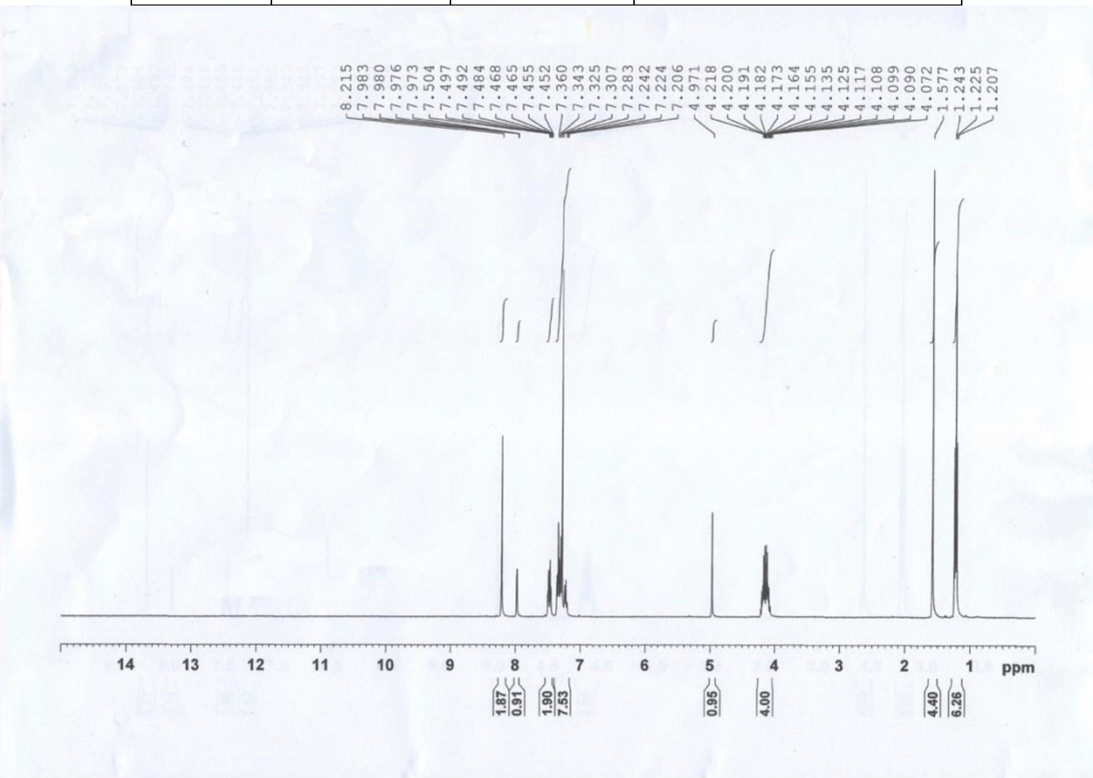

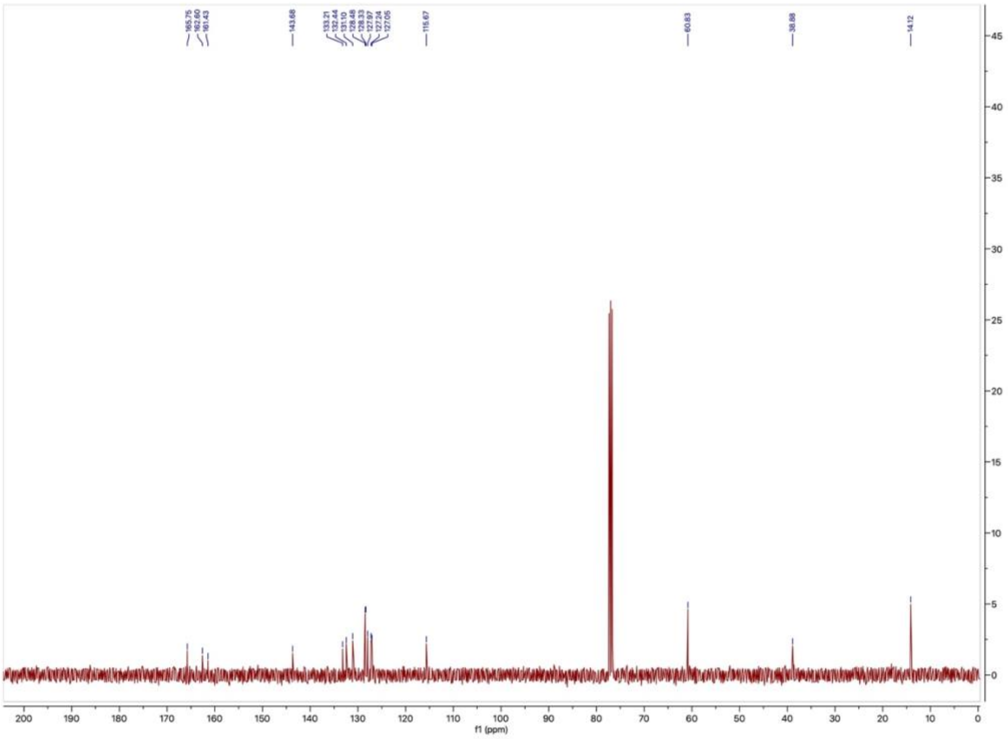

**UBCS395**	**MC4164**	**3a**	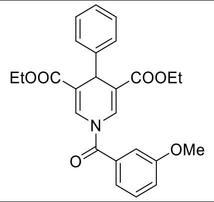

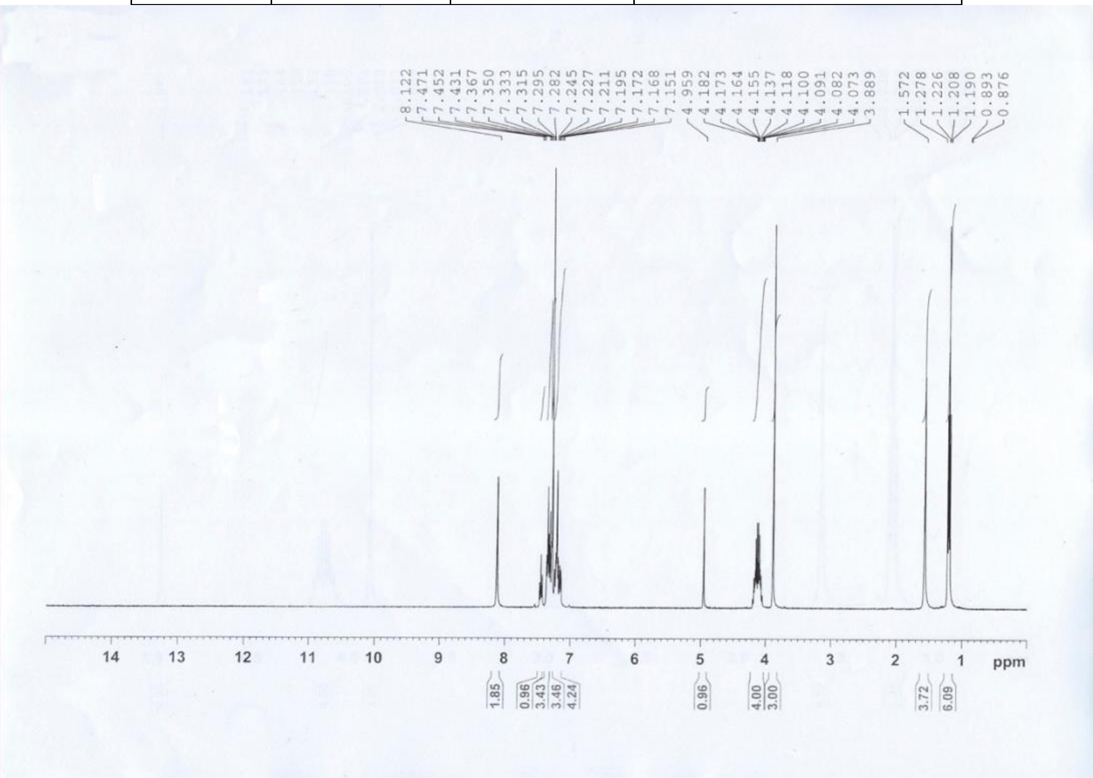

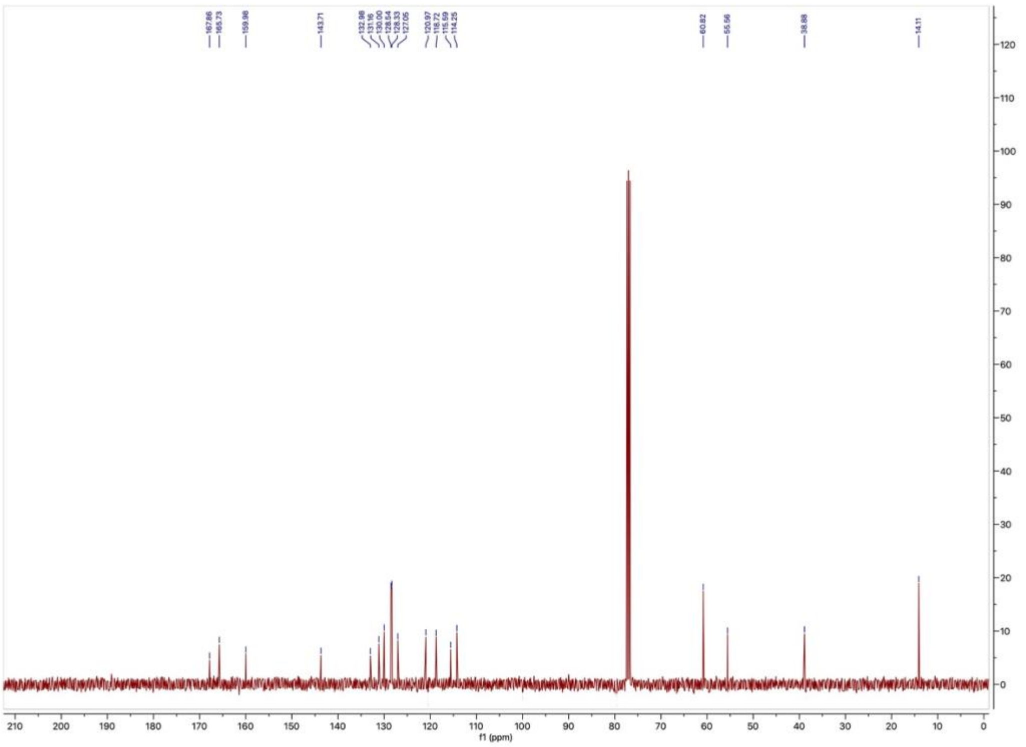

**UBCS396**	**MC4182**	**3b**	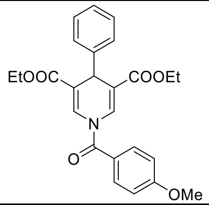	132°C	17,9%

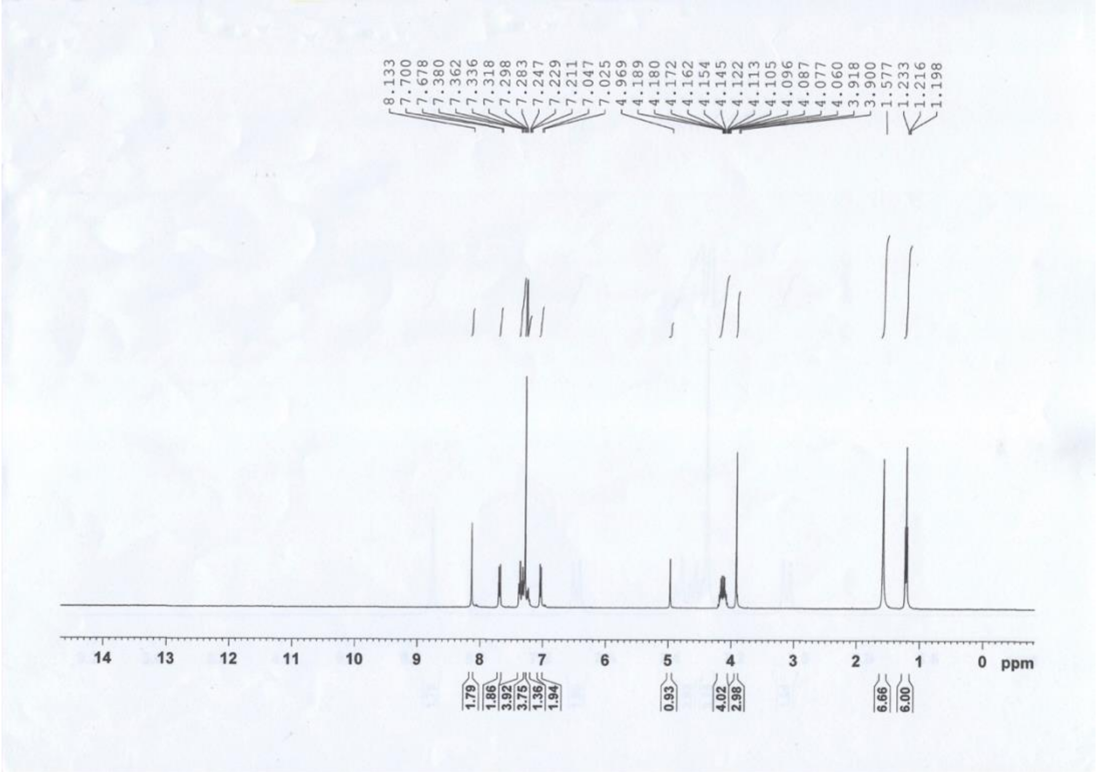

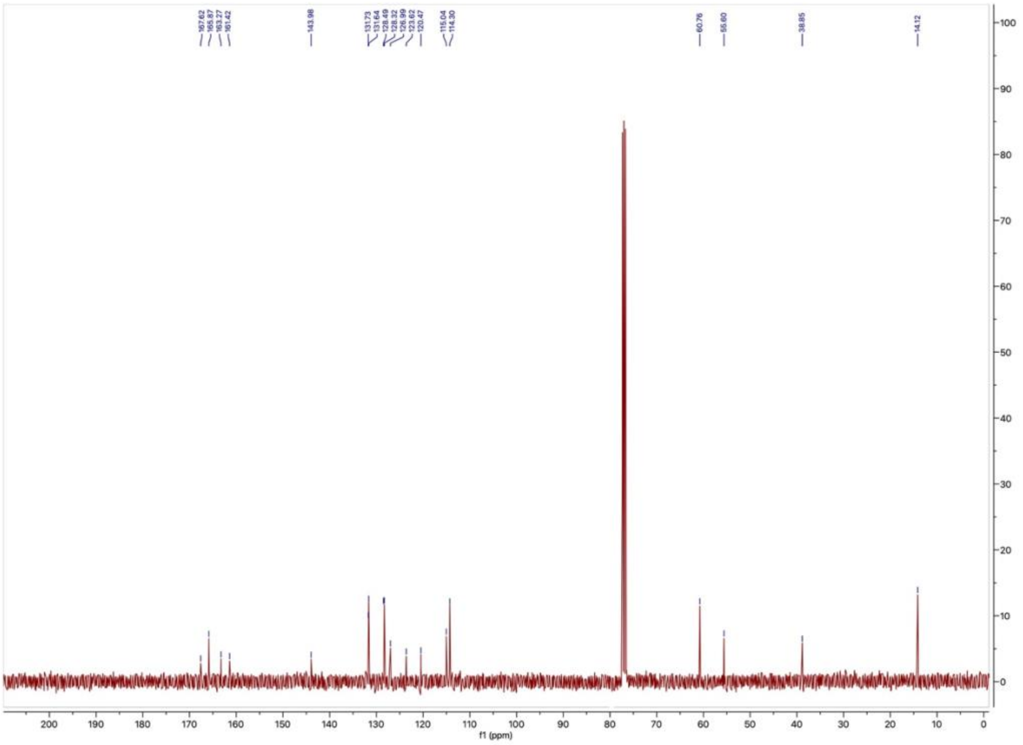

**UBCS398**	**MC4175**	**3c**	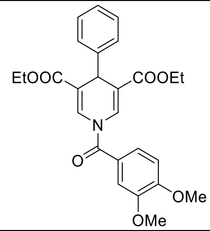

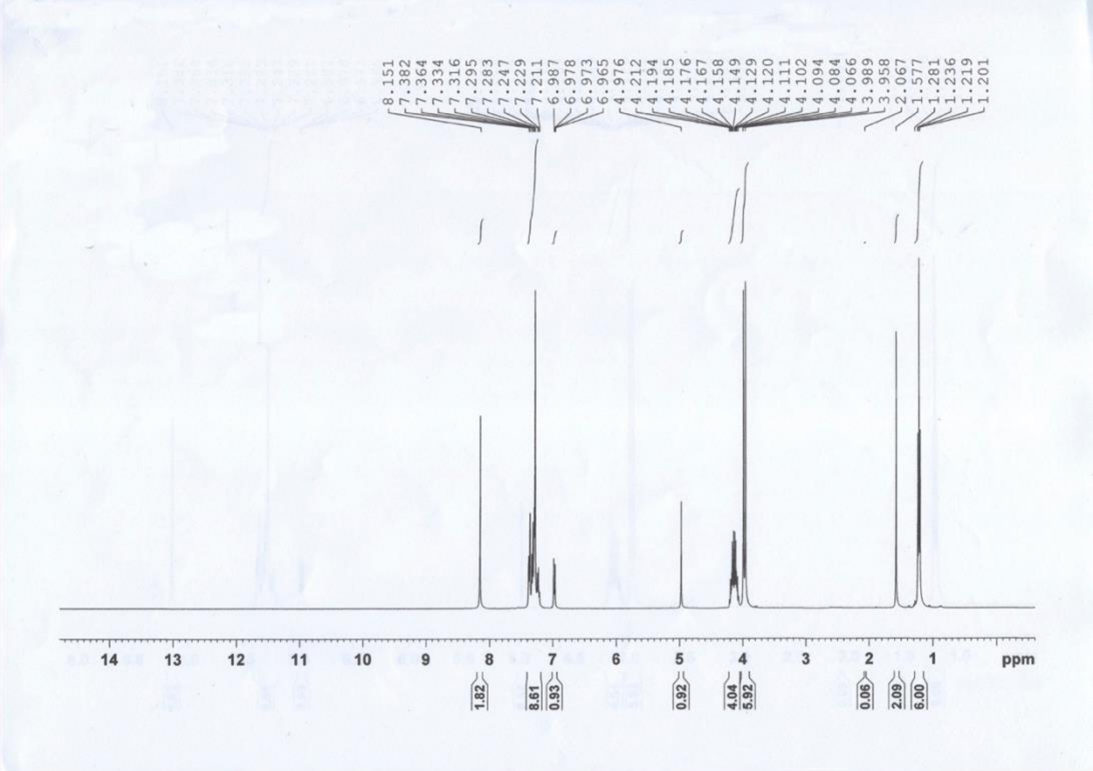

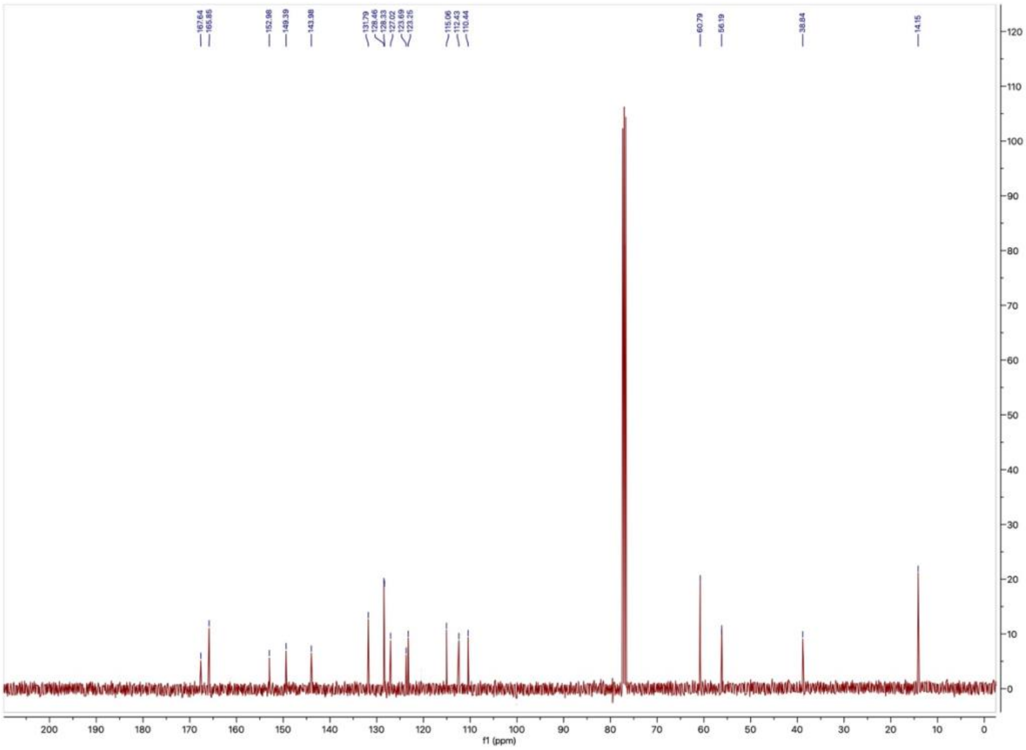

**UBCS397**	**MC4176**	**3d**	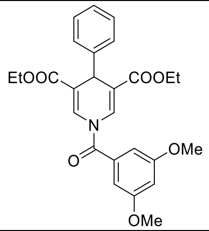	159°C	64, 3%

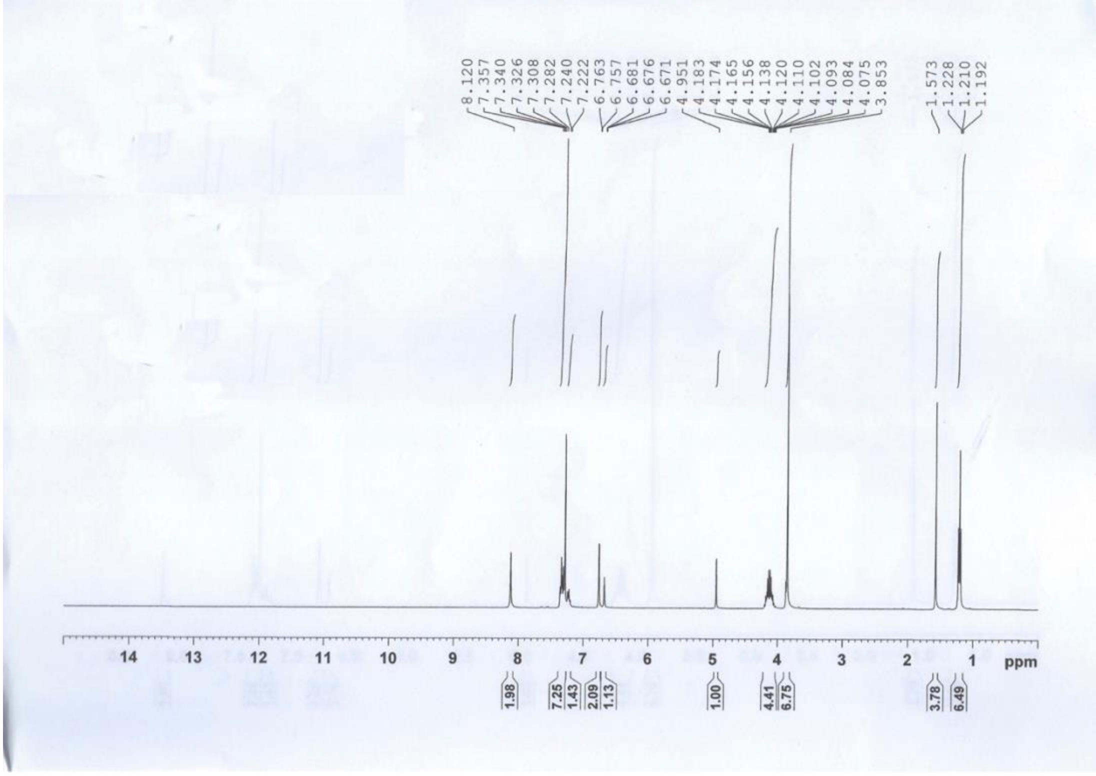

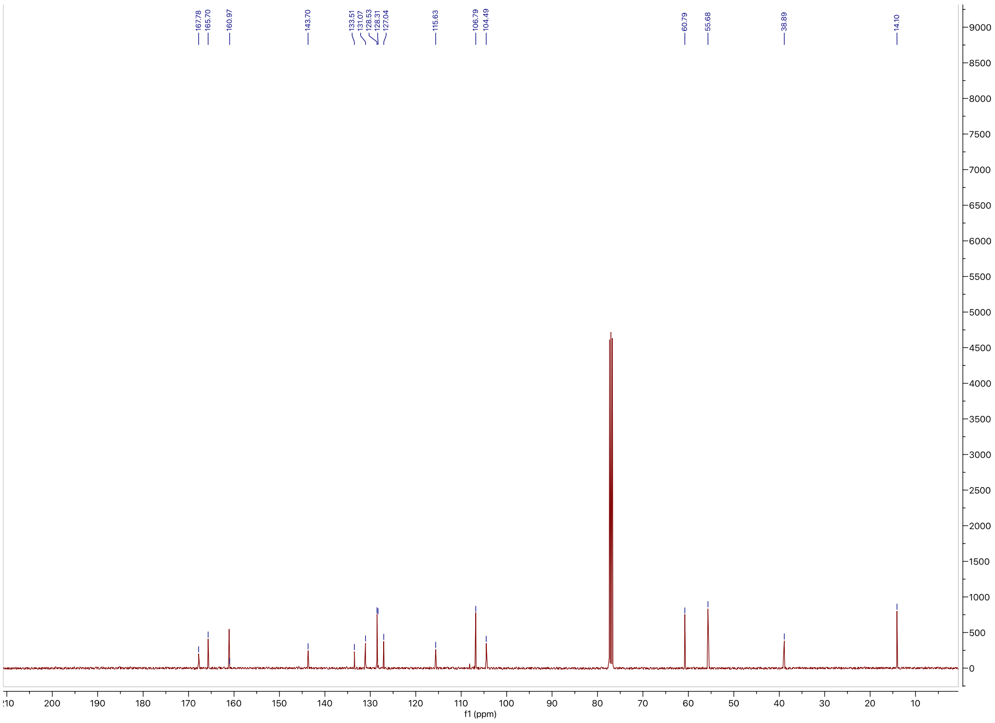

IR spectra and HPLC traces of final compounds 2a-n and 3a-d and high resolution mass spectra for final compounds 3a-d

2a MC4199

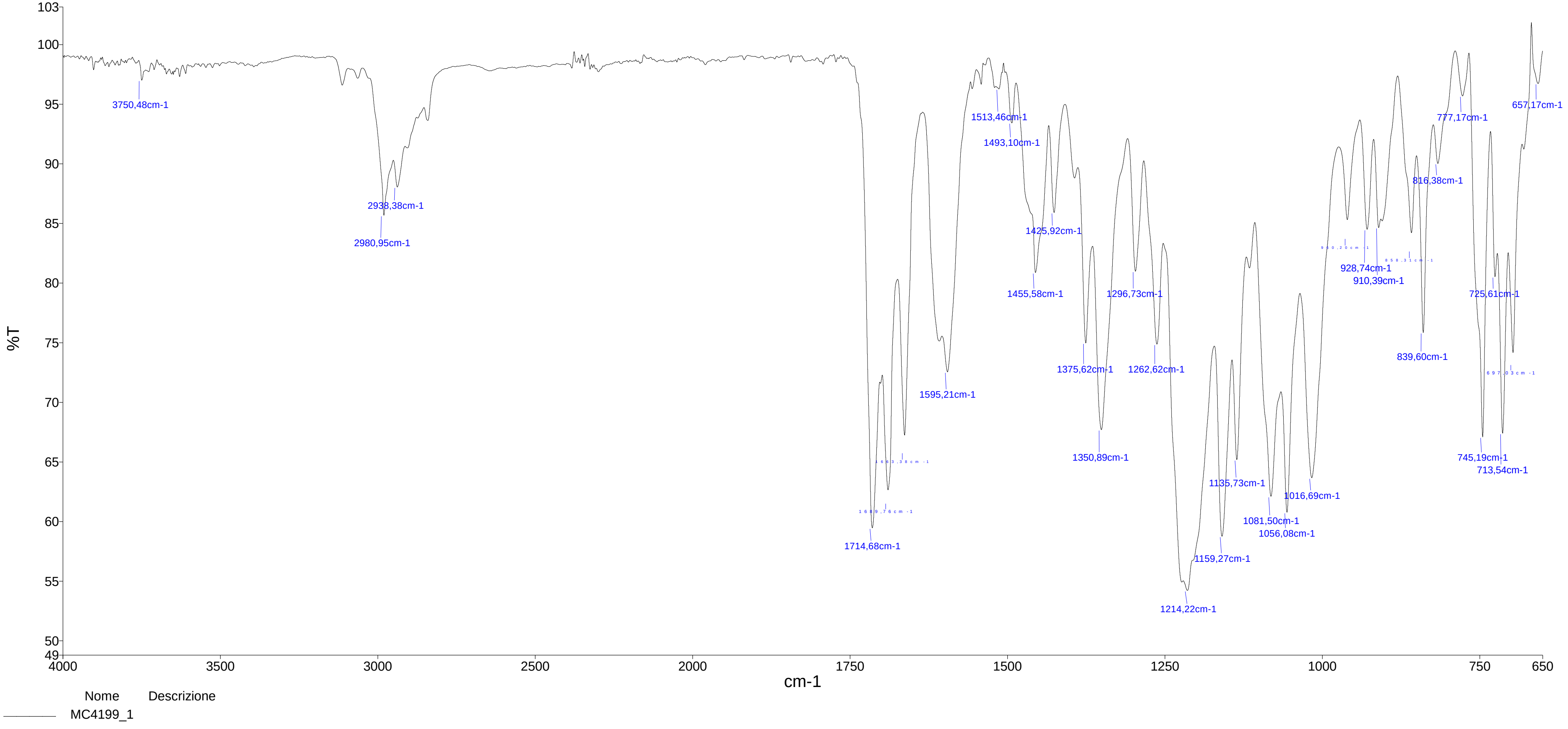

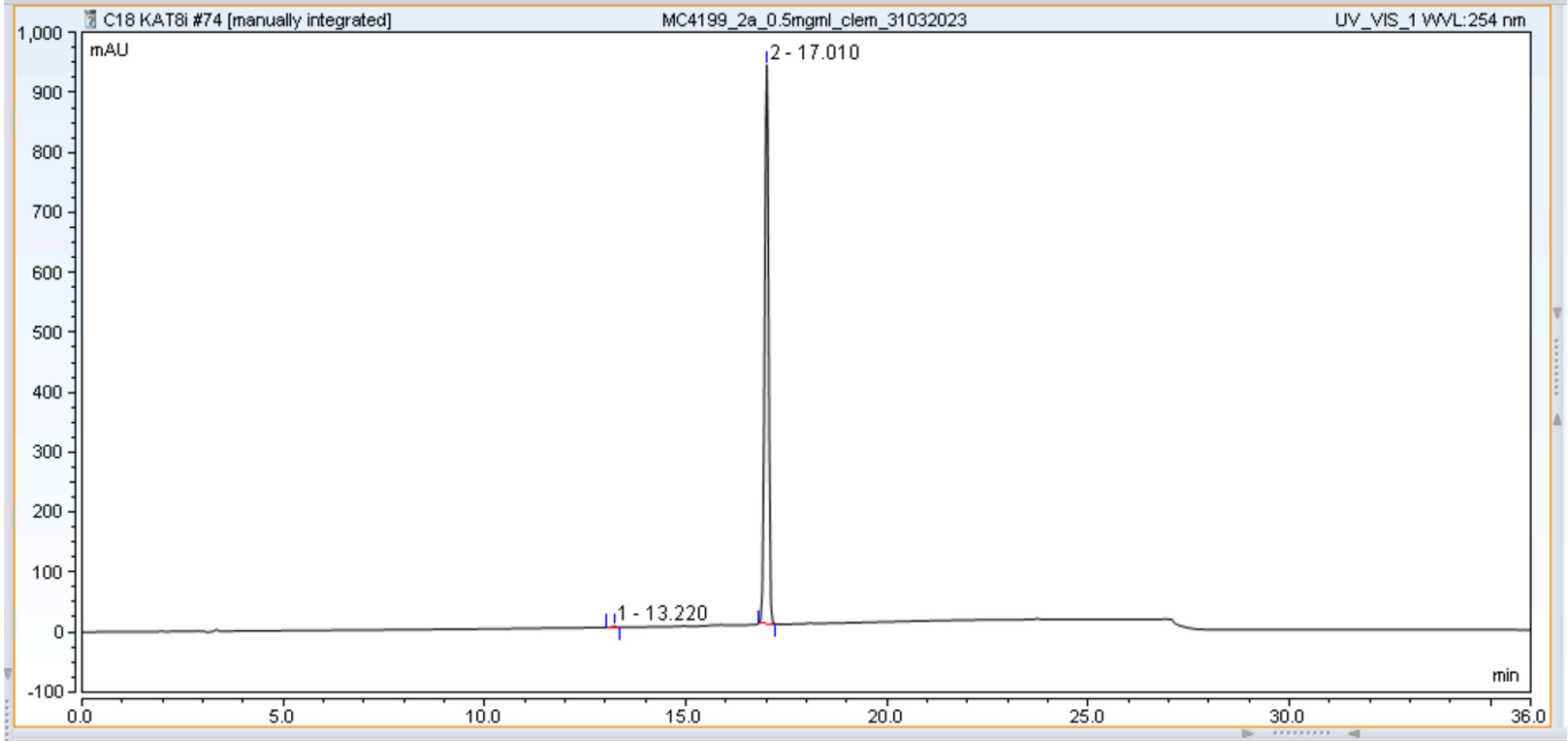

	A	B	C	D	E	F	G	H	I	J	K
1	Peak	Ret.Time	Amount	Rel.Area	Area	Height	Type	Width (50%	Asym.	Resol.	Plates
2	No.	min		%	mAU*min	mAU		min	EP	EP	EP
4	1	13.220	n.a.	0.18	0.1865	1.70	BMB	0.103	0.91	21.53	91718
5	2	17.010	n.a.	99.82	104.0530	932.86	BMB*	0.105	0.94	n.a.	145478
6	Maximum		0.0000	99.82	104.0530	932.86		0.105	0.94	21.53	145478
7	Minimum		0.0000	0.18	0.1865	1.70		0.103	0.91	21.53	91718
8	Sum		0.0000	100.00	104.2396	934.56					
9											

2b MC4158

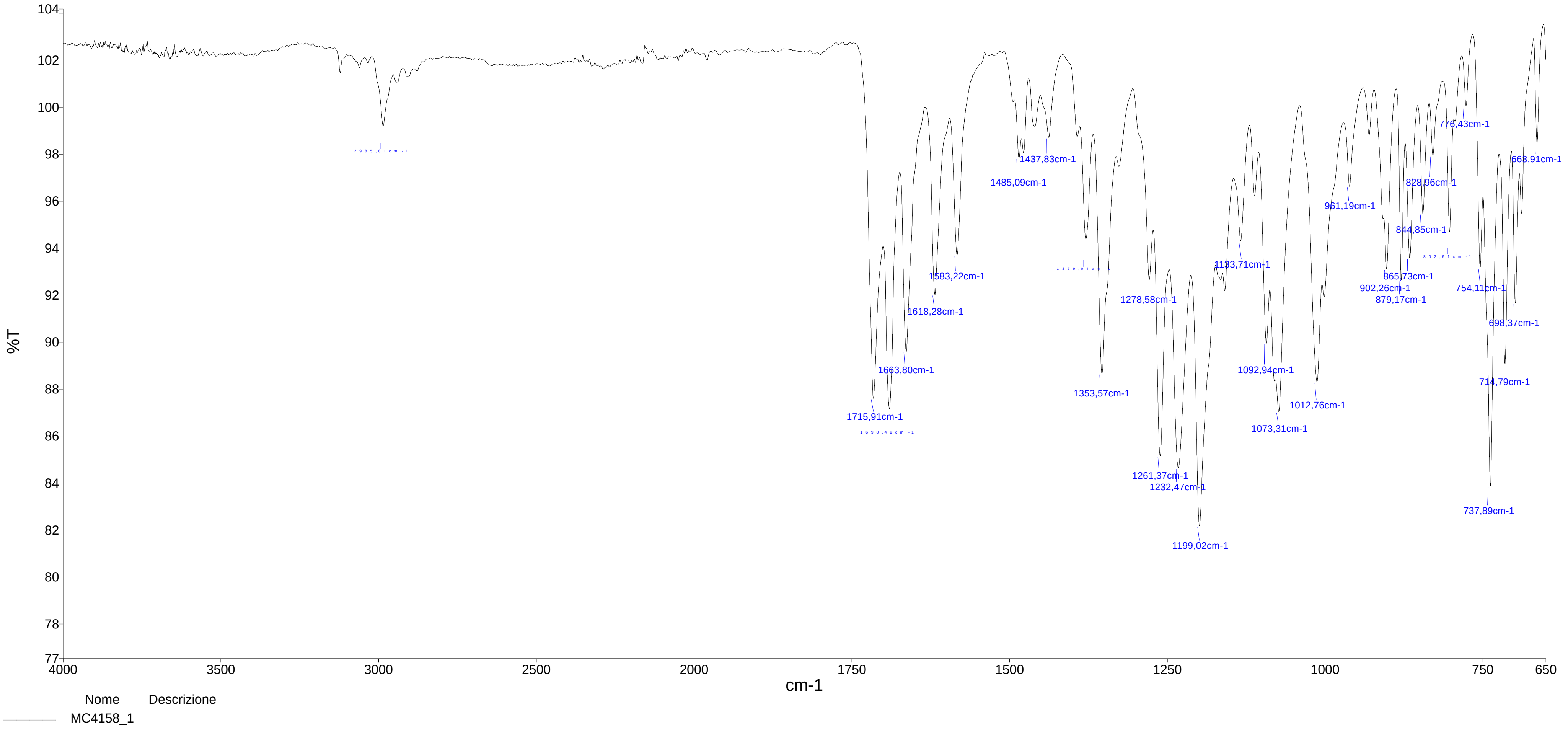
%T
cm-1
1715,91cm-1
1663,80cm-1
1618,28cm-1
1583,22cm-1
1485,09cm-1
1437,83cm-1
1353,57cm-1
1278,58cm-1
1261,37cm-1
1232,47cm-1
1199,02cm-1
1133,71cm-1
1092,94cm-1
1073,31cm-1
1012,76cm-1
961,19cm-1
902,26cm-1
879,17cm-1
865,73cm-1
844,85cm-1
828,96cm-1
776,43cm-1
754,11cm-1
737,89cm-1
714,79cm-1
698,37cm-1
663,91cm-1
Nome Descrizione
MC4158_1

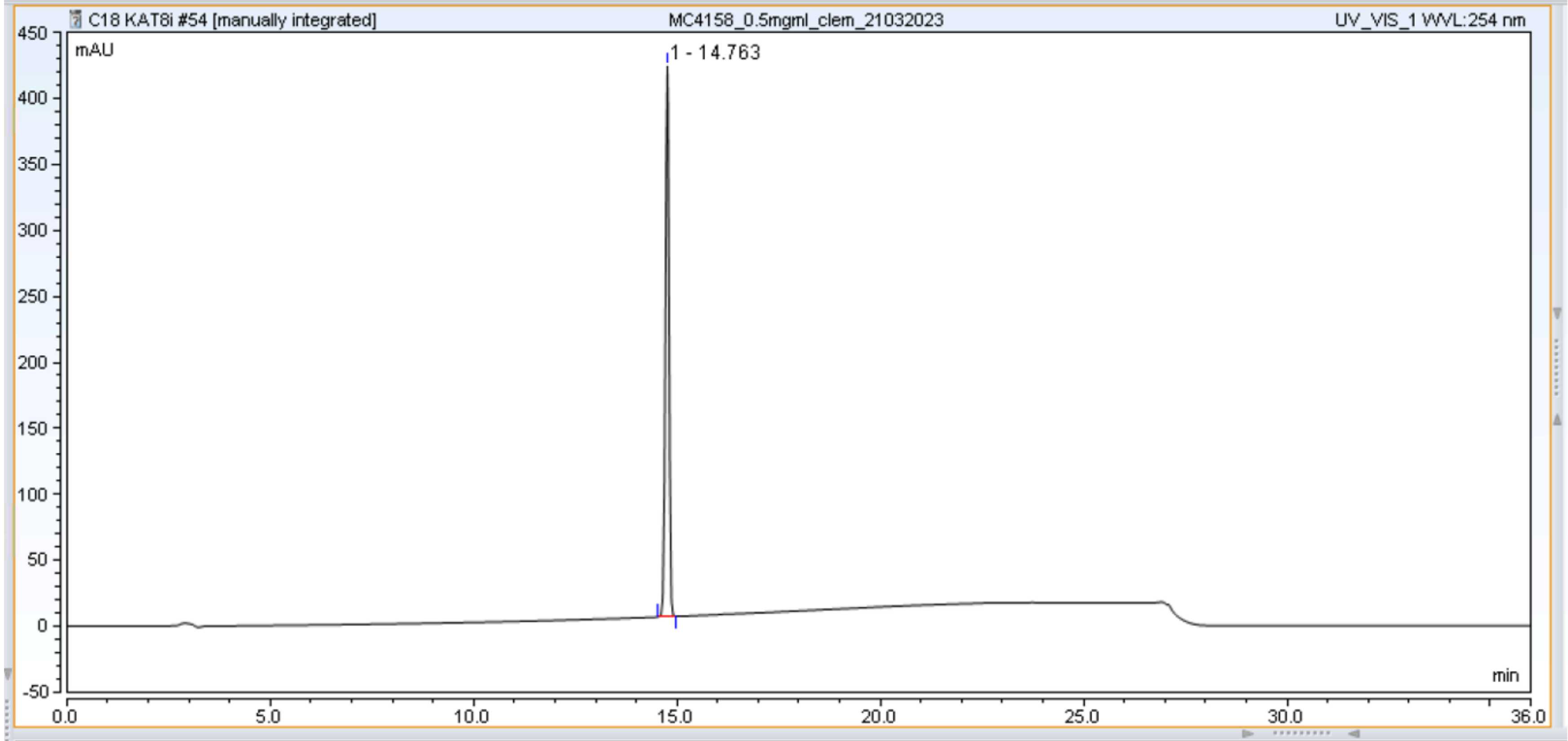

	A	B	C	D	E	F	G	H	I	J	K
1	Peak	Ret.Time	Amount	Rel.Area	Area	Height	Type	Width (50%	Asym.	Resol.	Plates
2	No.	min	n.a.	%	mAU*min	mAU		min	EP	EP	EP
4	1	14.763	n.a.	100.00	42.6207	417.36	BMB*	0.095	0.92	n.a.	133470
6	Maximum		0.0000	100.00	42.6207	417.36		0.095	0.92	0.00	133470
7	Minimum		0.0000	100.00	42.6207	417.36		0.095	0.92	0.00	133470
8	Sum		0.0000	100.00	42.6207	417.36					
9											

2c MC4168

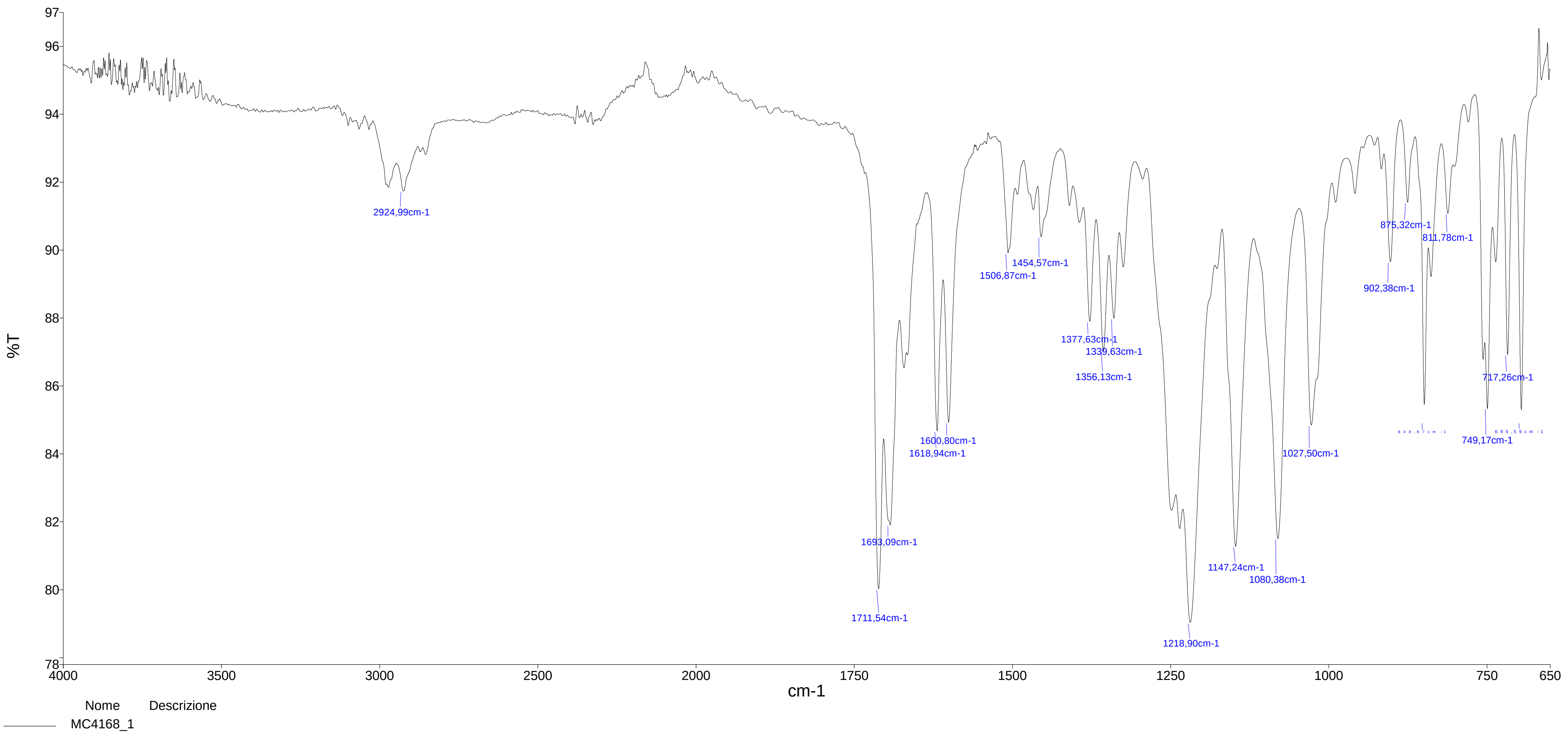
%T
cm-1
2924,99cm-1
1711,54cm-1
1693,09cm-1
1618,94cm-1
1600,80cm-1
1506,87cm-1
1454,57cm-1
1377,63cm-1
1356,13cm-1
1339,63cm-1
1218,90cm-1
1147,24cm-1
1080,38cm-1
1027,50cm-1
902,38cm-1
875,32cm-1
811,78cm-1
749,17cm-1
717,26cm-1
Nome
Descrizione
MC4168_1

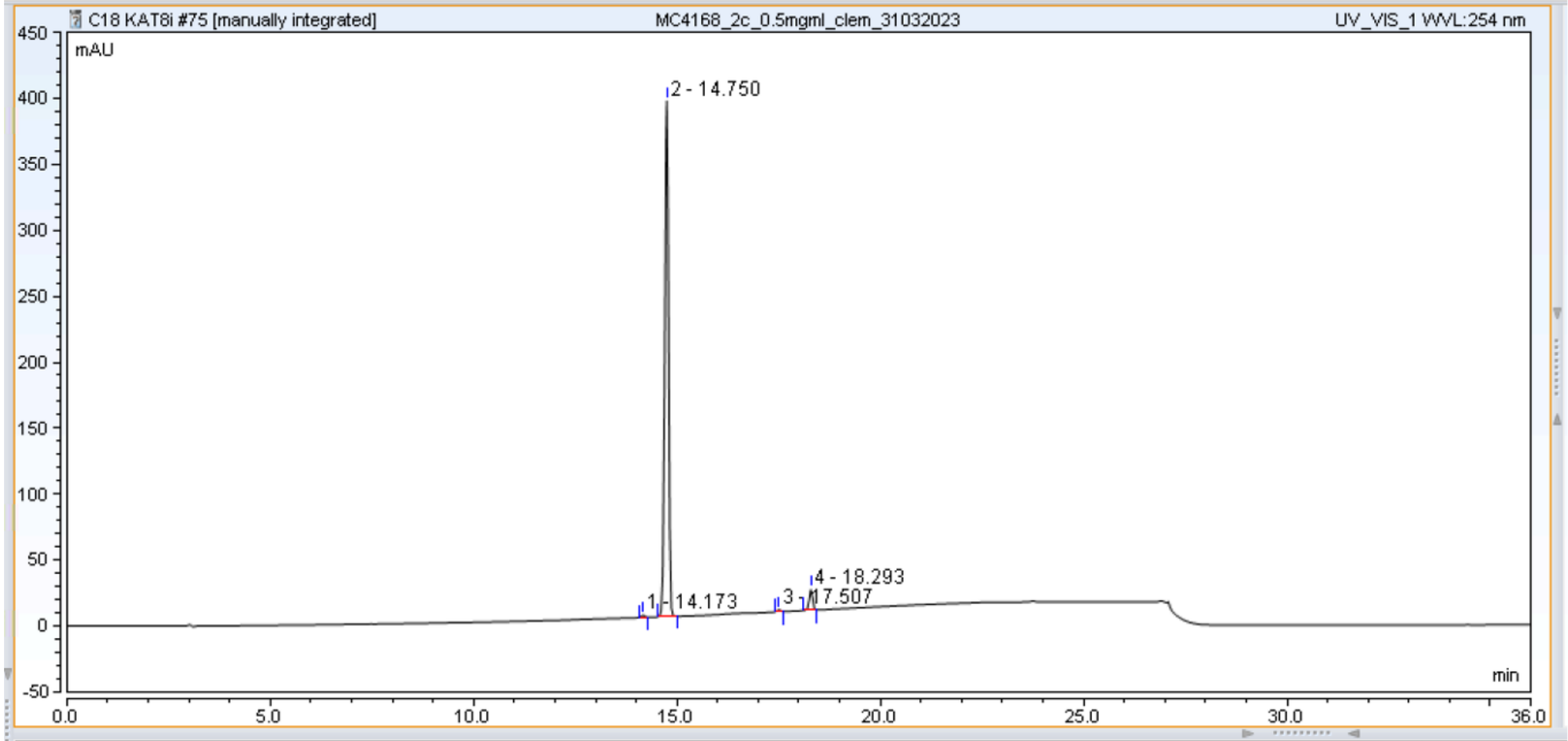

	A	B	C	D	E	F	G	H	I	J	K
1	Peak	Ret.Time	Amount	Rel.Area	Area	Height	Type	Width (50%	Asym.	Resol.	Plates
2	No.	min	n.a.	%	mAU*min	mAU		min	EP	EP	EP
4	1	14.173	n.a.	0.43	0.1898	1.98	BMB*	0.094	0.92	3.51	126697
5	2	14.750	n.a.	95.72	41.7944	391.18	BMB*	0.100	0.92	17.07	120452
6	3	17.507	n.a.	0.34	0.1483	1.57	BMB*	0.091	0.94	5.13	207287
7	4	18.293	n.a.	3.51	1.5306	15.88	BMB*	0.090	0.96	n.a.	227374
8	Maximum		0.0000	95.72	41.7944	391.18		0.100	0.96	17.07	227374
9	Minimum		0.0000	0.34	0.1483	1.57		0.090	0.92	3.51	120452
10	Sum		0.0000	100.00	43.6631	410.61					
11											

2d MC4172

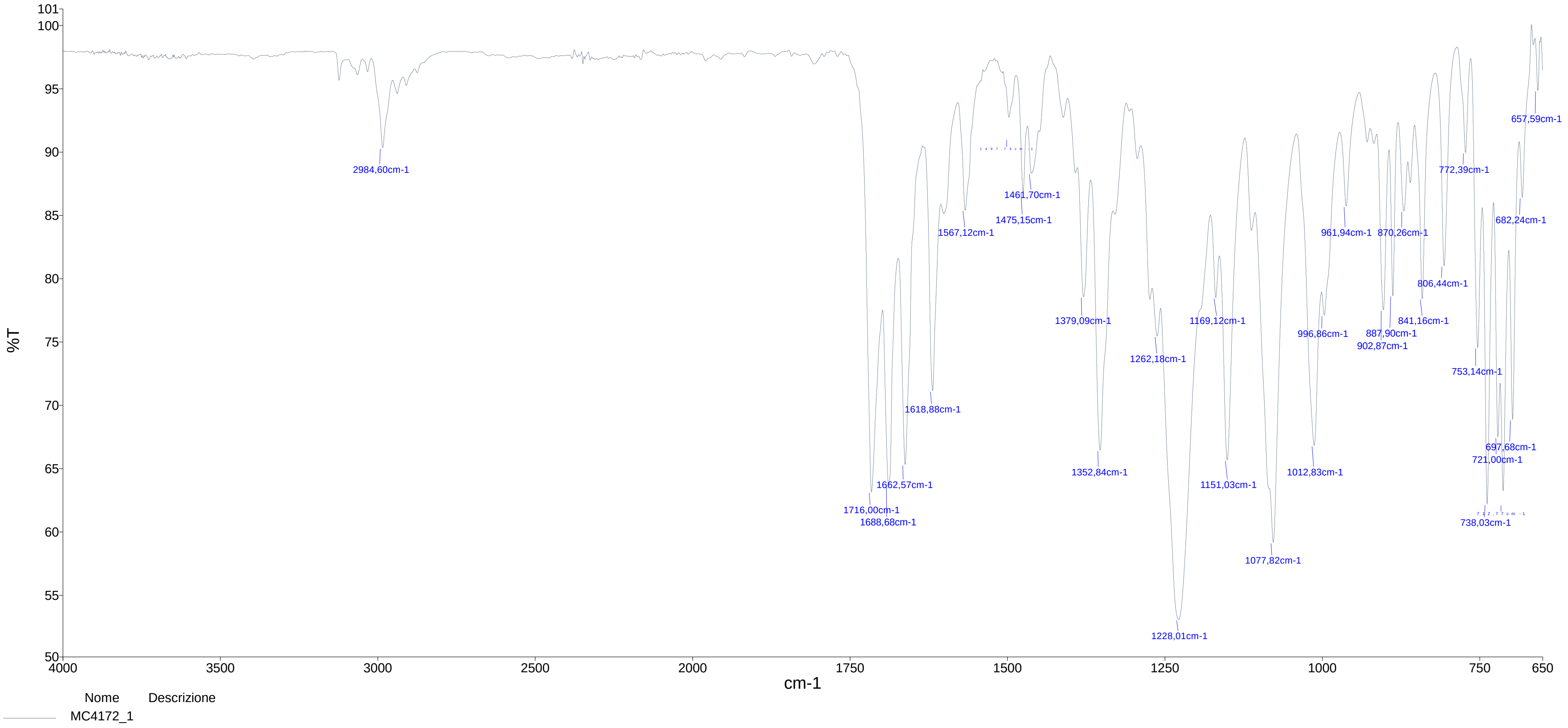

%T
cm-1
2984,60cm-1
1716,00cm-1
1688,68cm-1
1662,57cm-1
1618,88cm-1
1567,12cm-1
1475,15cm-1
1461,70cm-1
1379,09cm-1
1352,84cm-1
1262,18cm-1
1228,01cm-1
1169,12cm-1
1151,03cm-1
1077,82cm-1
1012,83cm-1
996,86cm-1
961,94cm-1
902,87cm-1
887,90cm-1
870,26cm-1
841,16cm-1
806,44cm-1
772,39cm-1
753,14cm-1
738,03cm-1
721,00cm-1
697,68cm-1
682,24cm-1
657,59cm-1
Nome
Descrizione
MC4172_1

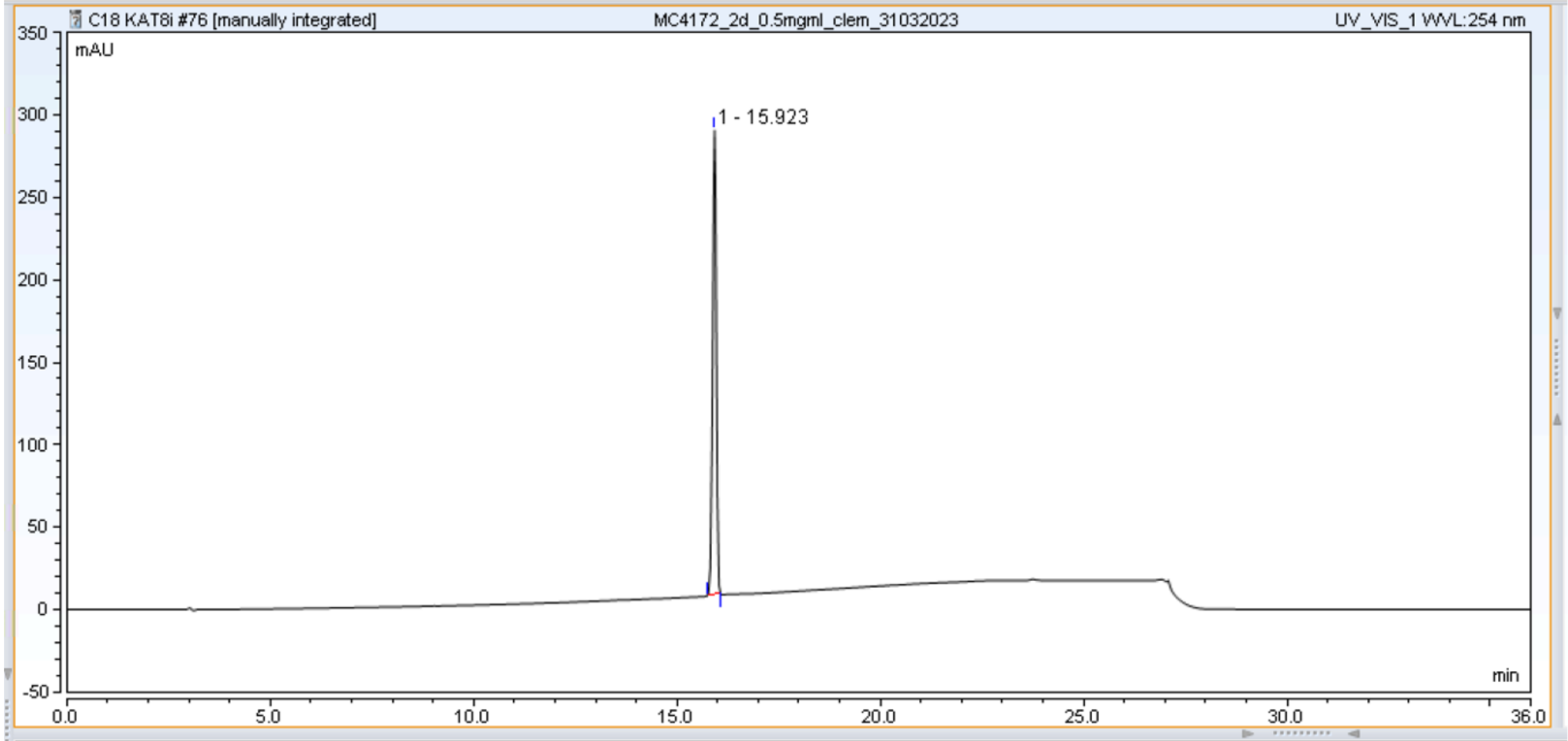

	A	B	C	D	E	F	G	H	I	J	K
1	Peak	Ret.Time	Amount	Rel.Area	Area	Height	Type	Width (50%	Asym.	Resol.	Plates
2	No.	min	n.a.	%	mAU*min	mAU		min	EP	EP	EP
4	1	15.923	n.a.	100.00	29.6950	281.30	BMB*	0.099	0.93	n.a.	142353
6	Maximum		0.0000	100.00	29.6950	281.30		0.099	0.93	0.00	142353
7	Minimum		0.0000	100.00	29.6950	281.30		0.099	0.93	0.00	142353
8	Sum		0.0000	100.00	29.6950	281.30					
9											

2e MC4166

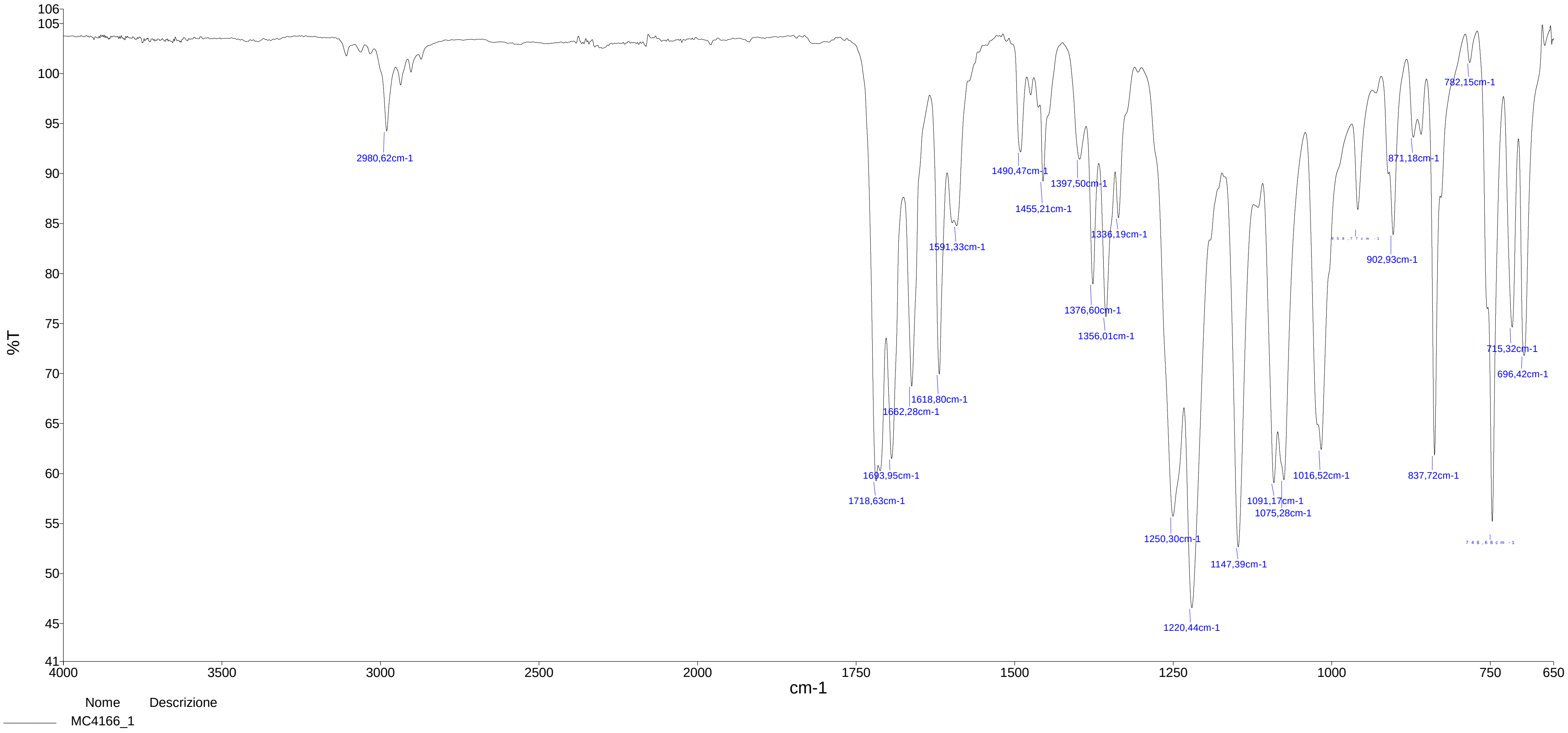

%T
106
105
100
95
90
85
80
75
70
65
60
55
50
45
41
4000
3500
3000
2500
2000
1750
1500
1250
1000
750
650
cm-1
2980,62cm-1
1718,63cm-1
1693,95cm-1
1662,28cm-1
1618,80cm-1
1591,33cm-1
1490,47cm-1
1455,21cm-1
1397,50cm-1
1376,60cm-1
1356,01cm-1
1336,19cm-1
1250,30cm-1
1220,44cm-1
1147,39cm-1
1091,17cm-1
1075,28cm-1
1016,52cm-1
958,77cm-1
902,93cm-1
871,18cm-1
837,72cm-1
782,15cm-1
746,68cm-1
715,32cm-1
696,42cm-1
Nome Descrizione
MC4166_1

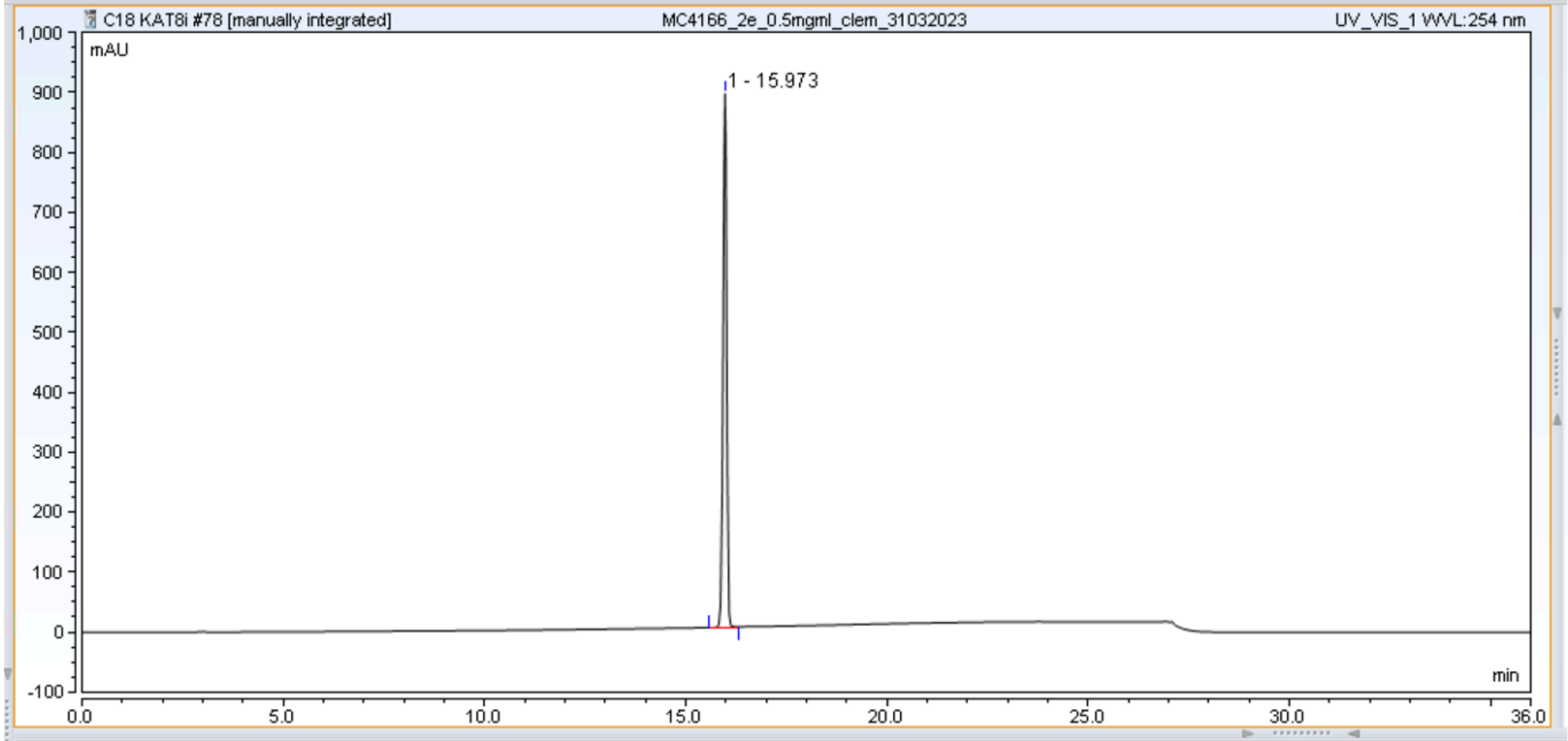

	A	B	C	D	E	F	G	H	I	J	K
1	Peak	Ret.Time	Amount	Rel.Area	Area	Height	Type	Width (50%	Asym.	Resol.	Plates
2	No.	min	n.a.	%	mAU*min	mAU		min	EP	EP	EP
4	1	15.973	n.a.	100.00	92.9384	890.43	BM	0.097	0.92	n.a.	150105
6	Maximum		0.0000	100.00	92.9384	890.43		0.097	0.92	0.00	150105
7	Minimum		0.0000	100.00	92.9384	890.43		0.097	0.92	0.00	150105
8	Sum		0.0000	100.00	92.9384	890.43					
9											

2f MC4181

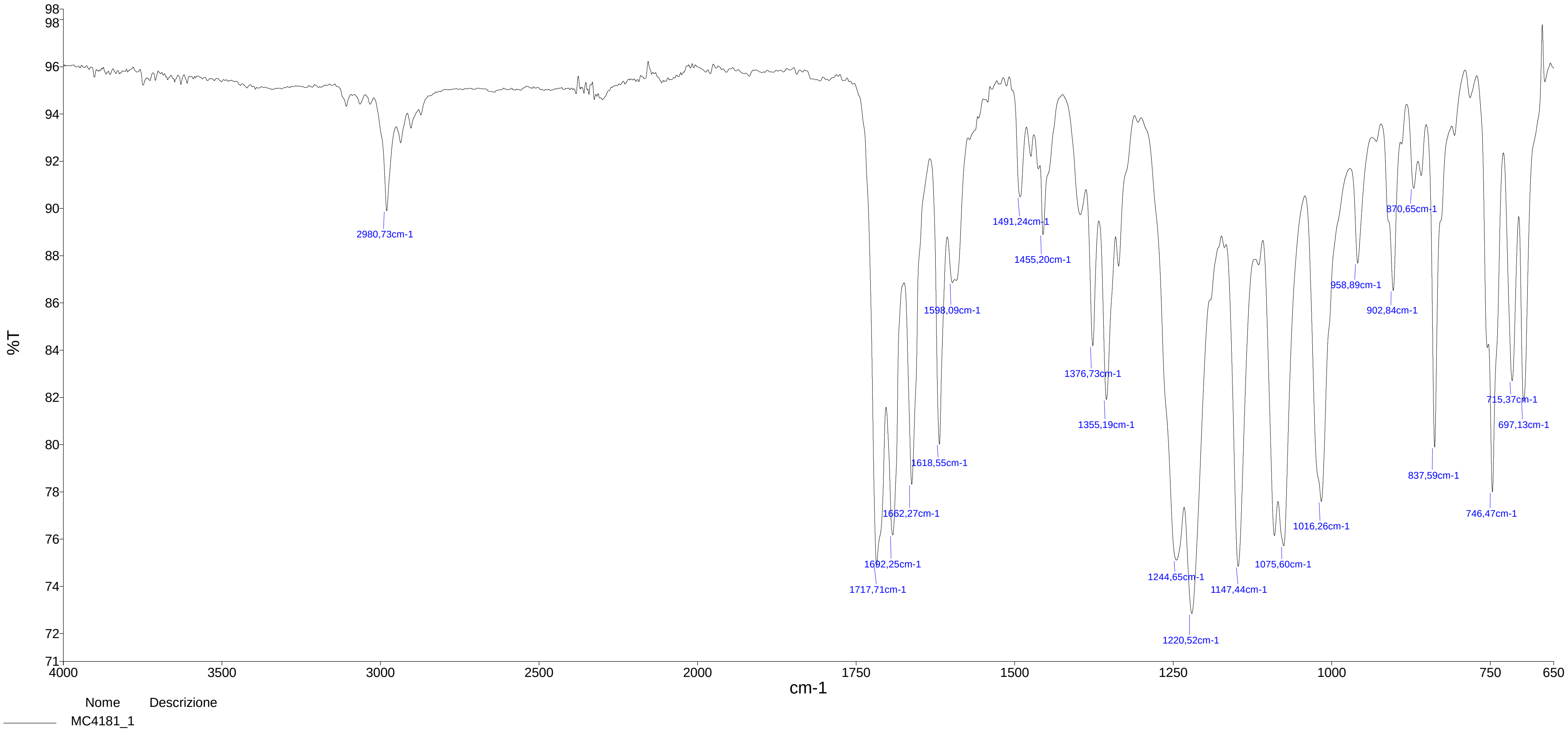

%T
cm-1
2980,73cm-1
1717,71cm-1
1692,25cm-1
1662,27cm-1
1618,55cm-1
1598,09cm-1
1491,24cm-1
1455,20cm-1
1376,73cm-1
1355,19cm-1
1244,65cm-1
1220,52cm-1
1147,44cm-1
1075,60cm-1
1016,26cm-1
958,89cm-1
902,84cm-1
870,65cm-1
837,59cm-1
746,47cm-1
715,37cm-1
697,13cm-1
Nome Descrizione
MC4181_1

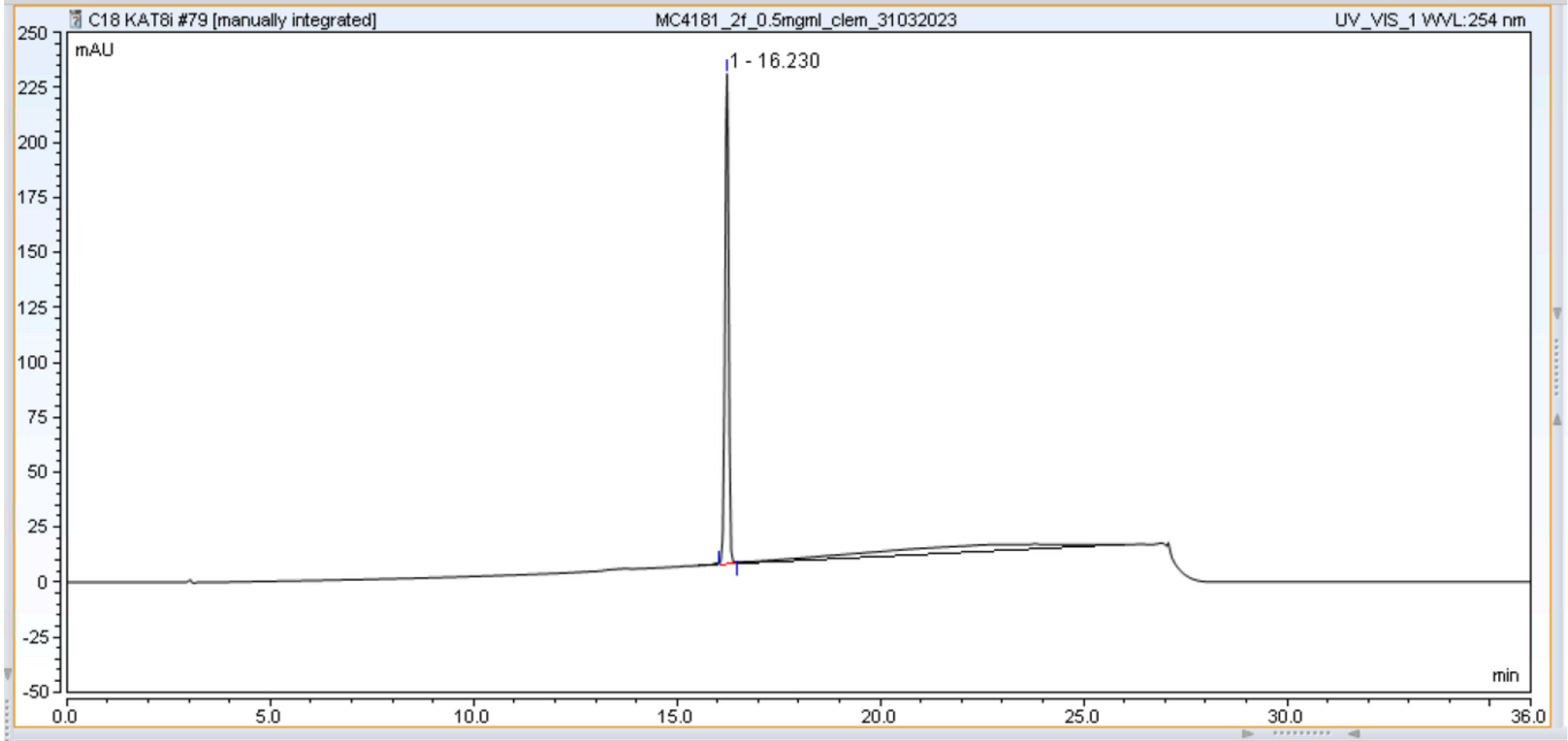

	A	B	C	D	E	F	G	H	I	J	K
1	Peak	Ret.Time	Amount	Rel.Area	Area	Height	Type	Width (50%	Asym.	Resol.	Plates
2	No.	min	n.a.	%	mAU*min	mAU		min	EP	EP	EP
4	1	16.230	n.a.	100.00	23.6146	223.14	M	0.098	0.92	n.a.	150911
6	Maximum		0.0000	100.00	23.6146	223.14		0.098	0.92	0.00	150911
7	Minimum		0.0000	100.00	23.6146	223.14		0.098	0.92	0.00	150911
8	Sum		0.0000	100.00	23.6146	223.14					
9											

2g MC4185

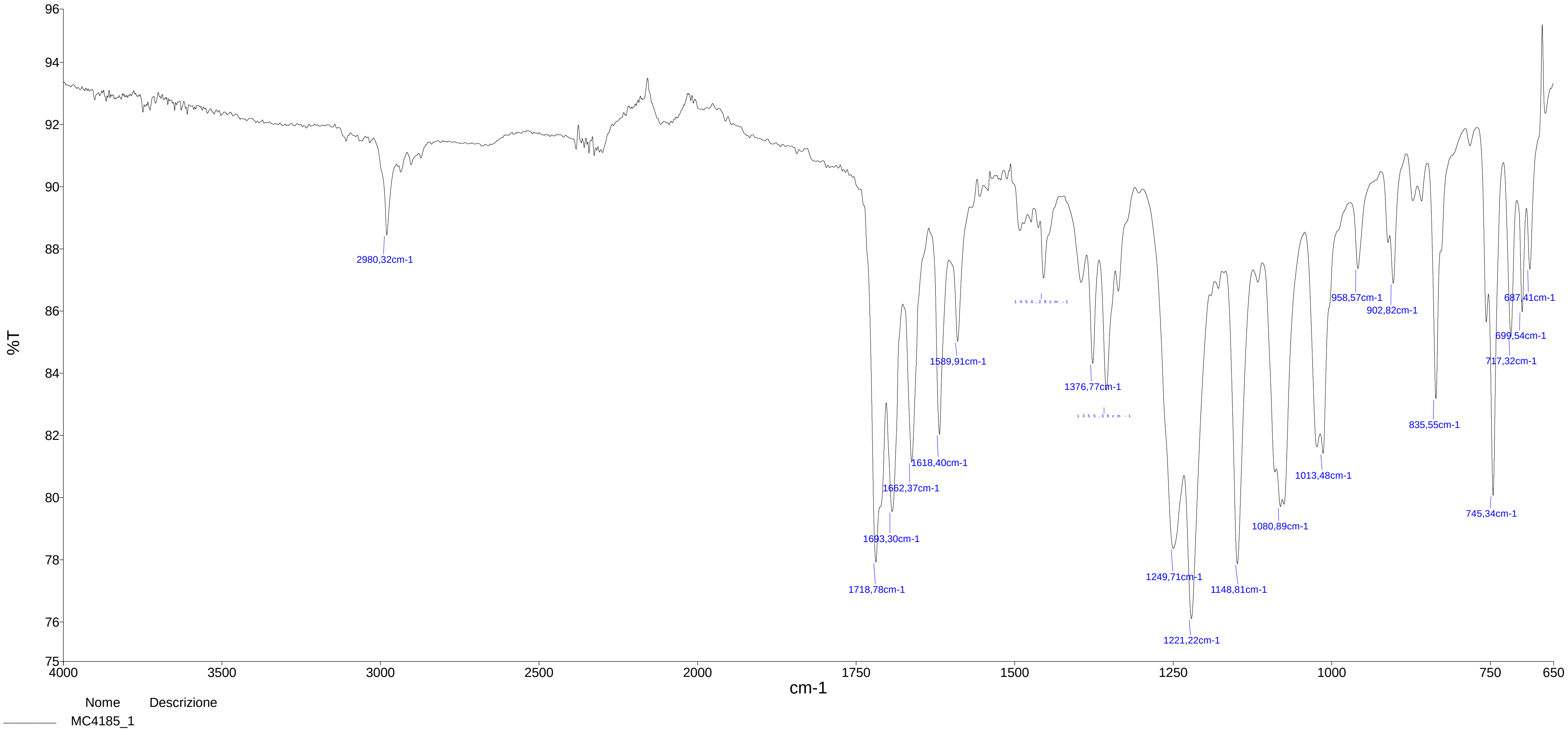

%T
96
94
92
90
88
86
84
82
80
78
76
75
4000
3500
3000
2500
2000
1750
1500
1250
1000
750
650
cm-1
2980,32cm-1
1718,78cm-1
1693,30cm-1
1662,37cm-1
1618,40cm-1
1589,91cm-1
1454,29cm-1
1376,77cm-1
1355,39cm-1
1249,71cm-1
1221,22cm-1
1148,81cm-1
1080,89cm-1
1013,48cm-1
958,57cm-1
902,82cm-1
835,55cm-1
745,34cm-1
717,32cm-1
699,54cm-1
687,41cm-1
Nome
Descrizione
MC4185_1

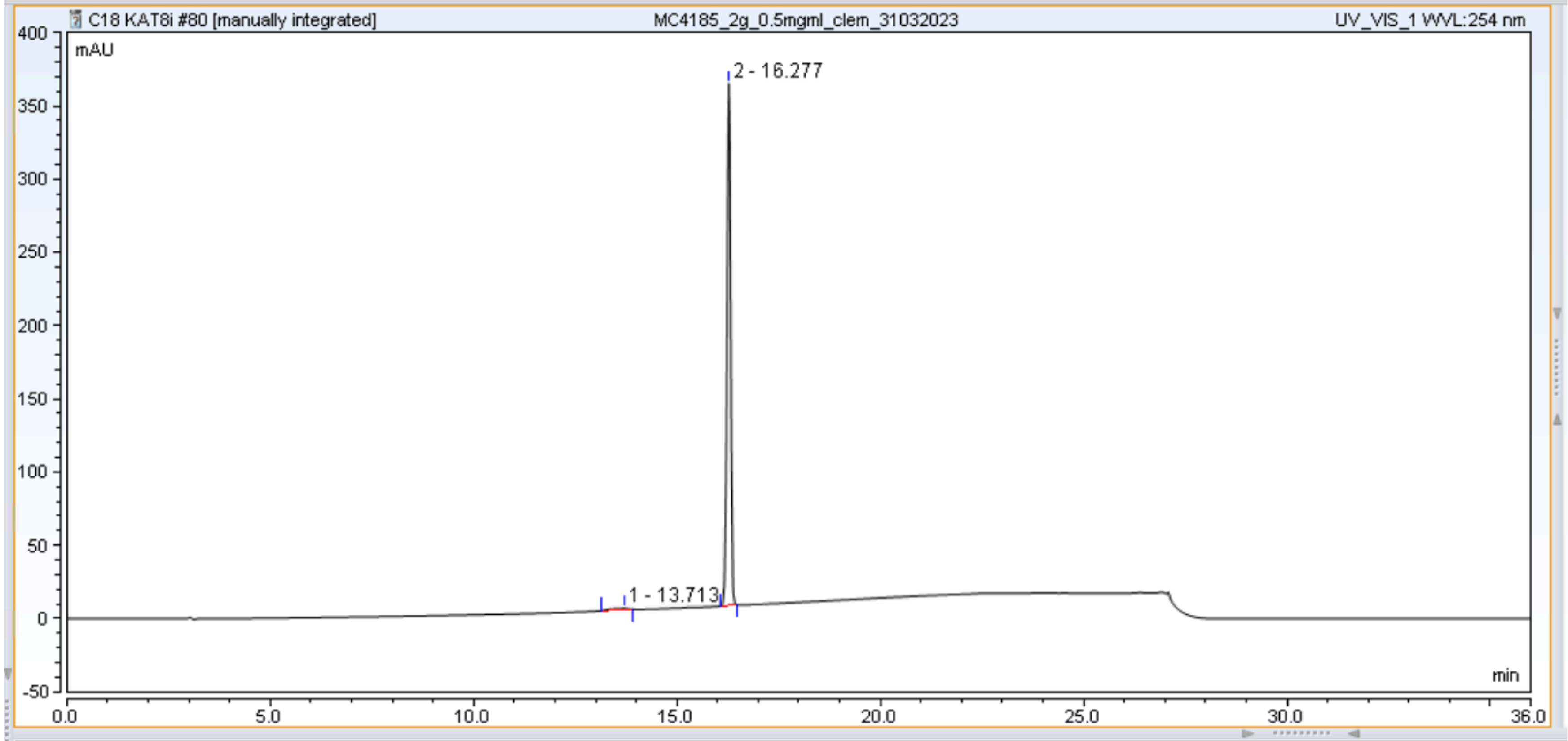

	A	B	C	D	E	F	G	H	I	J	K
1	Peak	Ret.Time	Amount	Rel.Area	Area	Height	Type	Width (50%	Asym.	Resol.	Plates
2	No.	min	n.a.	%	mAU*min	mAU		min	EP	EP	EP
4	1	13.713	n.a.	1.40	0.5283	0.86	BMB*	0.597	0.67	4.36	2926
5	2	16.277	n.a.	98.60	37.2587	356.38	BMB*	0.098	0.93	n.a.	153624
6	Maximum		0.0000	98.60	37.2587	356.38		0.597	0.93	4.36	153624
7	Minimum		0.0000	1.40	0.5283	0.86		0.098	0.67	4.36	2926
8	Sum		0.0000	100.00	37.7869	357.24					
9											

2h MC4165

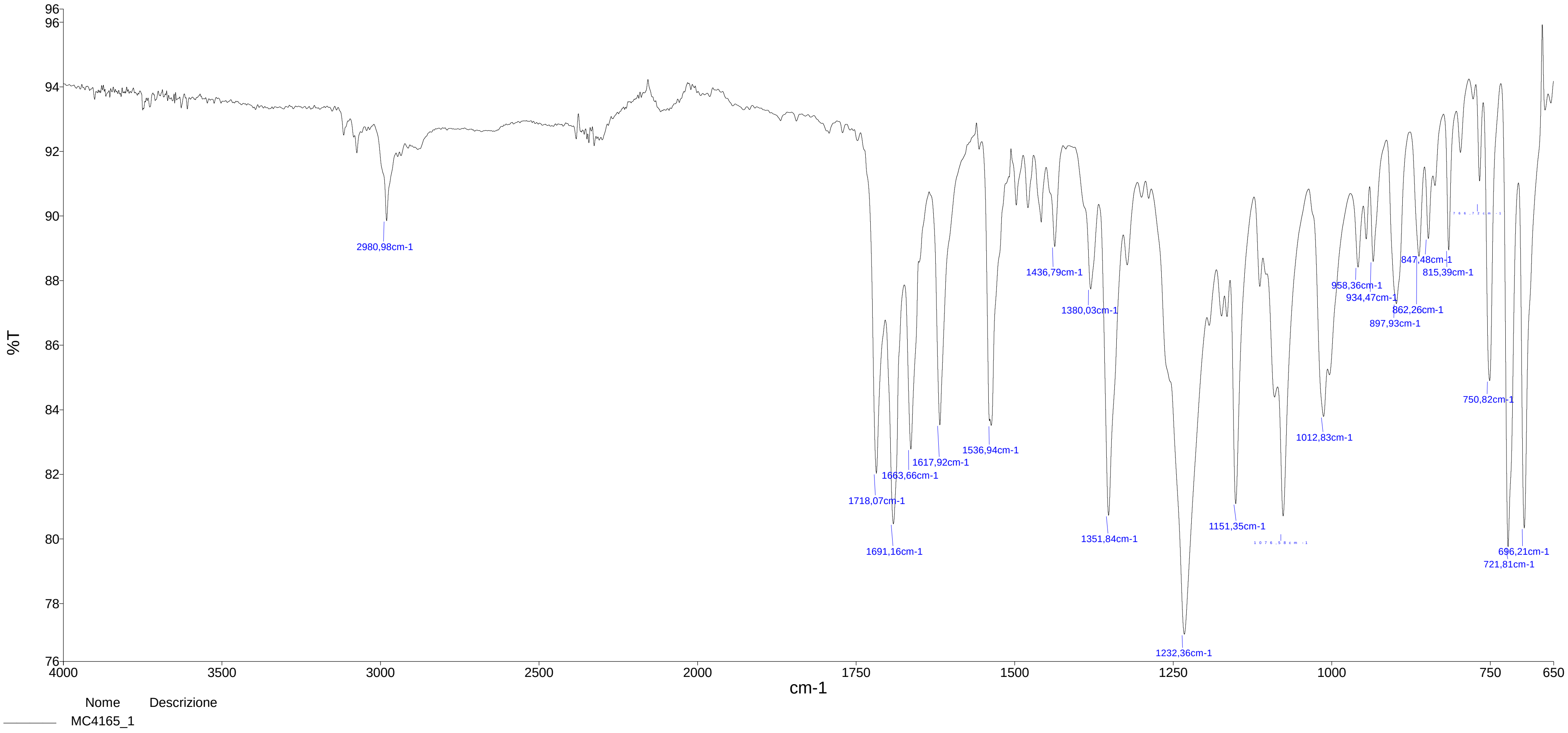
%T
cm-1
2980,98cm-1
1718,07cm-1
1691,16cm-1
1663,66cm-1
1617,92cm-1
1536,94cm-1
1436,79cm-1
1380,03cm-1
1351,84cm-1
1232,36cm-1
1151,35cm-1
1012,83cm-1
958,36cm-1
934,47cm-1
897,93cm-1
862,26cm-1
847,48cm-1
815,39cm-1
750,82cm-1
721,81cm-1
696,21cm-1
Nome
Descrizione
MC4165_1

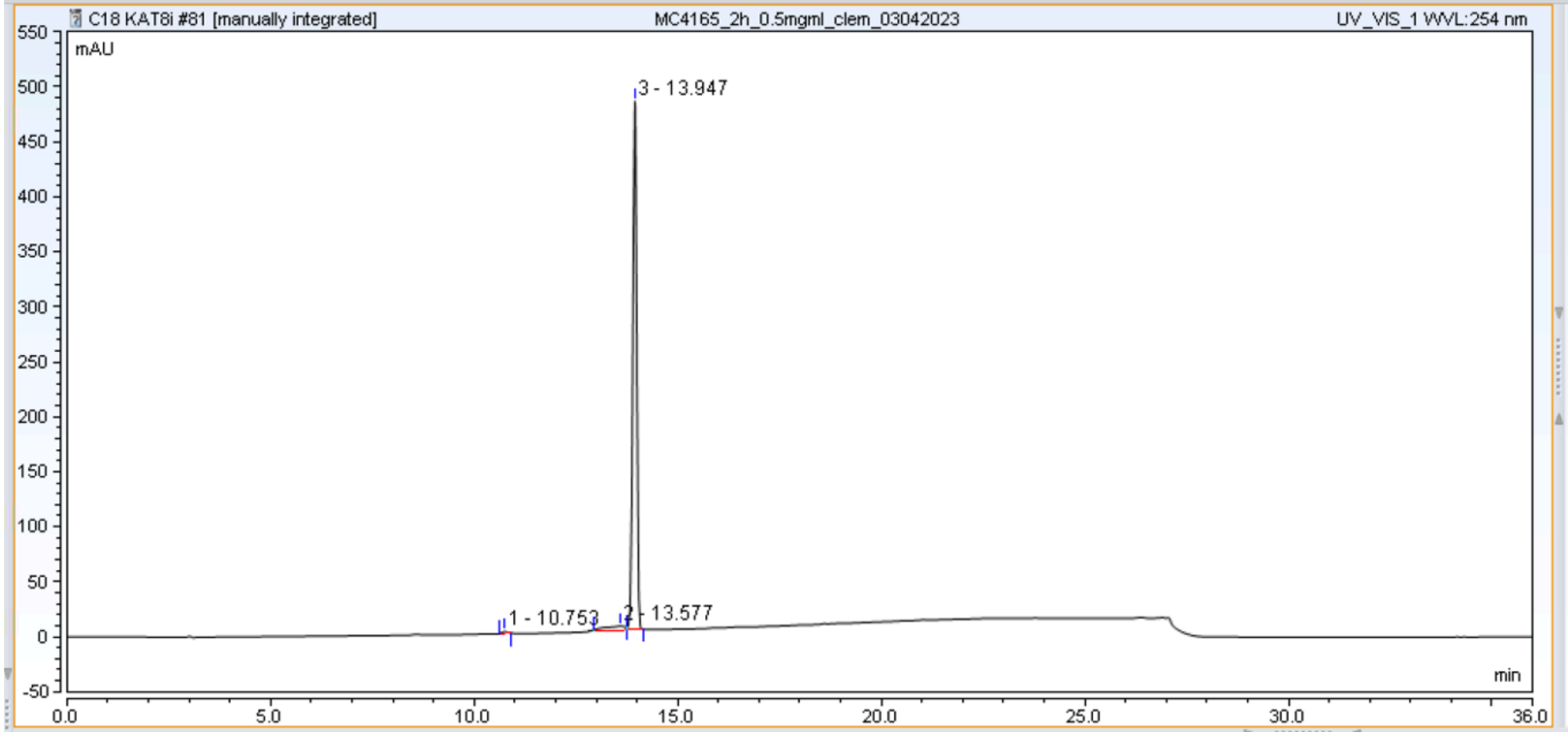

	A	B	C	D	E	F	G	H	I	J	K
1	Peak	Ret.Time	Amount	Rel.Area	Area	Height	Type	Width (50%	Asym.	Resol.	Plates
2	No.	min	n.a.	%	mAU*min	mAU		min	EP	EP	EP
4	1	10.753	n.a.	0.48	0.2688	1.66	BMB*	0.162	1.08	n.a.	24371
5	2	13.577	n.a.	4.29	2.4166	3.79	M *	n.a.	n.a.	n.a.	n.a.
6	3	13.947	n.a.	95.23	53.6503	480.57	MB*	0.105	0.93	n.a.	97269
7	Maximum		0.0000	95.23	53.6503	480.57		0.162	1.08	0.00	97269
8	Minimum		0.0000	0.48	0.2688	1.66		0.105	0.93	0.00	24371
9	Sum		0.0000	100.00	56.3357	486.02					
10											

2i MC4167

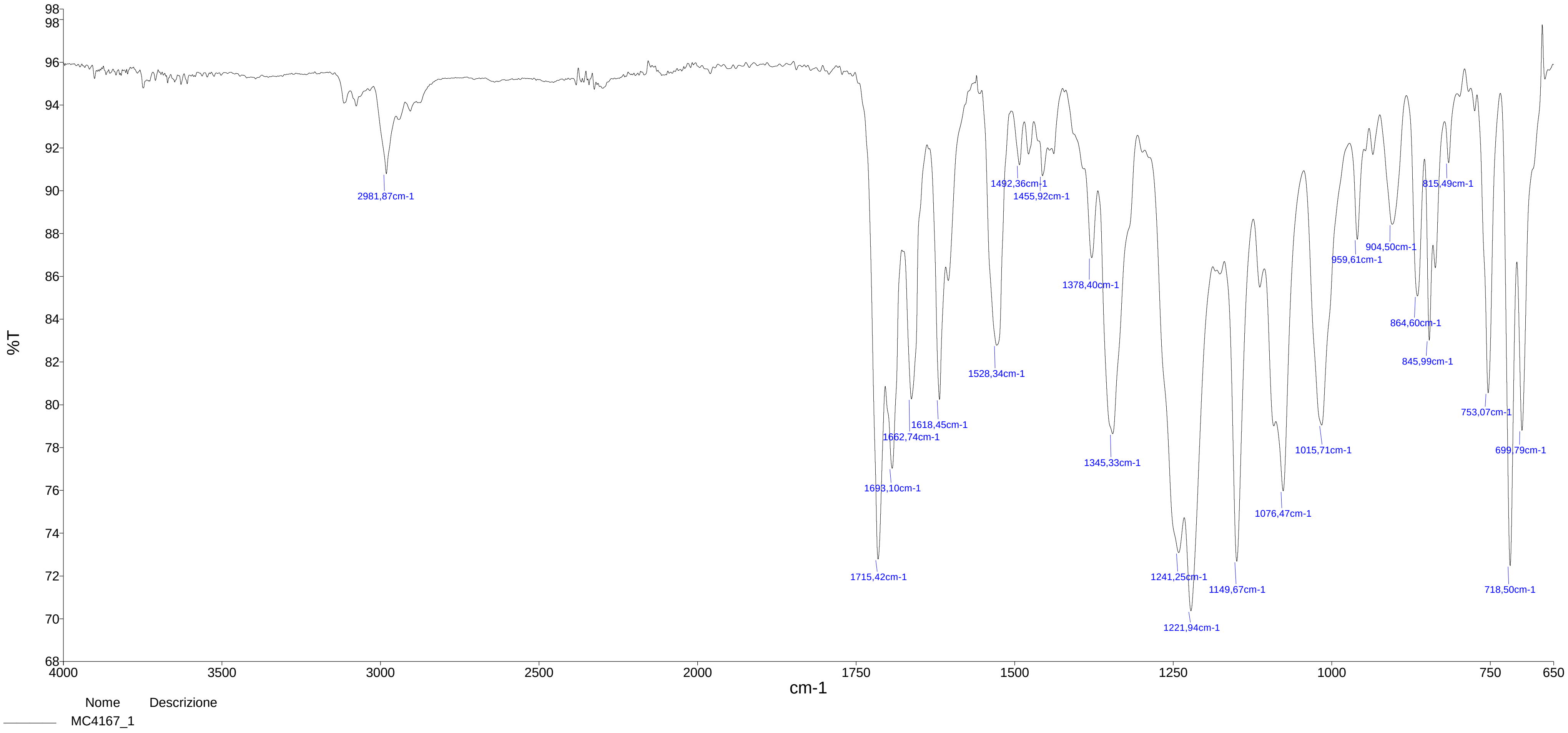

%T
cm-1
2981,87cm-1
1715,42cm-1
1693,10cm-1
1662,74cm-1
1618,45cm-1
1528,34cm-1
1492,36cm-1
1455,92cm-1
1378,40cm-1
1345,33cm-1
1241,25cm-1
1221,94cm-1
1149,67cm-1
1076,47cm-1
1015,71cm-1
959,61cm-1
904,50cm-1
864,60cm-1
845,99cm-1
815,49cm-1
753,07cm-1
718,50cm-1
699,79cm-1
Nome
Descrizione
MC4167_1

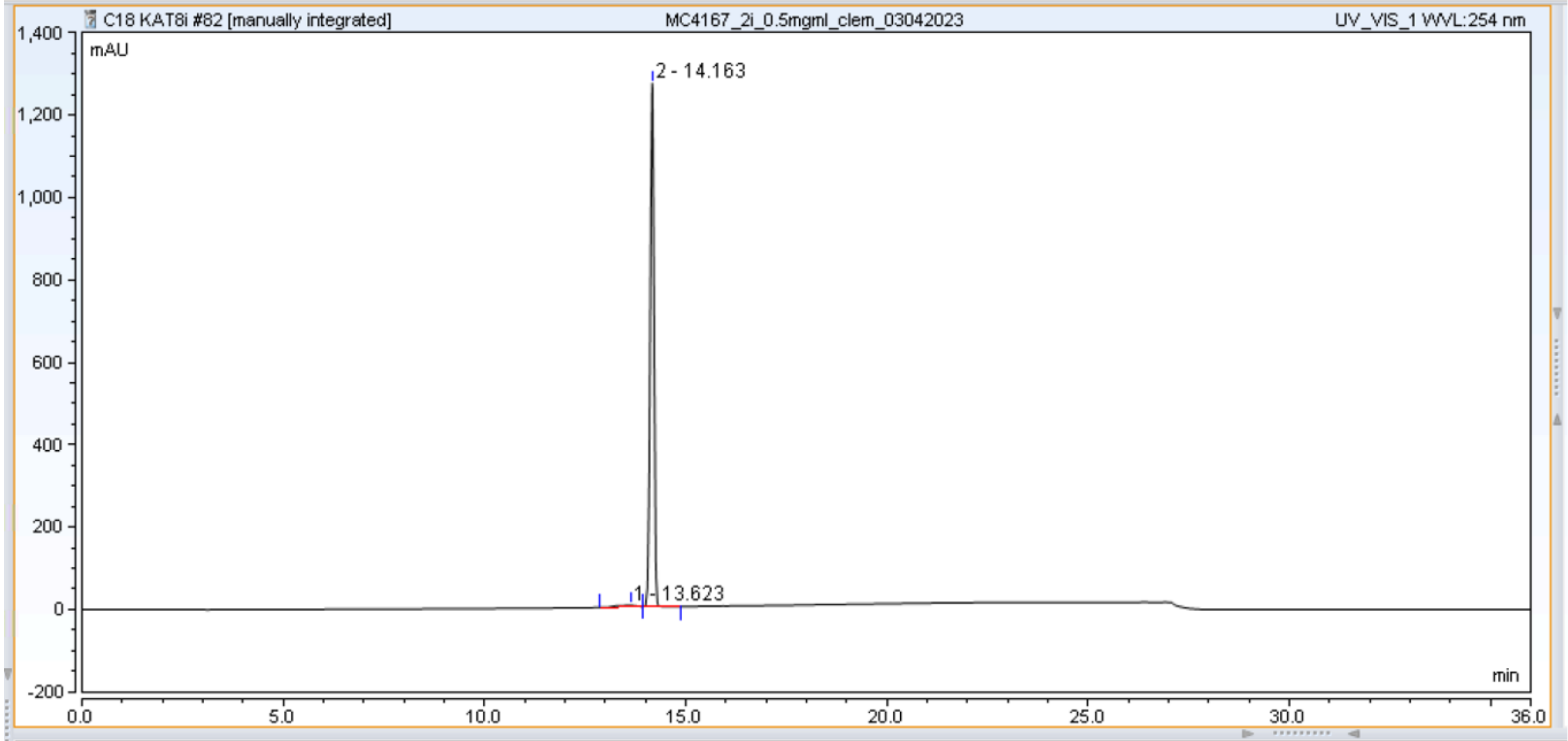

	A	B	C	D	E	F	G	H	I	J	K
1	Peak	Ret.Time	Amount	Rel.Area	Area	Height	Type	Width (50%	Asym.	Resol.	Plates
2	No.	min	n.a.	%	mAU*min	mAU		min	EP	EP	EP
4	1	13.623	n.a.	1.90	2.7416	4.18	BM	0.655	n.a.	0.84	2395
5	2	14.163	n.a.	98.10	141.2413	1270.74	MB	0.105	0.93	n.a.	101341
6	Maximum		0.0000	98.10	141.2413	1270.74		0.655	0.93	0.84	101341
7	Minimum		0.0000	1.90	2.7416	4.18		0.105	0.93	0.84	2395
8	Sum		0.0000	100.00	143.9829	1274.92					
9											

2j MC4177

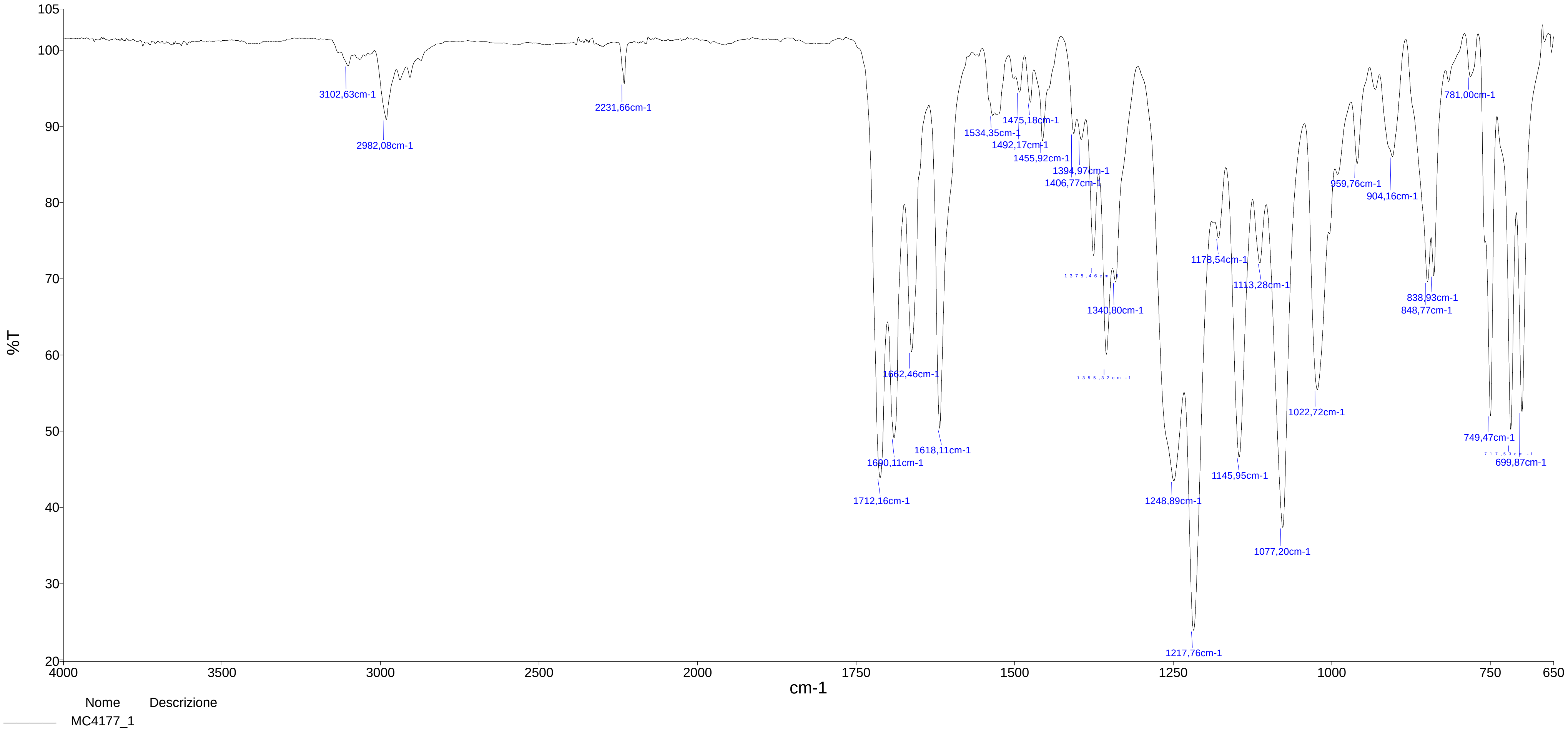

105
100
90
80
70
60
50
40
30
20
%T
4000
3500
3000
2500
2000
1750
1500
1250
1000
750
650
cm-1
3102,63cm-1
2982,08cm-1
2231,66cm-1
1712,16cm-1
1690,11cm-1
1662,46cm-1
1618,11cm-1
1534,35cm-1
1492,17cm-1
1475,18cm-1
1455,92cm-1
1406,77cm-1
1394,97cm-1
1375,46cm-1
1355,32cm-1
1340,80cm-1
1248,89cm-1
1217,76cm-1
1178,54cm-1
1145,95cm-1
1113,28cm-1
1077,20cm-1
1022,72cm-1
959,76cm-1
904,16cm-1
848,77cm-1
838,93cm-1
781,00cm-1
749,47cm-1
717,53cm-1
699,87cm-1
Nome
Descrizione
MC4177_1

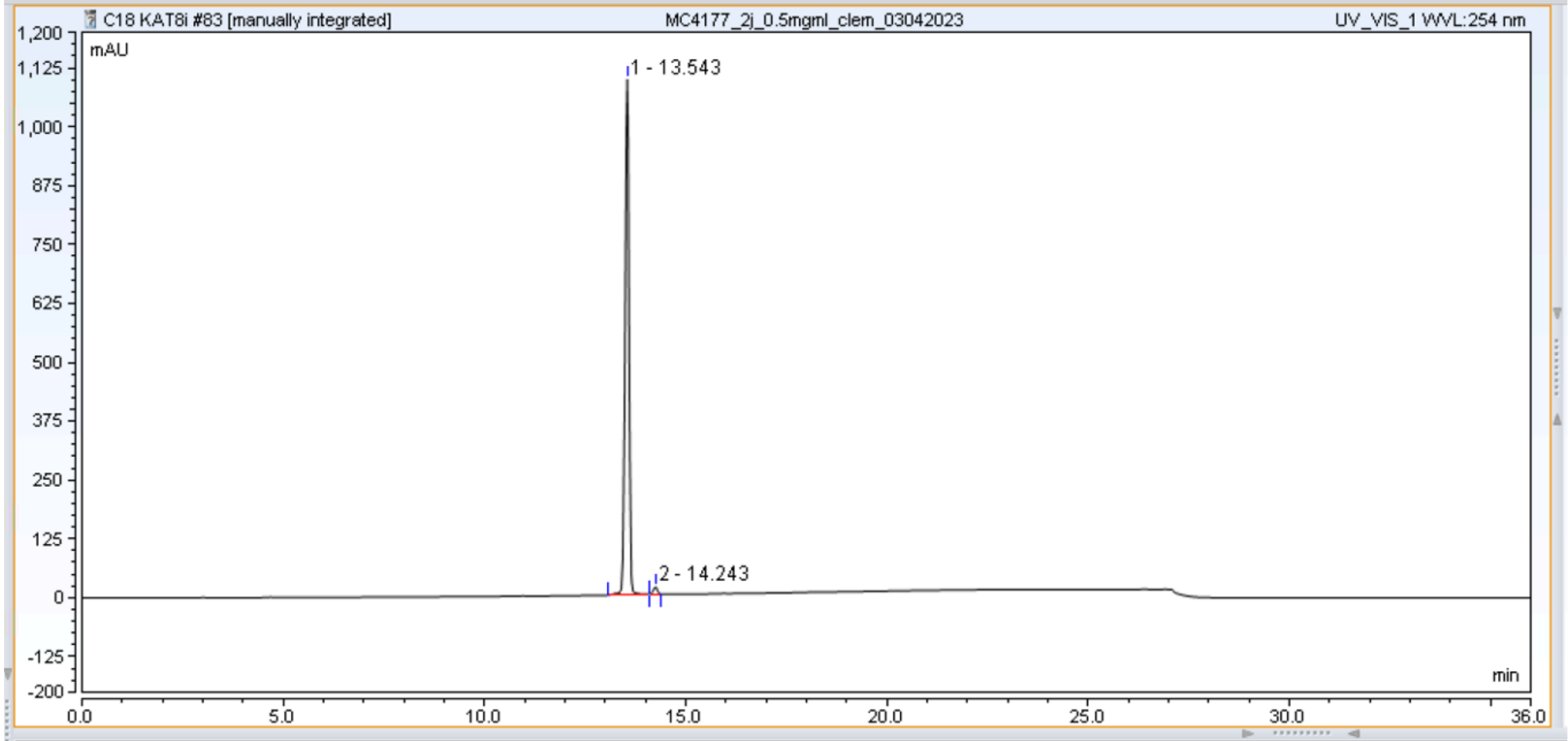

Peak No.	Ret.Time min	Amount n.a.	Rel.Area %	Area mAU*min	Height mAU	Type	Width (50% min	Asym. EP	Resol. EP	Plates EP
1	13.543	n.a.	98.51	124.4290	1094.52	BM *	0.106	0.91	3.85	90784
2	14.243	n.a.	1.49	1.8771	16.17	MB*	0.109	0.87	n.a.	95132
Maximum		0.0000	98.51	124.4290	1094.52		0.109	0.91	3.85	95132
Minimum		0.0000	1.49	1.8771	16.17		0.106	0.87	3.85	90784
Sum		0.0000	100.00	126.3062	1110.69					

2k MC4208

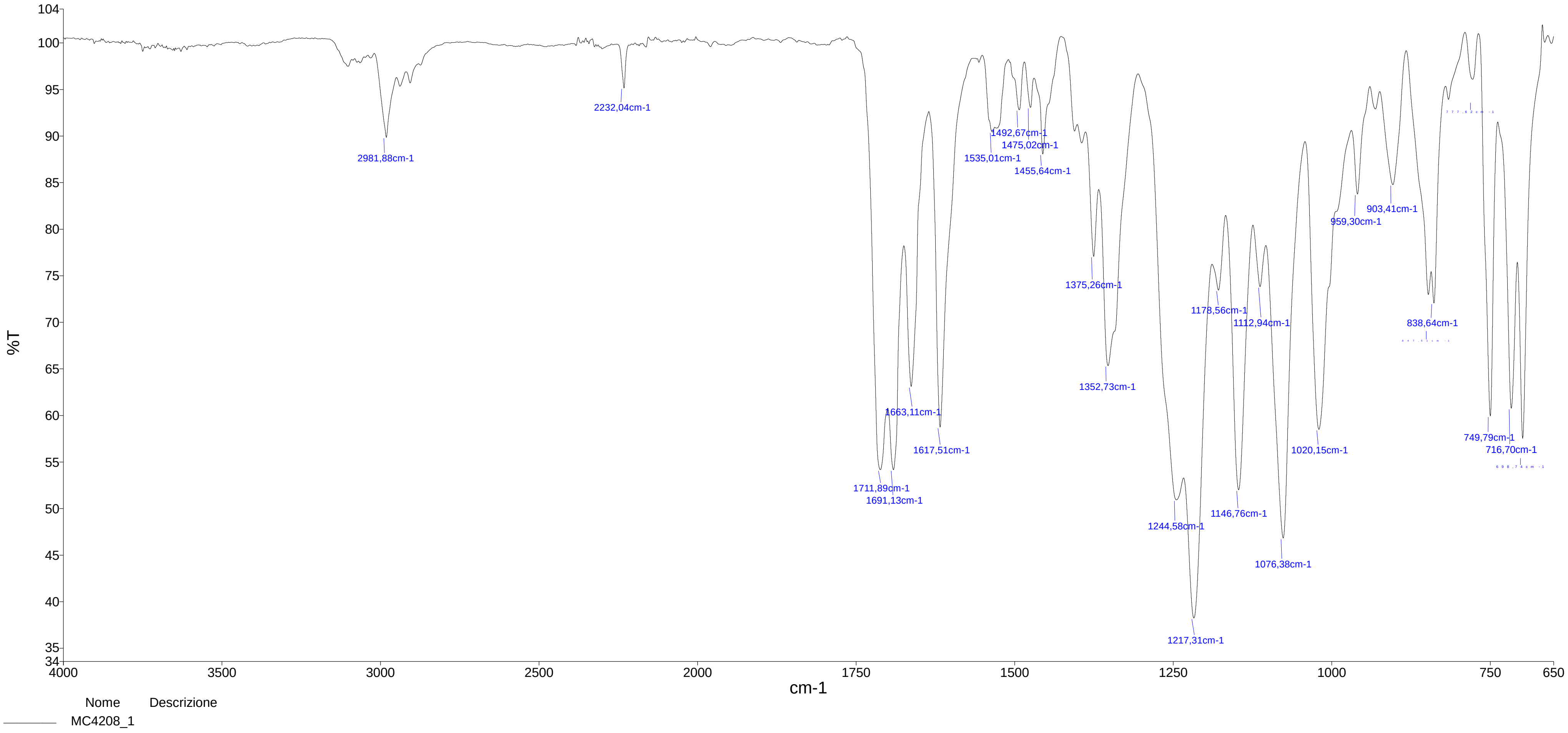

%T
cm-1
2981,88cm-1
2232,04cm-1
1711,89cm-1
1691,13cm-1
1663,11cm-1
1617,51cm-1
1535,01cm-1
1492,67cm-1
1475,02cm-1
1455,64cm-1
1375,26cm-1
1352,73cm-1
1244,58cm-1
1217,31cm-1
1178,56cm-1
1146,76cm-1
1112,94cm-1
1076,38cm-1
1020,15cm-1
959,30cm-1
903,41cm-1
838,64cm-1
749,79cm-1
716,70cm-1
Nome
Descrizione
MC4208_1

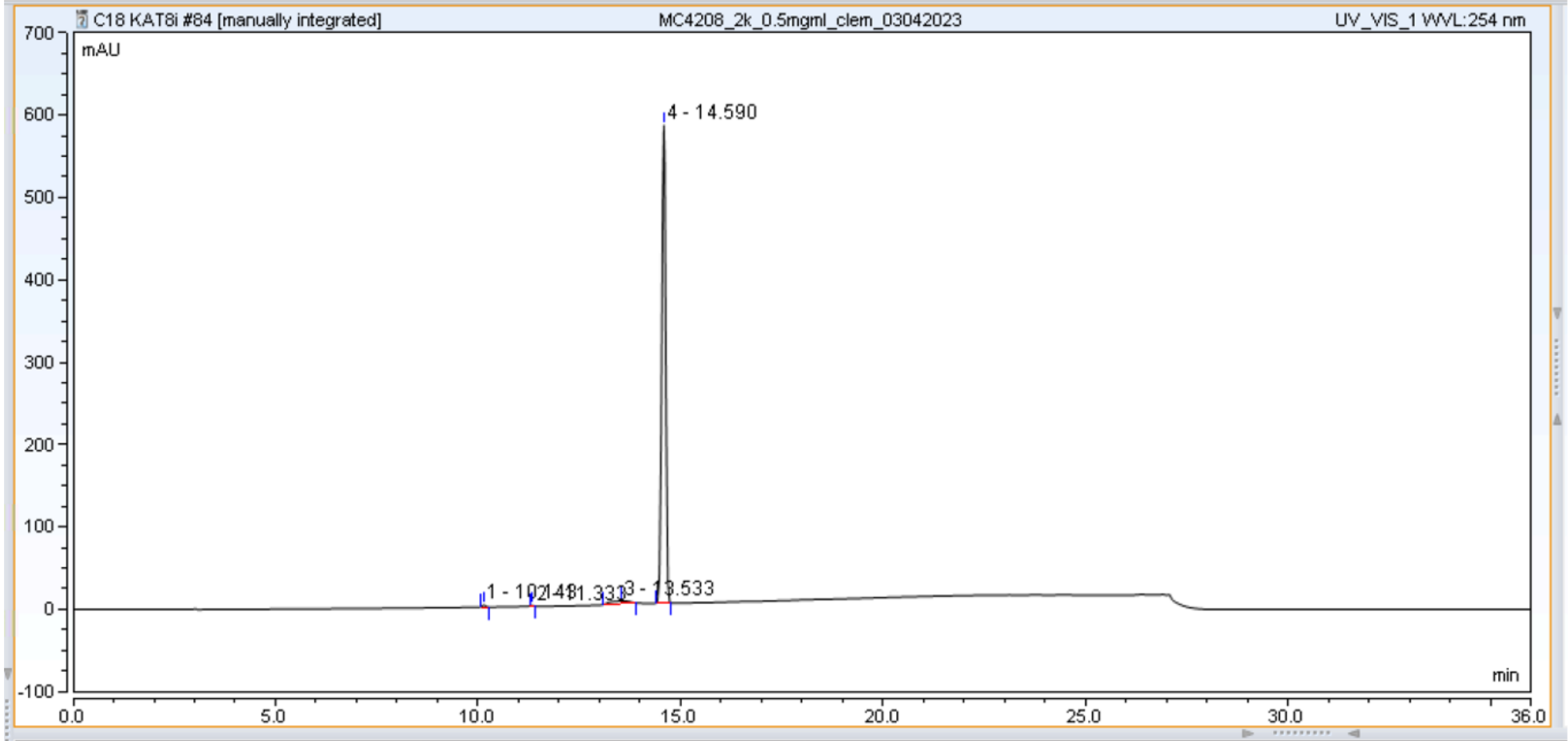

	A	B	C	D	E	F	G	H	I	J	K
1	Peak	Ret.Time	Amount	Rel.Area	Area	Height	Type	Width (50%	Asym.	Resol.	Plates
2	No.	min	n.a.	%	mAU*min	mAU		min	EP	EP	EP
4	1	10.143	n.a.	0.37	0.2459	2.42	BMB*	0.100	1.02	7.80	57444
5	2	11.333	n.a.	0.11	0.0772	0.98	BMB*	0.080	1.10	3.98	109900
6	3	13.533	n.a.	2.68	1.8011	3.39	BMB*	0.573	0.91	1.84	3095
7	4	14.590	n.a.	96.84	65.1264	579.71	BMB*	0.106	0.92	n.a.	104457
8	Maximum		0.0000	96.84	65.1264	579.71		0.573	1.10	7.80	109900
9	Minimum		0.0000	0.11	0.0772	0.98		0.080	0.91	1.84	3095
10	Sum		0.0000	100.00	67.2506	586.51					
11											

2l MC4204

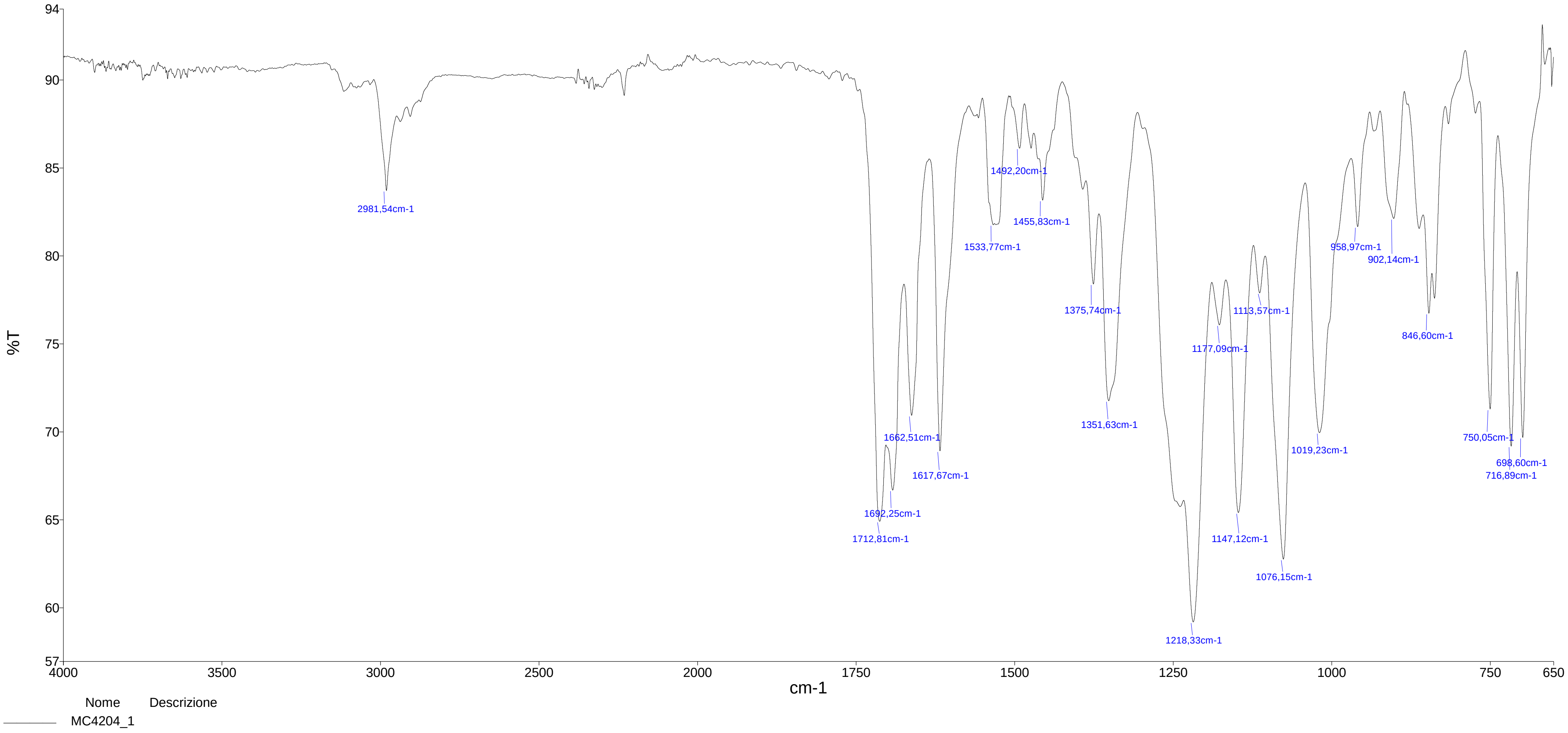
%T
cm-1
2981,54cm-1
1712,81cm-1
1692,25cm-1
1662,51cm-1
1617,67cm-1
1533,77cm-1
1492,20cm-1
1455,83cm-1
1375,74cm-1
1351,63cm-1
1218,33cm-1
1177,09cm-1
1147,12cm-1
1113,57cm-1
1076,15cm-1
1019,23cm-1
958,97cm-1
902,14cm-1
846,60cm-1
750,05cm-1
716,89cm-1
698,60cm-1
Nome Descrizione
MC4204_1

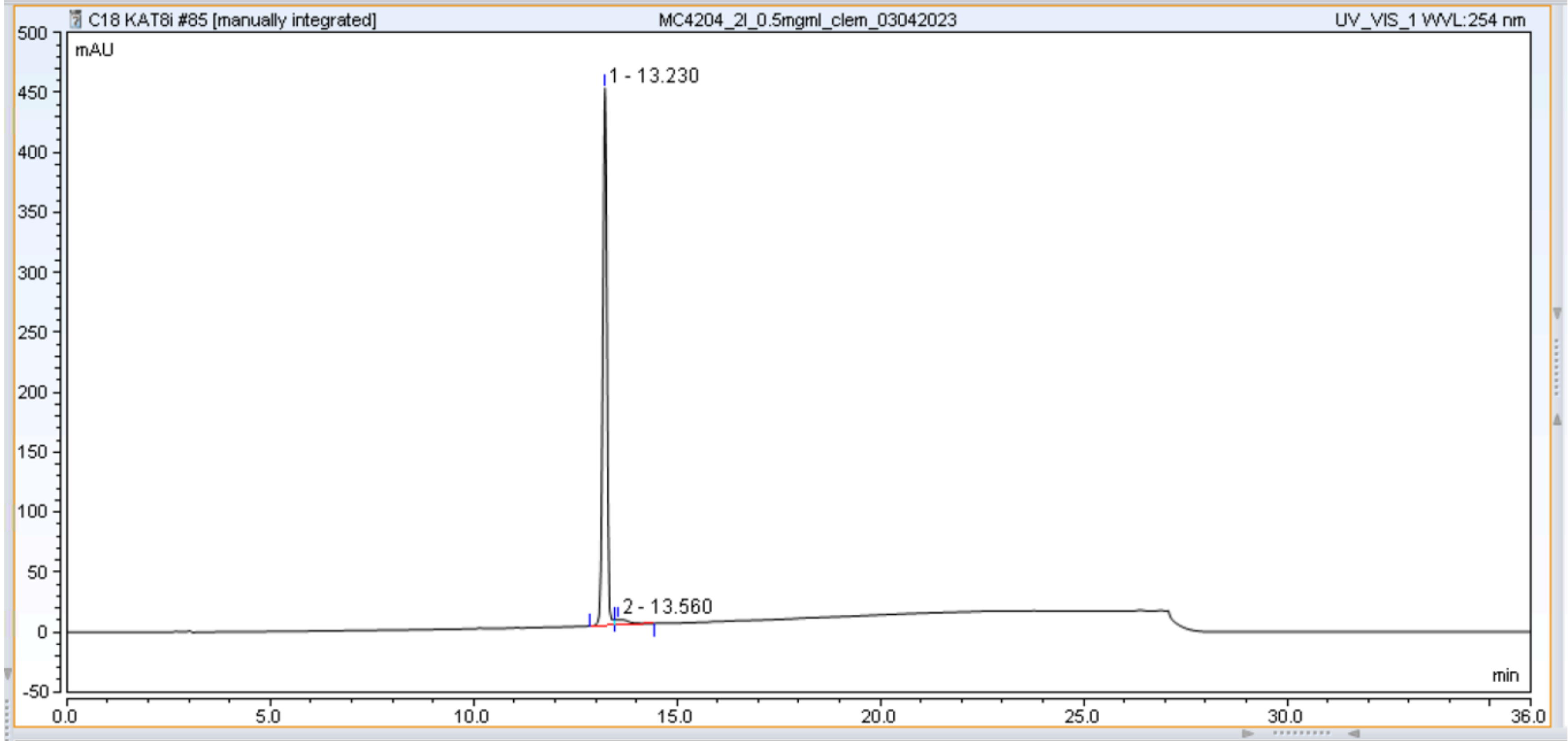

	A	B	C	D	E	F	G	H	I	J	K
1	Peak	Ret.Time	Amount	Rel.Area	Area	Height	Type	Width (50%	Asym.	Resol.	Plates
2	No.	min		%	mAU*min	mAU		min	EP	EP	EP
4	1	13.230	n.a.	96.66	52.0938	448.22	BM	0.108	0.93	n.a.	83258
5	2	13.560	n.a.	3.34	1.8026	4.35	MB	n.a.	n.a.	n.a.	n.a.
6	Maximum		0.0000	96.66	52.0938	448.22		0.108	0.93	0.00	83258
7	Minimum		0.0000	3.34	1.8026	4.35		0.108	0.93	0.00	83258
8	Sum		0.0000	100.00	53.8964	452.57					
9											

2m MC4188

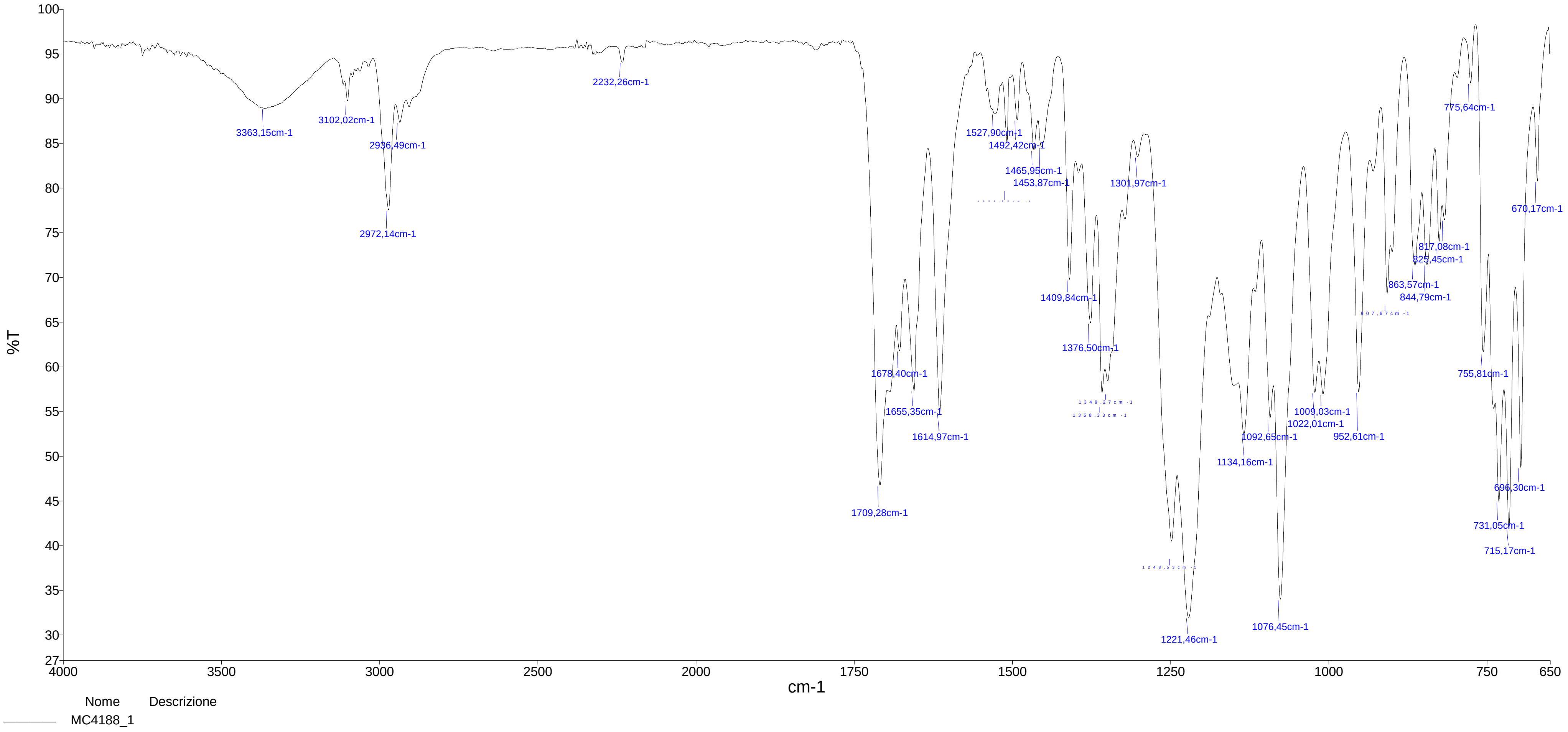
%T
cm-1
3363,15cm-1
3102,02cm-1
2936,49cm-1
2972,14cm-1
2232,26cm-1
1709,28cm-1
1678,40cm-1
1655,35cm-1
1614,97cm-1
1527,90cm-1
1492,42cm-1
1465,95cm-1
1453,87cm-1
1409,84cm-1
1376,50cm-1
1301,97cm-1
1221,46cm-1
1134,16cm-1
1092,65cm-1
1076,45cm-1
1022,01cm-1
1009,03cm-1
952,61cm-1
863,57cm-1
844,79cm-1
825,45cm-1
817,08cm-1
775,64cm-1
755,81cm-1
731,05cm-1
715,17cm-1
696,30cm-1
670,17cm-1
Nome Descrizione
MC4188_1

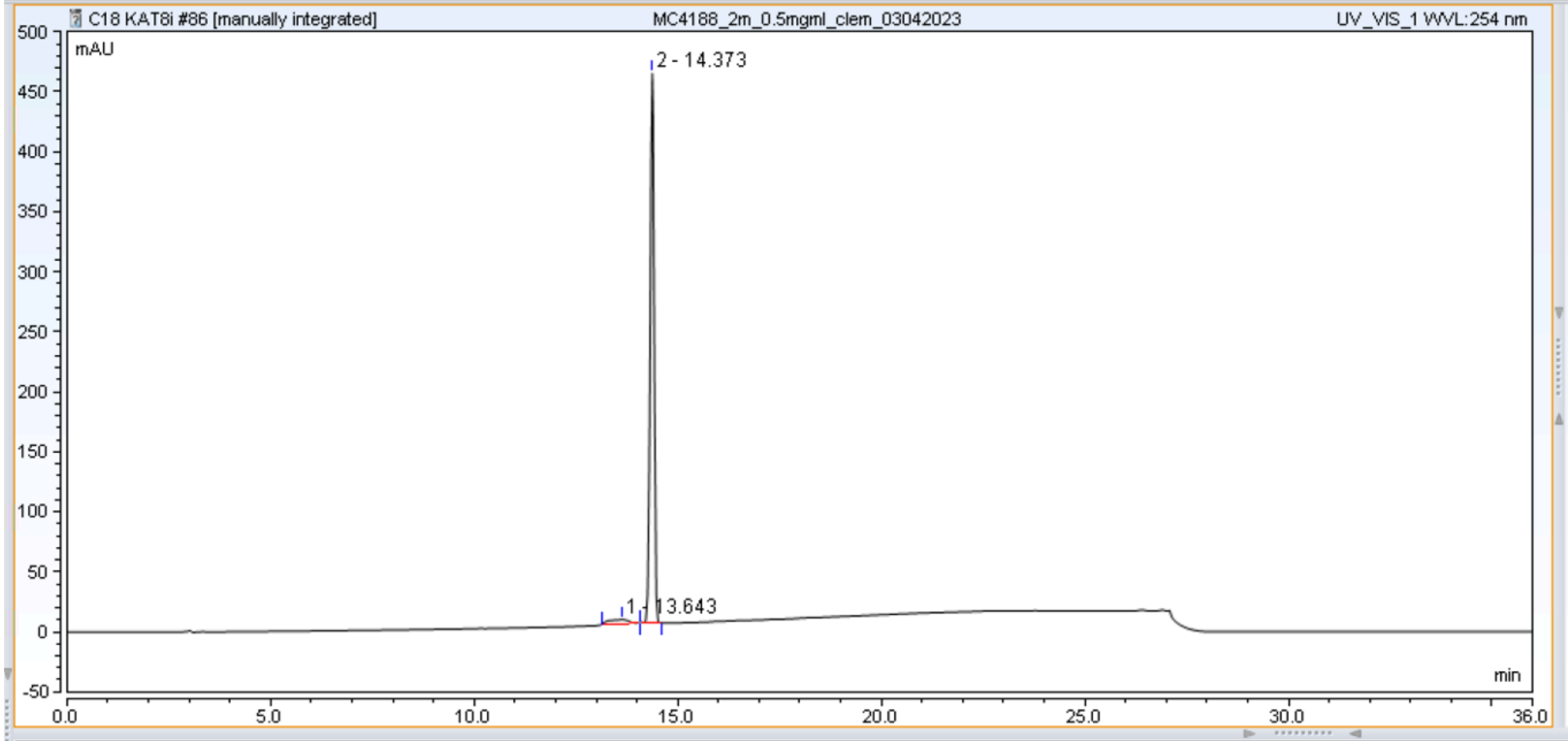

	A	B	C	D	E	F	G	H	I	J	K
1	Peak	Ret.Time	Amount	Rel.Area	Area	Height	Type	Width (50%	Asym.	Resol.	Plates
2	No.	min	n.a.	%	mAU*min	mAU		min	EP	EP	EP
4	1	13.643	n.a.	3.96	2.1446	3.42	BM *	0.617	n.a.	1.19	2712
5	2	14.373	n.a.	96.04	52.0605	458.00	MB*	0.107	0.92	n.a.	99766
6	Maximum		0.0000	96.04	52.0605	458.00		0.617	0.92	1.19	99766
7	Minimum		0.0000	3.96	2.1446	3.42		0.107	0.92	1.19	2712
8	Sum		0.0000	100.00	54.2051	461.42					
9											

2n MC4186

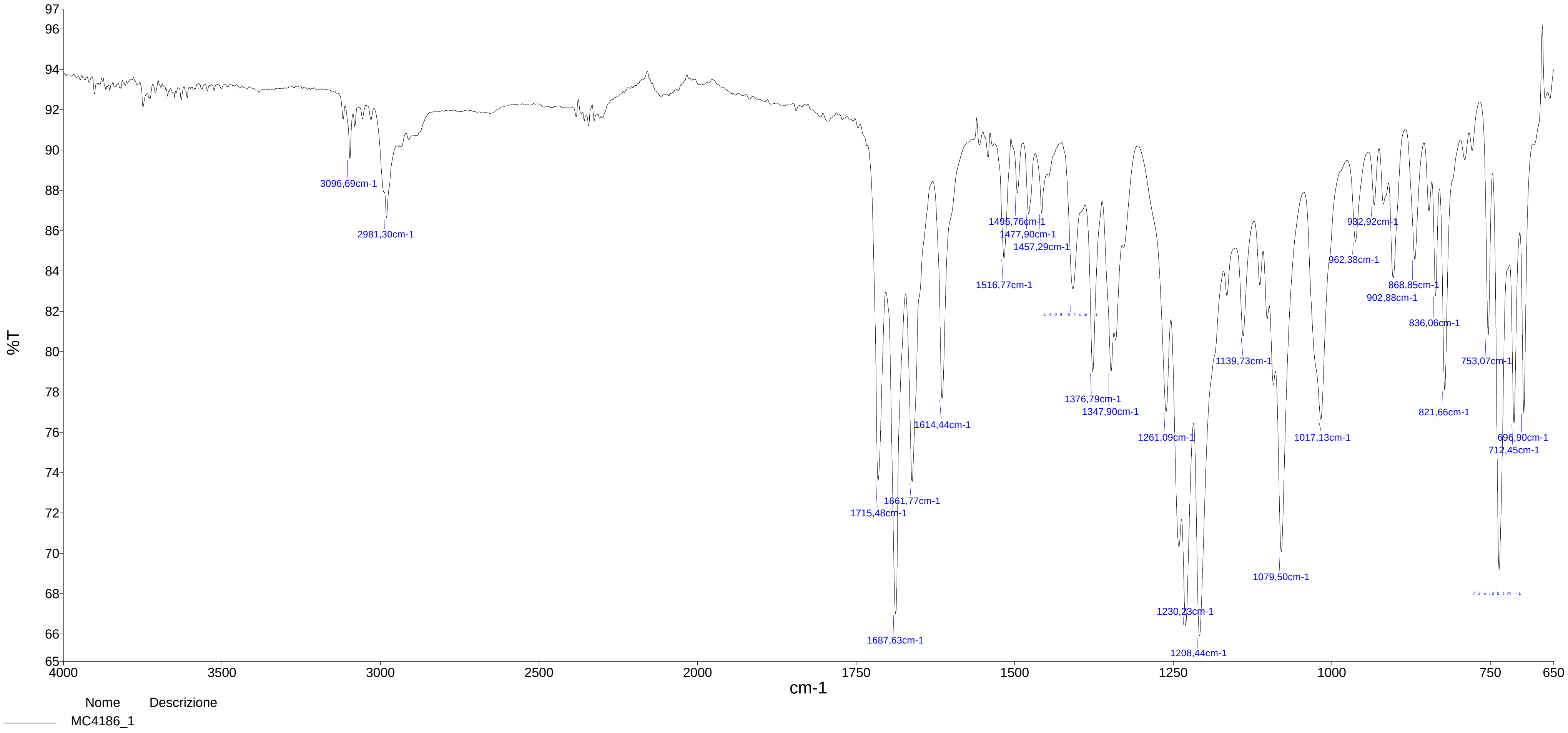

%T
cm-1
3096,69cm-1
2981,30cm-1
1715,48cm-1
1687,63cm-1
1661,77cm-1
1614,44cm-1
1516,77cm-1
1495,76cm-1
1477,90cm-1
1457,29cm-1
1408,04cm-1
1376,79cm-1
1347,90cm-1
1261,09cm-1
1230,23cm-1
1208,44cm-1
1139,73cm-1
1079,50cm-1
1017,13cm-1
962,38cm-1
932,92cm-1
902,88cm-1
868,85cm-1
836,06cm-1
821,66cm-1
753,07cm-1
735,98cm-1
712,45cm-1
696,90cm-1
Nome Descrizione
MC4186_1

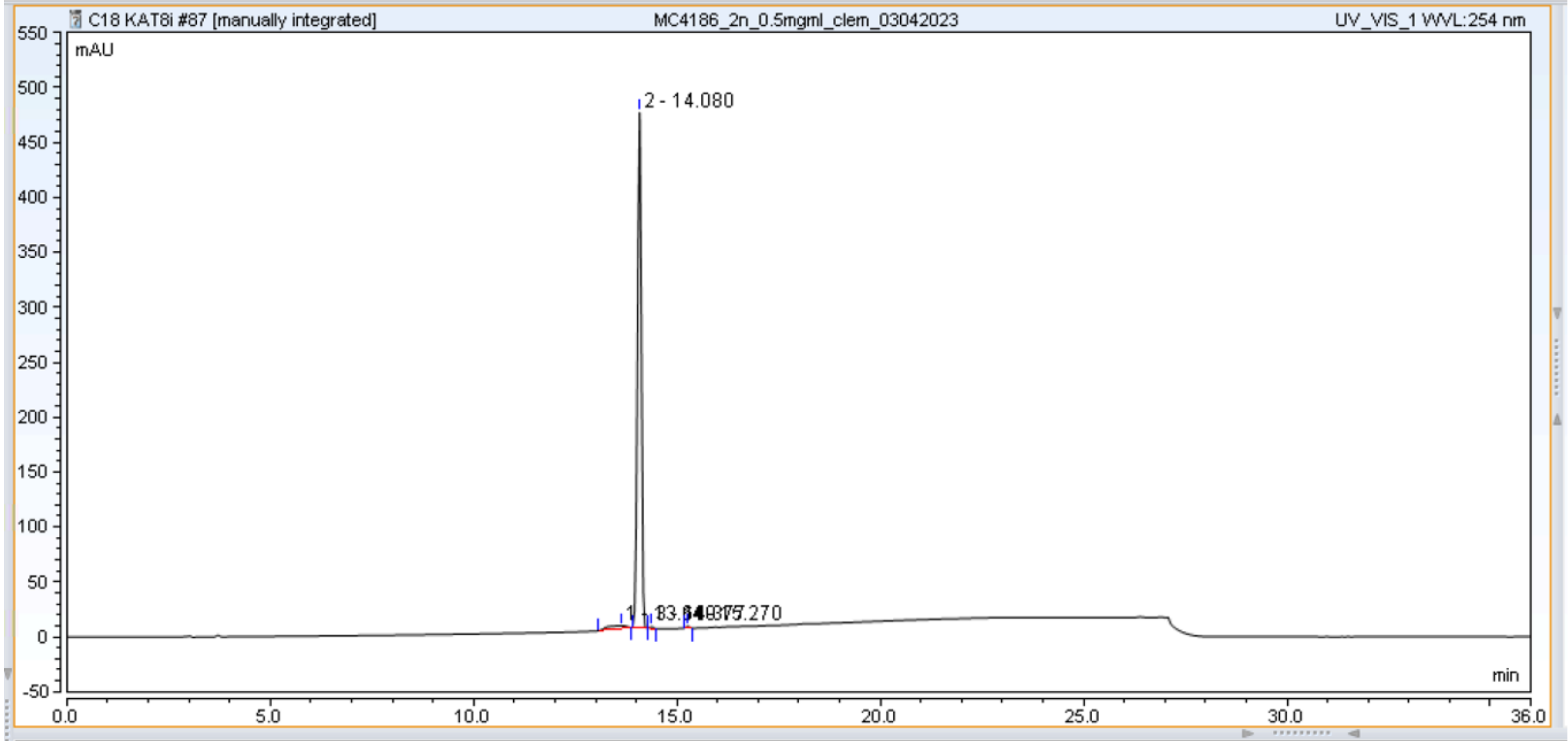

	A	B	C	D	E	F	G	H	I	J	K
1	Peak	Ret.Time	Amount	Rel.Area	Area	Height	Type	Width (50%	Asym.	Resol.	Plates
2	No.	min	n.a.	%	mAU*min	mAU		min	EP	EP	EP
4	1	13.640	n.a.	3.02	1.6319	2.56	BMB*	0.597	0.71	0.74	2892
5	2	14.080	n.a.	96.36	52.0346	469.17	BMB*	0.105	0.92	1.74	100569
6	3	14.377	n.a.	0.33	0.1798	1.83	BMB*	0.096	1.00	5.58	123520
7	4	15.270	n.a.	0.29	0.1545	1.66	BMB*	0.093	1.03	n.a.	150522
8	Maximum		0.0000	96.36	52.0346	469.17		0.597	1.03	5.58	150522
9	Minimum		0.0000	0.29	0.1545	1.66		0.093	0.71	0.74	2892
10	Sum		0.0000	100.00	54.0007	475.21					
11											

3a MC4164

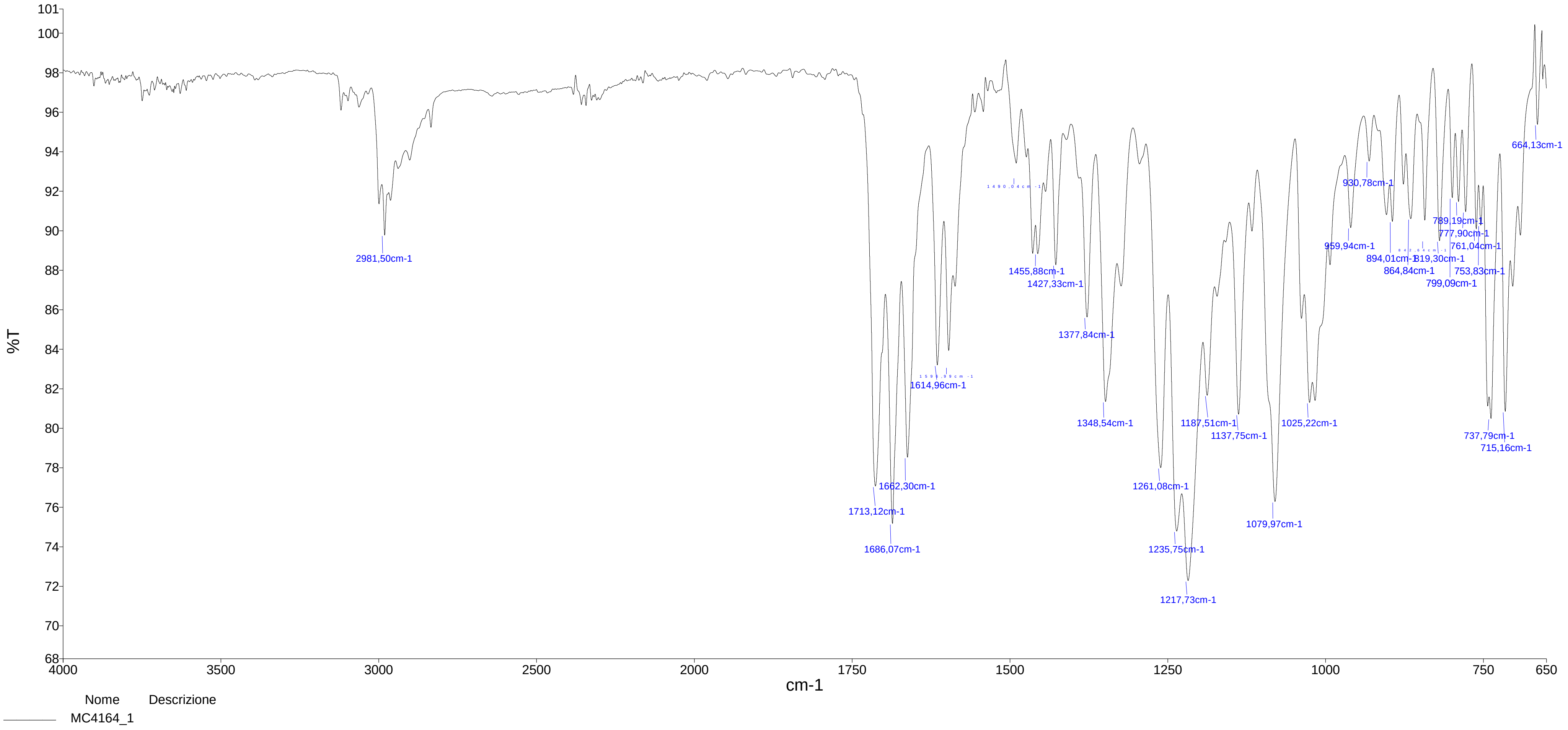

%T
cm-1
2981,50cm-1
1713,12cm-1
1686,07cm-1
1662,30cm-1
1614,96cm-1
1490,04cm-1
1455,88cm-1
1427,33cm-1
1377,84cm-1
1348,54cm-1
1261,08cm-1
1235,75cm-1
1217,73cm-1
1187,51cm-1
1137,75cm-1
1079,97cm-1
1025,22cm-1
959,94cm-1
930,78cm-1
894,01cm-1
864,84cm-1
819,30cm-1
799,09cm-1
789,19cm-1
777,90cm-1
761,04cm-1
753,83cm-1
737,79cm-1
715,16cm-1
664,13cm-1
Nome
Descrizione
MC4164_1

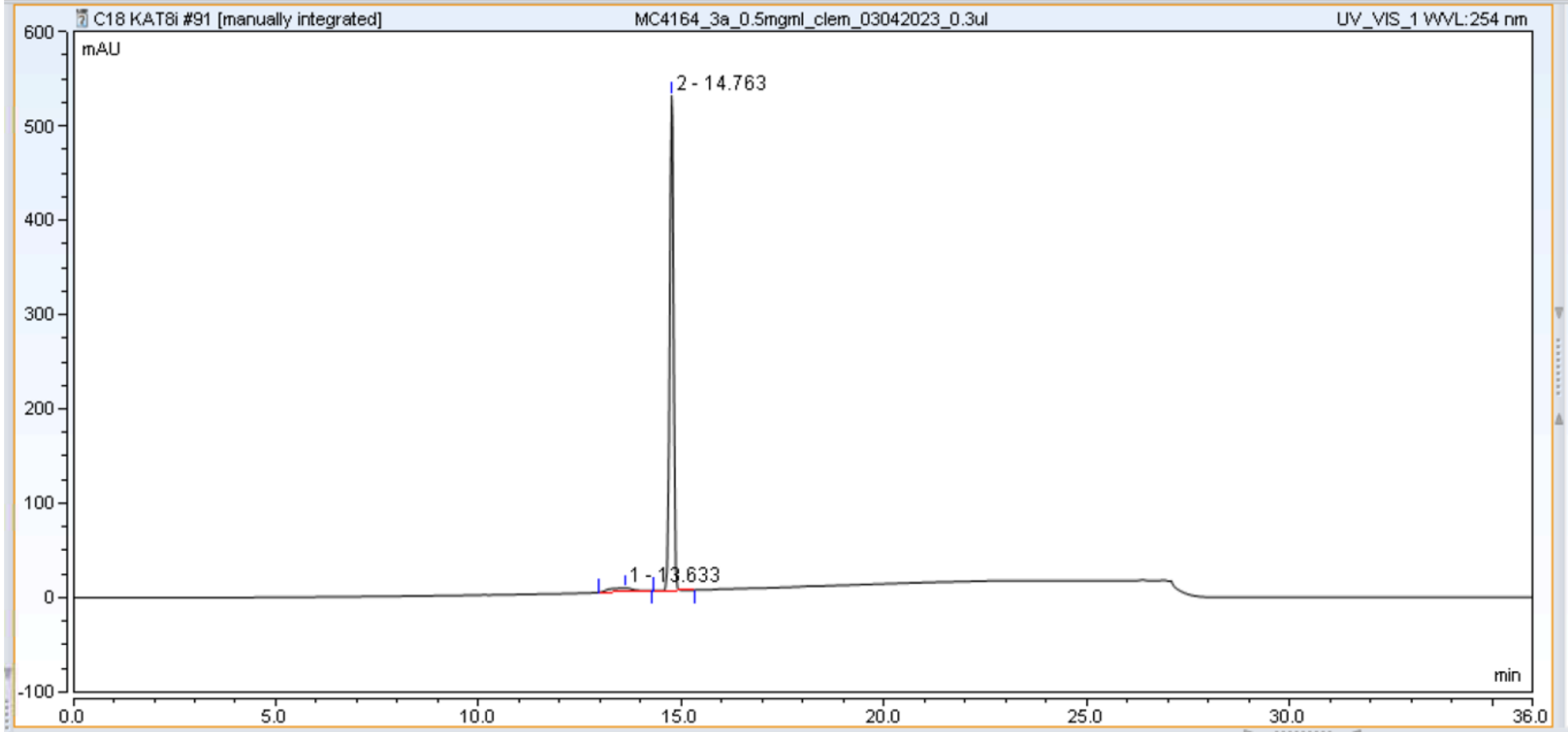

	A	B	C	D	E	F	G	H	I	J	K
1	Peak	Ret.Time	Amount	Rel.Area	Area	Height	Type	Width (50%	Asym.	Resol.	Plates
2	No.	min	n.a.	%	mAU*min	mAU		min	EP	EP	EP
4	1	13.633	n.a.	4.58	2.7398	3.79	BMB*	0.690	0.92	1.68	2163
5	2	14.763	n.a.	95.42	57.0931	524.87	BMB*	0.102	0.94	n.a.	116027
6	Maximum		0.0000	95.42	57.0931	524.87		0.690	0.94	1.68	116027
7	Minimum		0.0000	4.58	2.7398	3.79		0.102	0.92	1.68	2163
8	Sum		0.0000	100.00	59.8328	528.66					
9											

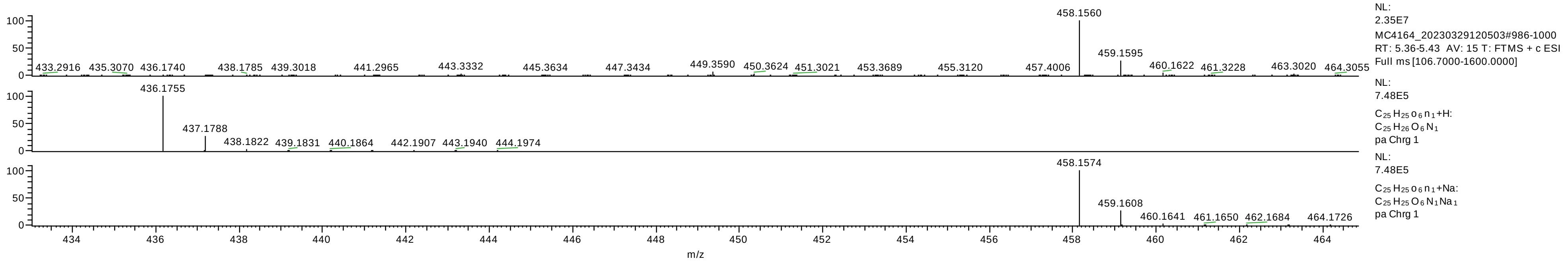
NL:
2.35E7
MC4164_20230329120503#986-1000
RT: 5.36-5.43 AV: 15 T: FTMS + c ESI
Full ms [106.7000-1600.0000]
NL:
7.48E5
C25 H25 o6 n1+H:
C25 H26 O6 N1
pa Chrg 1
NL:
7.48E5
C25 H25 o6 n1+Na:
C25 H25 O6 N1 Na1
pa Chrg 1
458.1560
459.1595
436.1755
437.1788
458.1574
459.1608
m/z

3b MC4182

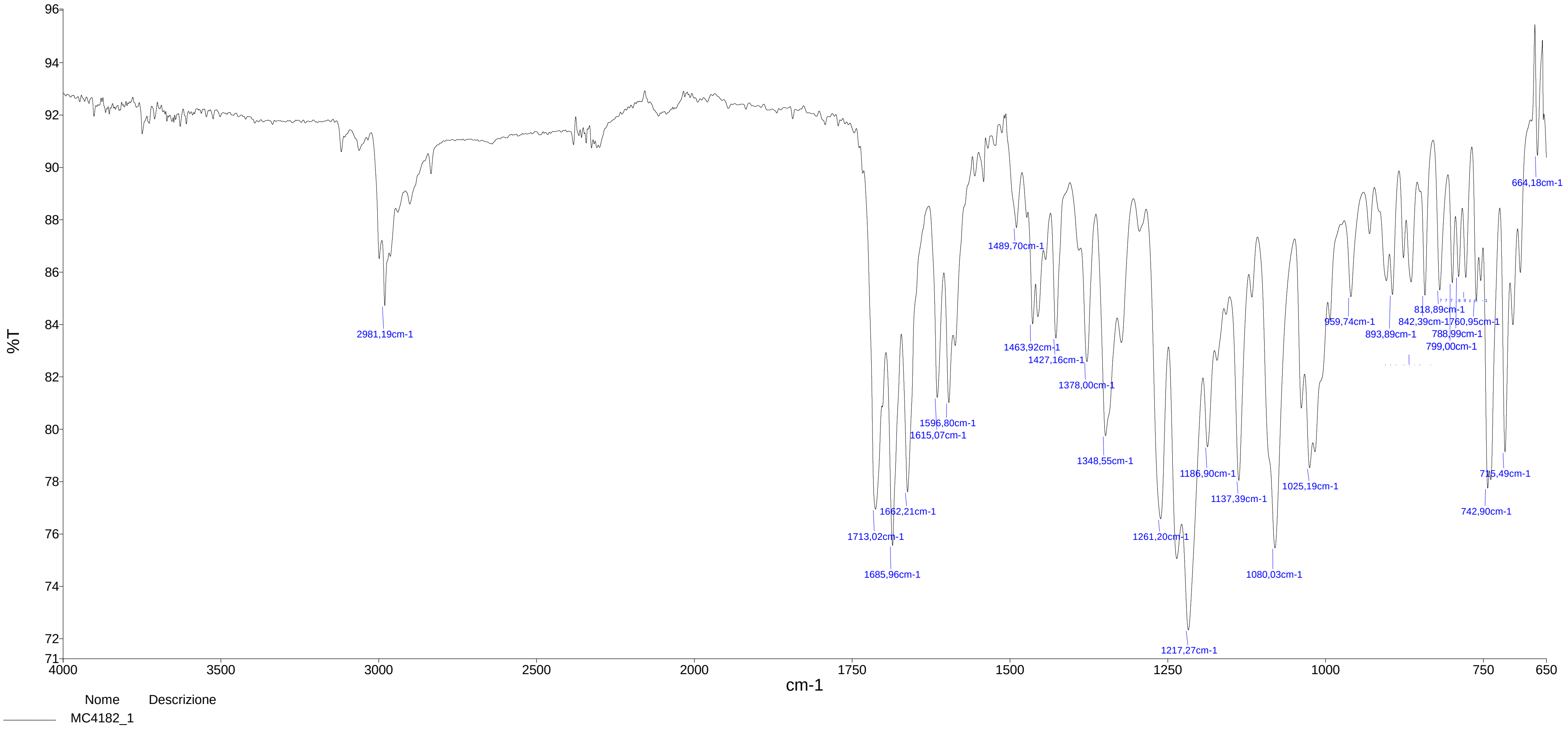
%T
cm-1
2981,19cm-1
1489,70cm-1
1463,92cm-1
1427,16cm-1
1378,00cm-1
1348,55cm-1
1596,80cm-1
1615,07cm-1
1713,02cm-1
1685,96cm-1
1662,21cm-1
1261,20cm-1
1217,27cm-1
1186,90cm-1
1137,39cm-1
1080,03cm-1
1025,19cm-1
959,74cm-1
893,89cm-1
842,39cm-1
818,89cm-1
760,95cm-1
788,99cm-1
799,00cm-1
742,90cm-1
715,49cm-1
664,18cm-1
Nome Descrizione
MC4182_1

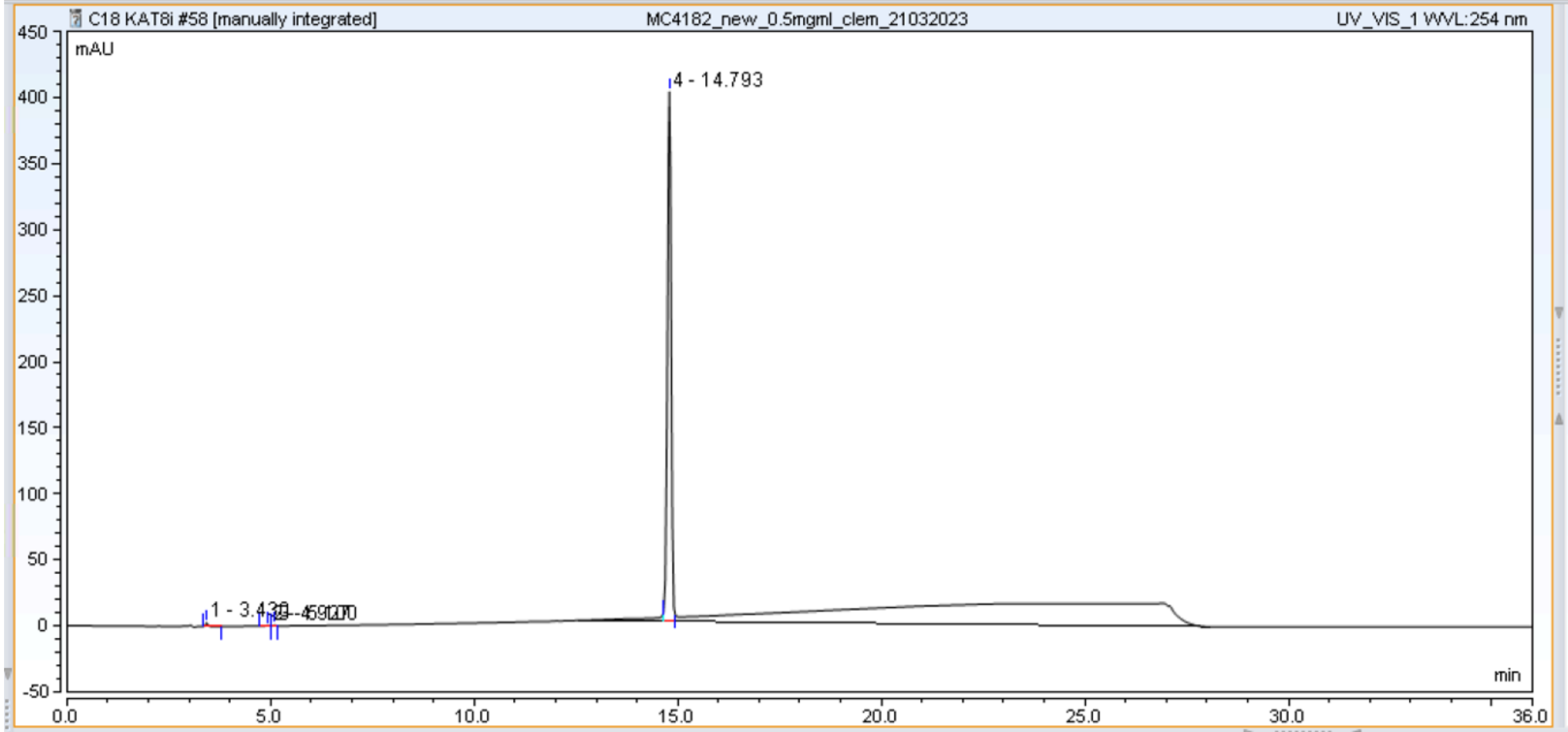

	A	B	C	D	E	F	G	H	I	J	K
1	Peak	Ret.Time	Amount	Rel.Area	Area	Height	Type	Width (50%	Asym.	Resol.	Plates
2	No.	min	µl	%	mAU*min	mAU		min	EP	EP	EP
4	1	3.430	n.a.	0.40	0.1731	3.09	BMB*	0.054	0.99	14.16	22415
5	2	4.927	n.a.	0.12	0.0496	0.65	BM *	0.071	0.90	1.46	26827
6	3	5.100	n.a.	0.10	0.0436	0.61	MB*	0.070	0.94	68.12	29613
7	4	14.793	n.a.	99.38	42.5068	401.33	M *	0.098	0.93	n.a.	125825
8	Maximum		0.0000	99.38	42.5068	401.33		0.098	0.99	68.12	125825
9	Minimum		0.0000	0.10	0.0436	0.61		0.054	0.90	1.46	22415
10	Sum		0.0000	100.00	42.7731	405.68					
11											

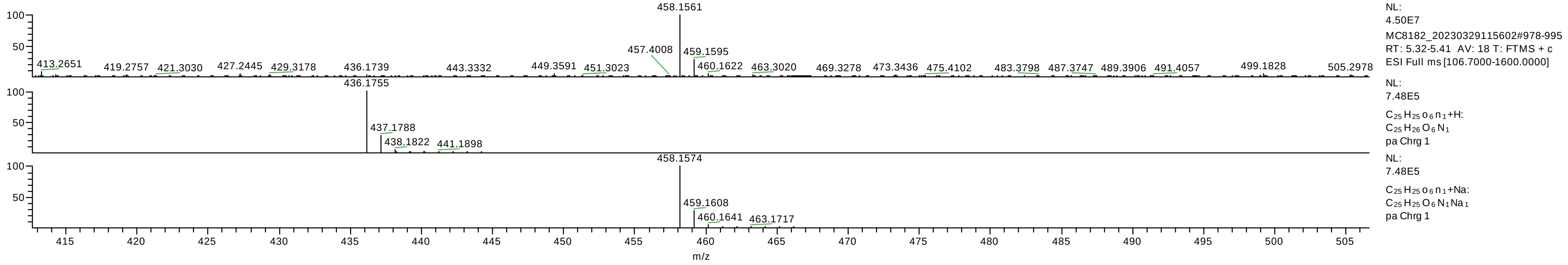

458.1561
NL:
4.50E7
MC8182_20230329115602#978-995
RT: 5.32-5.41 AV: 18 T: FTMS + c
ESI Full ms [106.7000-1600.0000]
413.2651
419.2757
421.3030
427.2445
429.3178
436.1739
443.3332
449.3591
451.3023
457.4008
459.1595
460.1622
463.3020
469.3278
473.3436
475.4102
483.3798
487.3747
489.3906
491.4057
499.1828
505.2978
436.1755
NL:
7.48E5
C25 H25 o6 n1+H:
C25 H26 O6 N1
pa Chrg 1
437.1788
438.1822
441.1898
458.1574
NL:
7.48E5
C25 H25 o6 n1+Na:
C25 H25 O6 N1 Na1
pa Chrg 1
459.1608
460.1641
463.1717
m/z

3c MC4175

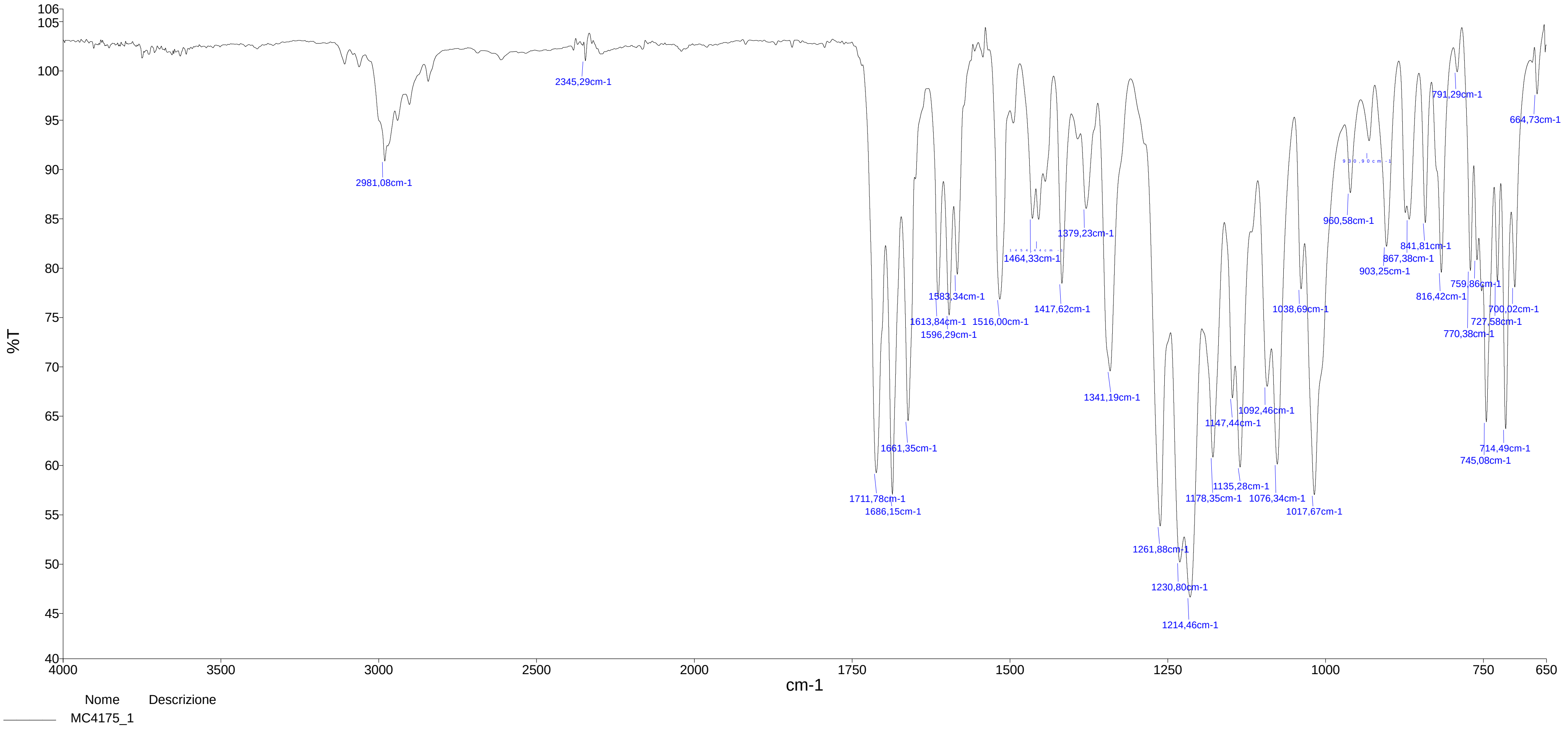

%T
cm-1
2981,08cm-1
2345,29cm-1
1711,78cm-1
1686,15cm-1
1661,35cm-1
1613,84cm-1
1596,29cm-1
1583,34cm-1
1516,00cm-1
1464,33cm-1
1417,62cm-1
1379,23cm-1
1341,19cm-1
1261,88cm-1
1230,80cm-1
1214,46cm-1
1178,35cm-1
1147,44cm-1
1135,28cm-1
1092,46cm-1
1076,34cm-1
1038,69cm-1
1017,67cm-1
960,58cm-1
903,25cm-1
867,38cm-1
841,81cm-1
816,42cm-1
791,29cm-1
770,38cm-1
759,86cm-1
745,08cm-1
727,58cm-1
714,49cm-1
700,02cm-1
664,73cm-1
Nome Descrizione
MC4175_1

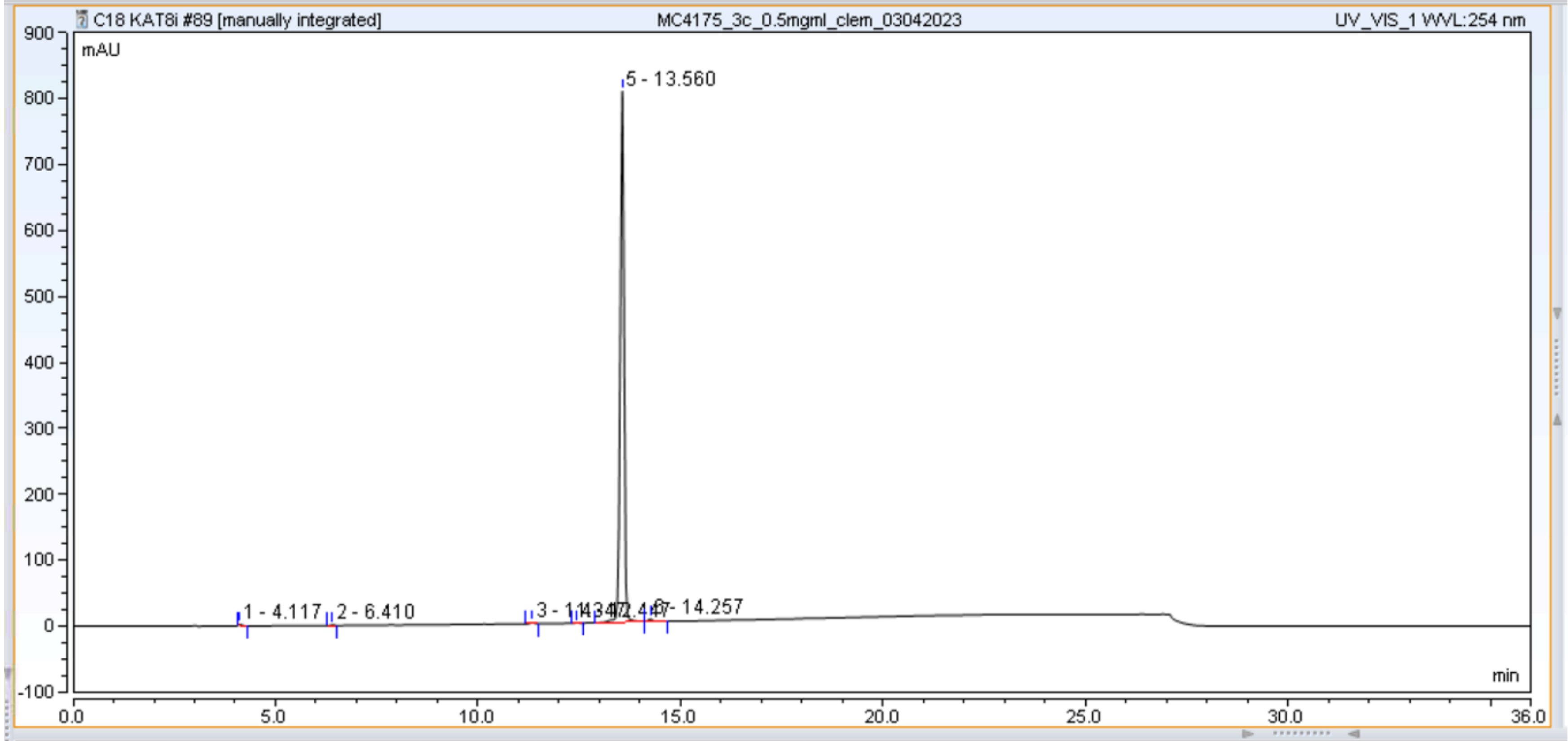

	A	B	C	D	E	F	G	H	I	J	K
1	Peak	Ret.Time	Amount	Rel.Area	Area	Height	Type	Width (50%	Asym.	Resol.	Plates
2	No.	min	µl	%	mAU*min	mAU		min	EP	EP	EP
4	1	4.117	n.a.	0.14	0.1257	1.85	BMB*	0.068	1.63	17.50	20052
5	2	6.410	n.a.	0.11	0.1000	1.09	BMB	0.086	0.96	30.88	30654
6	3	11.347	n.a.	0.24	0.2192	2.00	BMB	0.102	0.89	6.35	67932
7	4	12.447	n.a.	0.07	0.0637	0.60	BMB*	0.102	0.90	6.45	82676
8	5	13.560	n.a.	98.81	89.8827	805.24	BM	0.102	0.93	3.90	98028
9	6	14.257	n.a.	0.63	0.5746	4.39	MB	0.109	n.a.	n.a.	95469
10	Maximum		0.0000	98.81	89.8827	805.24		0.109	1.63	30.88	98028
11	Minimum		0.0000	0.07	0.0637	0.60		0.068	0.89	3.90	20052
12	Sum		0.0000	100.00	90.9661	815.17					
13											

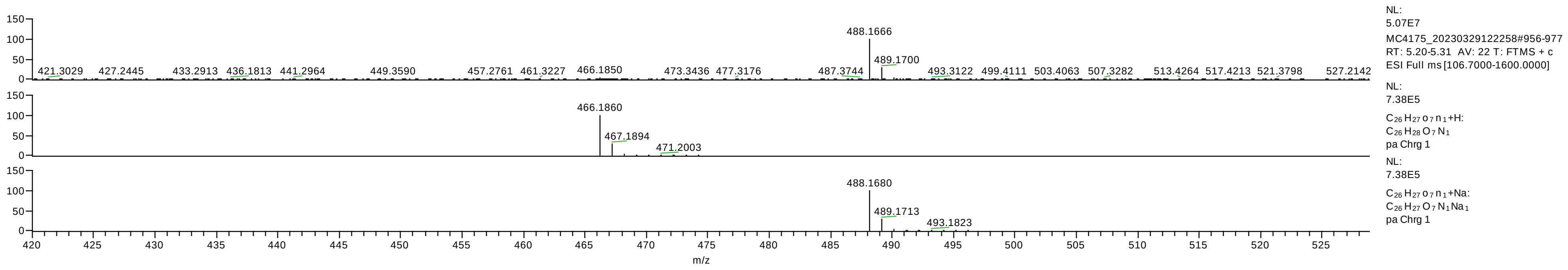
NL: 5.07E7
MC4175_20230329122258#956-977
RT: 5.20-5.31 AV: 22 T: FTMS + c
ESI Full ms [106.7000-1600.0000]
NL: 7.38E5
C26 H27 o7 n1 +H:
C26 H28 O7 N1
pa Chrg 1
NL: 7.38E5
C26 H27 o7 n1 +Na:
C26 H27 O7 N1 Na1
pa Chrg 1
488.1666
466.1860
488.1680
m/z

3d MC4176

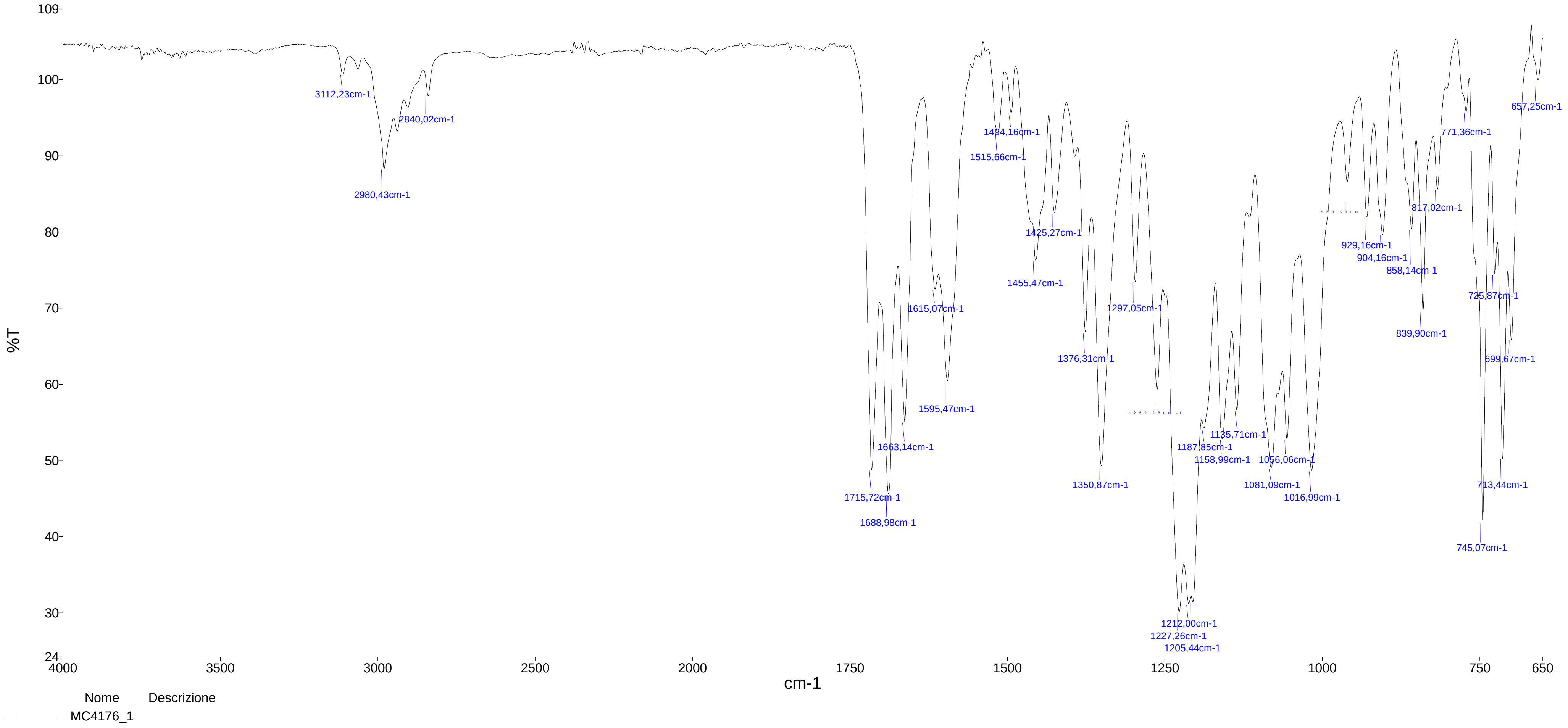

109
100
90
80
70
60
50
40
30
24
%T
4000
3500
3000
2500
2000
1750
1500
1250
1000
750
650
cm-1
3112,23cm-1
2980,43cm-1
2840,02cm-1
1715,72cm-1
1688,98cm-1
1663,14cm-1
1615,07cm-1
1595,47cm-1
1515,66cm-1
1494,16cm-1
1455,47cm-1
1425,27cm-1
1376,31cm-1
1350,87cm-1
1297,05cm-1
1227,26cm-1
1212,00cm-1
1205,44cm-1
1187,85cm-1
1158,99cm-1
1135,71cm-1
1081,09cm-1
1056,06cm-1
1016,99cm-1
929,16cm-1
904,16cm-1
858,14cm-1
839,90cm-1
817,02cm-1
771,36cm-1
745,07cm-1
725,87cm-1
713,44cm-1
699,67cm-1
657,25cm-1
Nome
Descrizione
MC4176_1

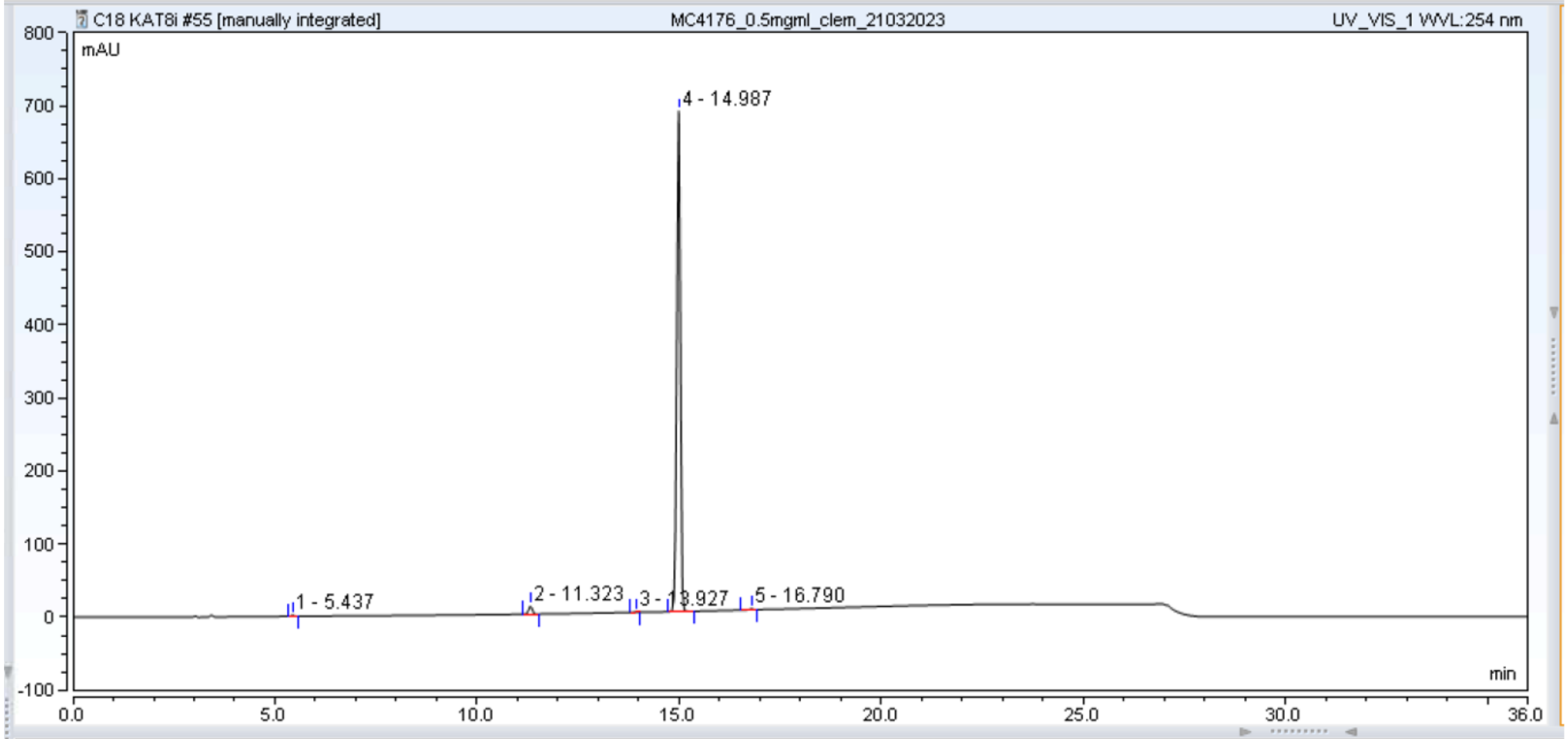

	A	B	C	D	E	F	G	H	I	J	K
1	Peak	Ret.Time	Amount	Rel.Area	Area	Height	Type	Width (50%	Asym.	Resol.	Plates
2	No.	min	n.a.	%	mAU*min	mAU		min	EP	EP	EP
4	1	5.437	n.a.	0.17	0.1215	1.48	BMB	0.076	0.96	40.30	28036
5	2	11.323	n.a.	1.62	1.1677	11.38	BMB	0.096	0.94	16.35	77188
6	3	13.927	n.a.	0.08	0.0543	0.57	BMB	0.092	0.93	6.64	126928
7	4	14.987	n.a.	97.91	70.7309	684.85	BMB	0.096	0.94	10.96	134149
8	5	16.790	n.a.	0.23	0.1654	1.53	BMB	0.098	0.88	n.a.	163418
9	Maximum		0.0000	97.91	70.7309	684.85		0.098	0.96	40.30	163418
10	Minimum		0.0000	0.08	0.0543	0.57		0.076	0.88	6.64	28036
11	Sum		0.0000	100.00	72.2398	699.80					
12											

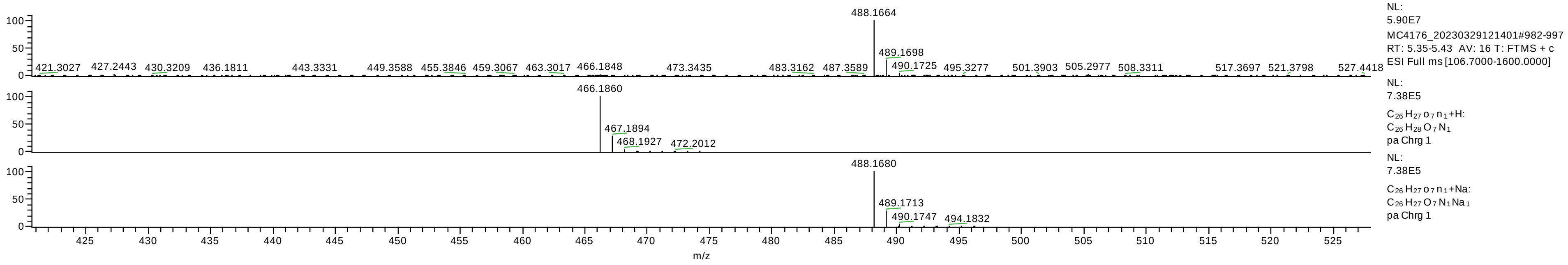
NL:
5.90E7
MC4176_20230329121401#982-997
RT: 5.35-5.43 AV: 16 T: FTMS + c
ESI Full ms [106.7000-1600.0000]
488.1664
489.1698
490.1725
NL:
7.38E5
C26 H27 o7 n1 +H:
C26 H28 O7 N1
pa Chrg 1
466.1860
467.1894
468.1927
472.2012
NL:
7.38E5
C26 H27 o7 n1 +Na:
C26 H27 O7 N1 Na1
pa Chrg 1
488.1680
489.1713
490.1747
494.1832
m/z

Table S2. Elemental analysis of final compounds 2a-n and 3a-d

LAB	CMP	Elemental analysis							
		Calculated				found			
		C	H	N	O	C	H	N	O
**MC4199**	**2a**	70.05	7.10	3.40	19.44	69.92	7.18	3.39	19.48
**MC4158**	**2b**	68.08	5.24	3.31	18.89	68.13	5.27	3.26	18.94
**MC4168**	**2c**	68.08	5.24	3.31	18.89	68.01	5.19	3.37	18.95
**MC4172**	**2d**	65.53	5.04	3.18	18.19	65.45	5.10	3.13	18.22
**MC4166**	**2e**	65.53	5.04	3.18	18.19	65.55	4.98	3.20	18.16
**MC4181**	**2f**	59.52	4.58	2.89	16.52	59.47	4.64	2.84	16.59
**MC4185**	**2g**	59.52	4.58	2.89	16.52	59.49	4.56	2.86	16.48
**MC4165**	**2h**	64.00	4.92	6.22	24.86	64.05	4.98	6.17	24.79
**MC4167**	**2i**	64.00	4.92	6.22	24.86	63.97	4.88	6.26	24.93
**MC4177**	**2j**	69.76	5.15	6.51	18.58	69.71	5.18	6.48	18.65
**MC4208**	**12k**	71.58	6.01	3.34	19.07	71.63	6.07	3.29	19.10
**MC4204**	**2l**	66.83	5.35	3.54	24.28	66.88	5.33	3.59	24.21
**MC4188**	**2m**	64.22	5.14	3.40	19.44	64.17	5.19	3.35	19.50
**MC4186**	**2n**	64.22	5.14	3.40	19.44	64.26	5.11	3.42	19.39
**MC4175**	**3a**	67.09	5.85	3.01	24.06	67.13	5.89	2.94	24.11
**MC4176**	**3b**	67.09	5.85	3.01	24.06	67.02	5.91	3.08	24.12
**MC4164**	**3c**	68.95	5.79	3.22	22.04	68.99	5.83	3.19	22.08
**MC4182**	**3d**	68.95	5.79	3.22	22.04	69.03	5.72	3.27	21.99

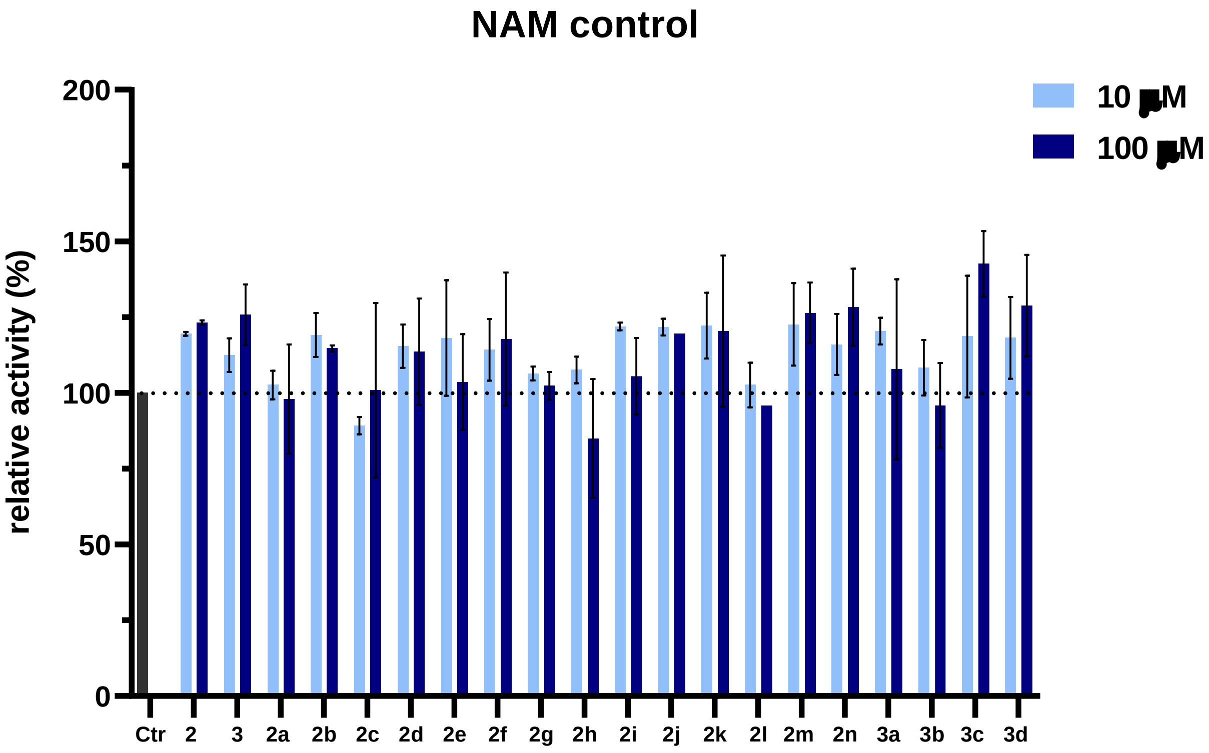

Figure S1. Control experiments for potential compound effects on the coupled enzymes of the assay system. The conversion of 50 µM NAM, the product of the Sirtuin-catalyzed reaction, by the coupled enzymes was tested in presence of DMSO (reference) and 10 µM or 100 µM of each compound.

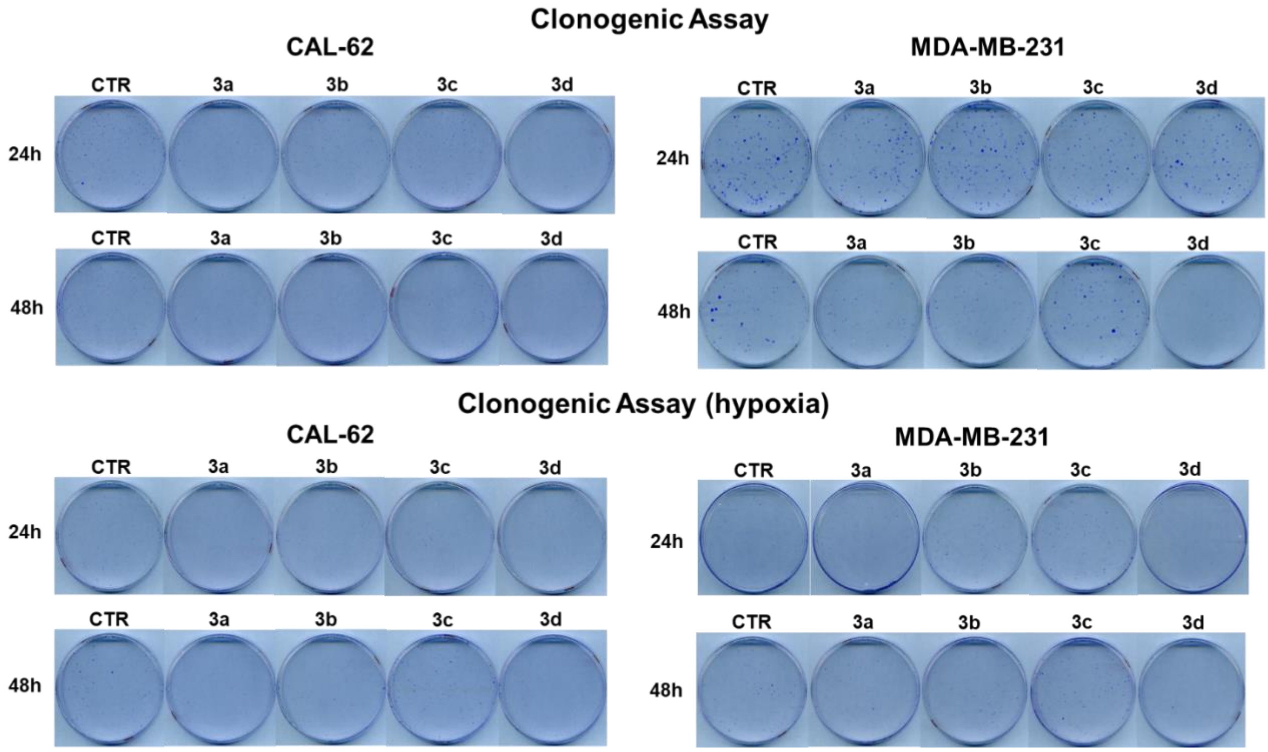

Figure S2. Representative plate images of the colony formation assay for CAL-62 and MDA-MB-231 cells treated with the SIRT3 activators **3a-d** at 50 μM for 24 and 48 h.

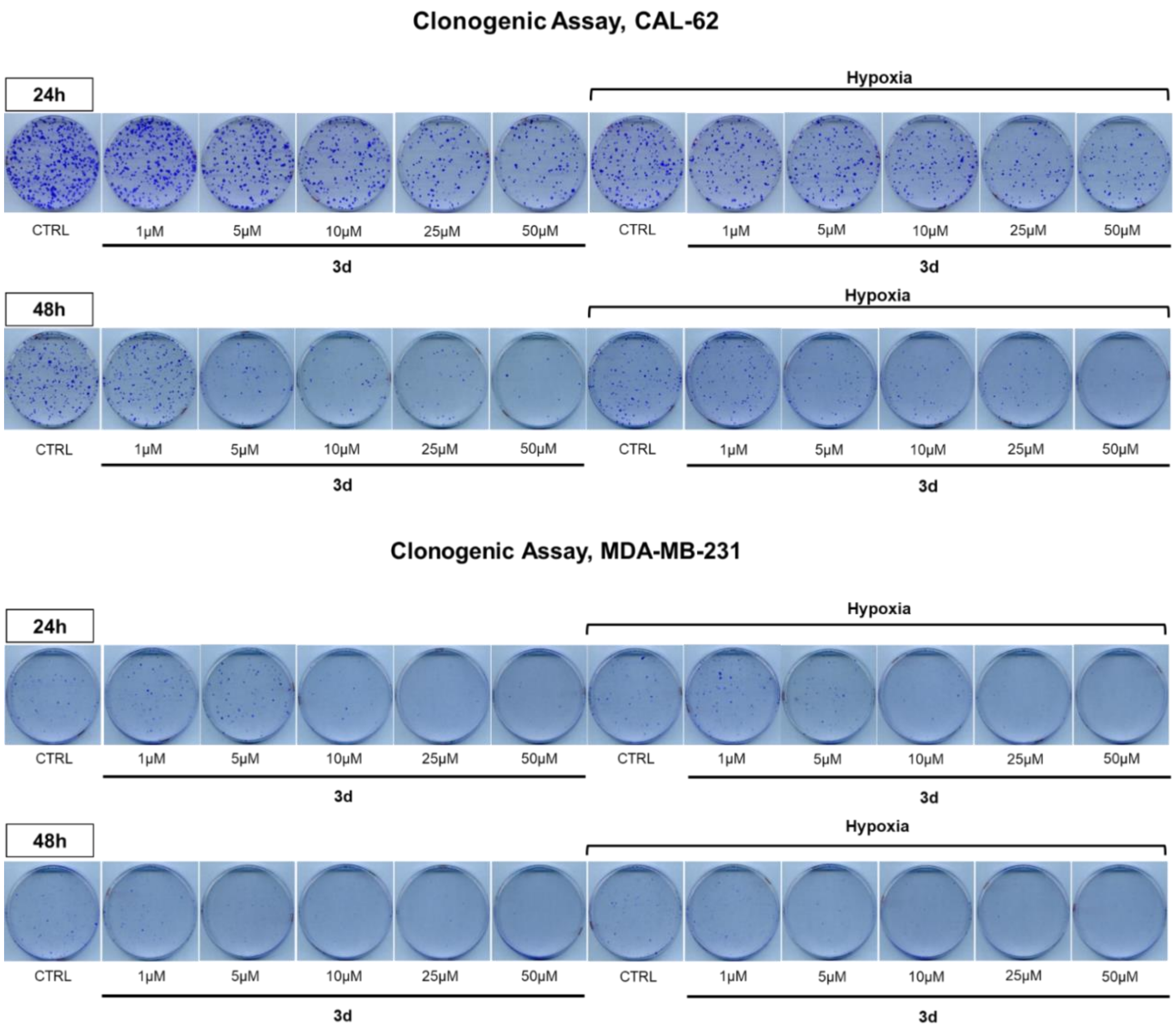

Figure S3. Representative plate images of the colony formation assay for CAL-62 and MDA-MB-231 cells treated with increasing dose (1, 5, 10, 25 and 50 μM) of the SIRT3 activator **3d** for 24 and 48 h.

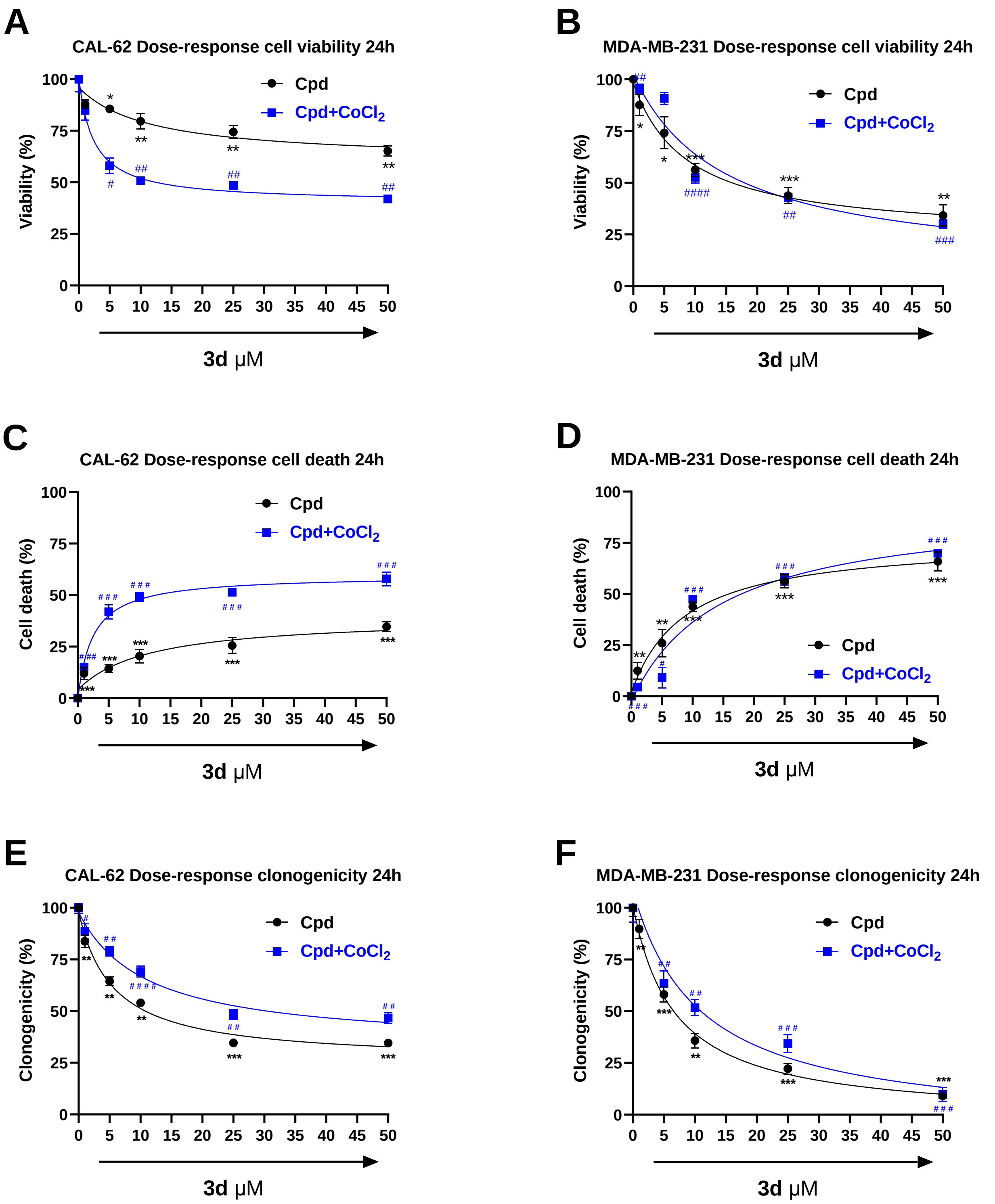

Figure S4. Dose-dependent effects of compound **3d** on cell viability, cell death and clonogenicity in CAL-62 (A, C, E) and MDA-MB-231 (B, D, F) cells upon 24 h of treatment. p-values were obtained using a Student's t-test (*p < 0.05, **p < 0.01, ***p < 0.001, ****p < 0.0001); $^{\#}p < 0.05$, $^{\#\#}p < 0.01$, $^{\#\#\#}p < 0.001$, $^{\#\#\#\#}p < 0.0001$) for three independent experiments.

Table S3. List of Primers used in gene expression analysis.

OLIGO FORWARD	OLIGO REVERSE
L32: GGAGCGACTGCTACGGAAG	L32: GATACTGTCCAAAAGGCTGGAA
SLUG: ATGAGGAATCTGGCTGCTGT	SLUG: CTCTCTCTGTGGGTGTGTGT
ZEB1: CAGGGAGGAGCAGTGAAAGA	ZEB1: CTCTTCAGGTGCCTCAGGAA
MMP2: ATGCCGCCTTTAACTGGAG	MMP2: GGAAAGCCAGGATCCATTTT
SNAIL: CACTATGCCGCGCTCTTTC	SNAIL: GCTGGAAGGTAAACTCTGGATTAGA
MMP9: CTACTGTGCCTTTGAGTCC	MMP9: AGAGAATCGCCAGTACTTCC
COL1A2: TGCAAGAACAGCATTGCATAC	COL1A2: GGCAGGCGAGATGGCTTATTTGTT

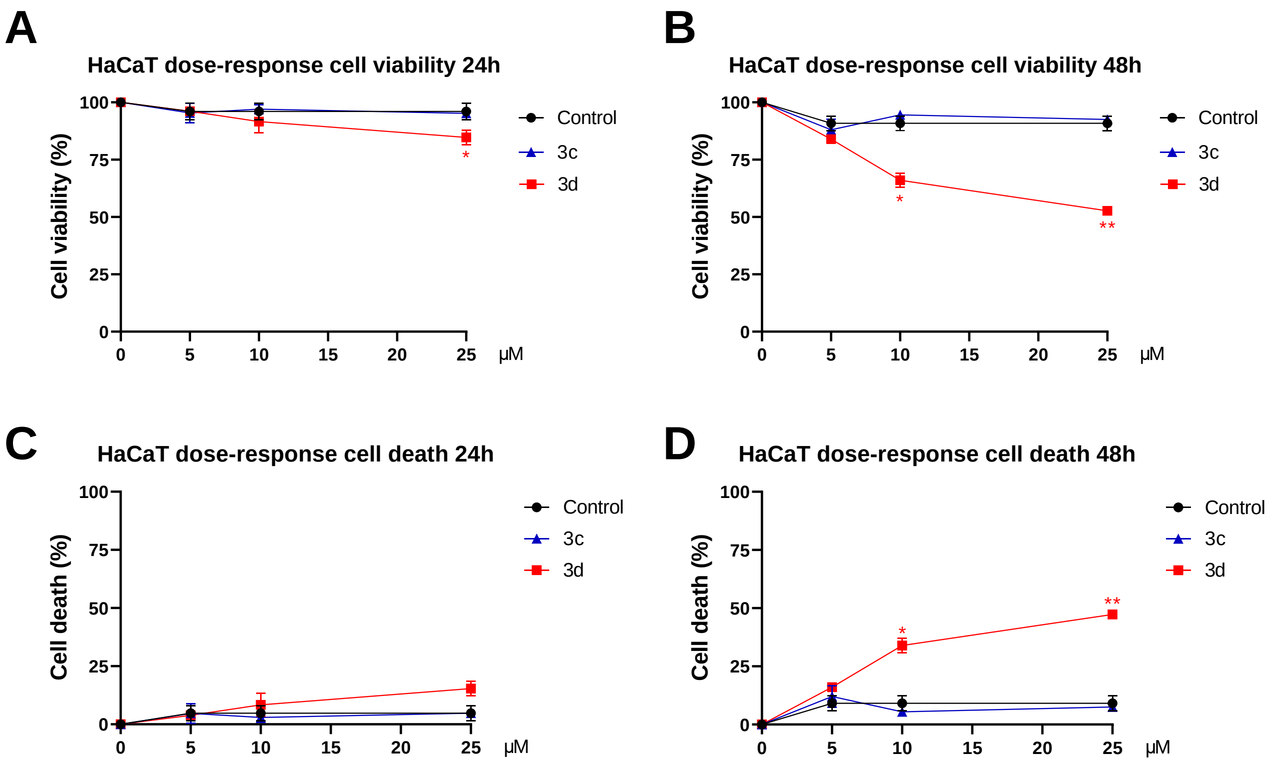

Figure S5. Dose-dependent effects of compounds **3c** and **3d** on cell viability (A, B) and cell death induction (C, D) in non-cancer HaCaT cells upon 24 h (A, C) and 48 h (B, D) of treatment. p-values were obtained using a Student's t-test (*p < 0.05, **p < 0.01) for three independent experiments.

**A)** Cell viability (%) T24 cpd **3d**			
Cpd conc.	CAL-62	MDA-MB-231	HaCaT
5 µM	85,7	74,1	96,1
10 µM	79,6	56,2	91,6
25 µM	74,4	42,8	84,7

**B)** Cell viability (%) T48 cpd **3d**			
Cpd conc.	CAL-62	MDA-MB-231	HaCaT
5 µM	78,3	51,7	83,9
10 µM	63,5	36,3	66,1
25 µM	33,8	21,1	52,7

**C)** Cell death (%) T24 cpd **3d**			
Cpd conc.	CAL-62	MDA-MB-231	HaCaT
5 µM	14,3	25,9	3,9
10 µM	20,4	43,8	8,4
25 µM	25,6	57,2	15,3

**D)** Cell death (%) T48 cpd **3d**			
Cpd conc.	CAL-62	MDA-MB-231	HaCaT
5 µM	21,7	48,3	16,1
10 µM	36,6	63,6	33,9
25 µM	66,1	78,8	47,3

Tables S4 (A-D). Quantification of living (A, B) and death cells (C, D) upon 24 h (A, C) and 48 h (B, D) of treatment relative to three independent experiments. Cell lines are indicated.
